# Supplementary material for: Asteroid shower on the Earth-Moon system immediately before the Cryogenian period revealed by KAGUYA
Source: Nat Commun. 2020 Jul 21;11:3453. doi: 10.1038/s41467-020-17115-6 (PMC7374575; doi:10.1038/s41467-020-17115-6)
Supplement: Supplementary file 1 — Supplementary Information [file 41467_2020_17115_MOESM1_ESM.pdf]

Supplementary Information for

Asteroid shower on the Earth-Moon system immediately before the Cryogenian period revealed by KAGUYA

By Terada et al.

All calibrated ages in Supplementary Information are based on a constant flux model.

Byrgius A

Diameter: 18.7 km

Number of craters: 366   Counting area: 148 km^2

N(1)=$3.83 \times 10^{-5} \pm 1.98 \times 10^{-6}$ km^{-2}, N(10)=$9.71 \times 10^{-9} \pm 5.03 \times 10^{-9}$ km^{-2}

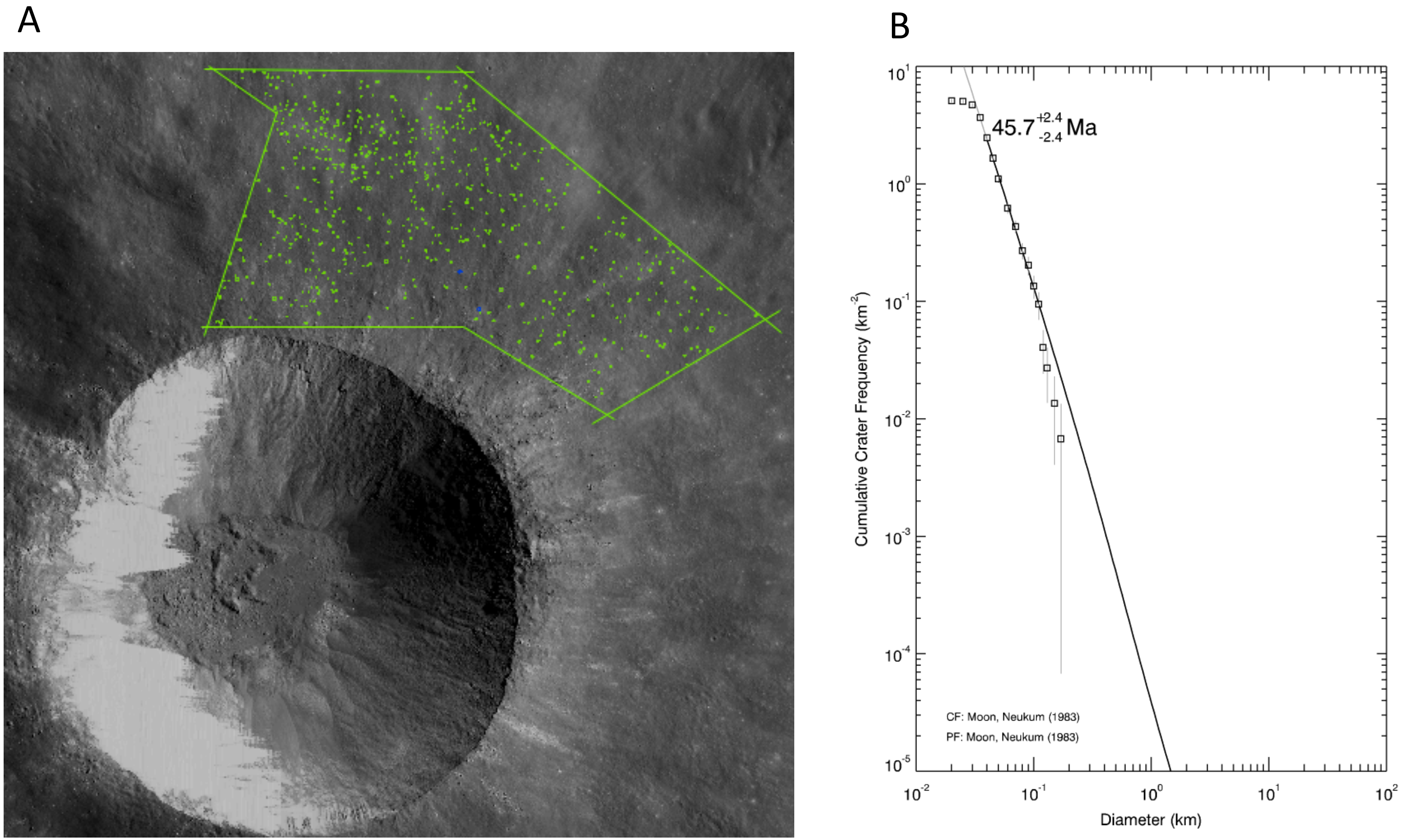

Supplementary Figure 1: The Terrain Camera image and the cumulative size-frequency distribution of Byrgius A

Tycho

Diameter: 83.2 km

Number of craters: 1092 Counting area: 2147 km^2

N(1)=$4.85 \times 10^{-5} \pm 2.44 \times 10^{-6}$ km^{-2}, N(10)=$1.23 \times 10^{-7} \pm 6.20 \times 10^{-9}$ km^{-2}

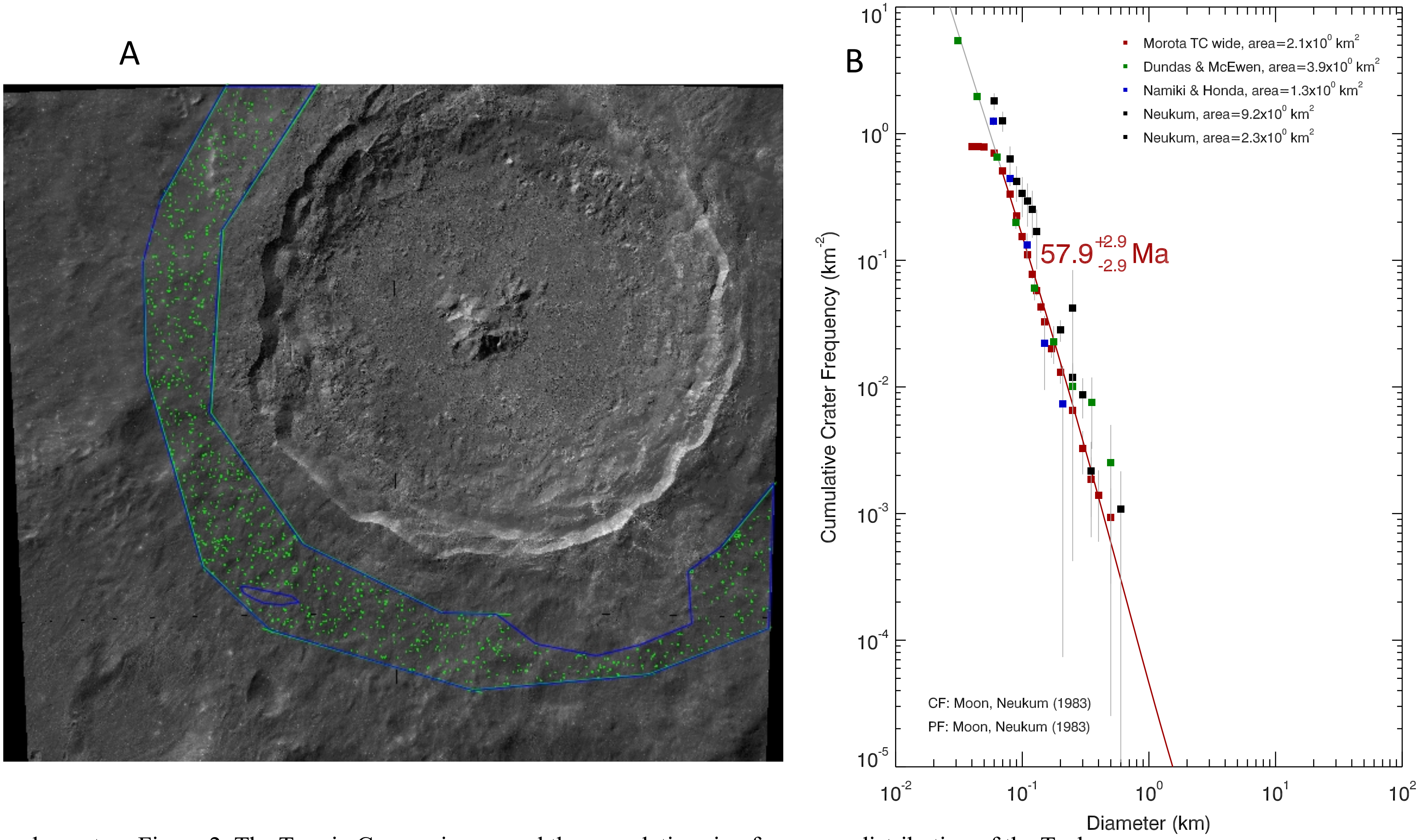

Supplementary Figure 2: The Terrain Camera image and the cumulative size-frequency distribution of the Tycho

Proclus

Diameter: 27.2 km

Number of craters: 101   Counting area: 415 km^2

N(1)=8.74×10^{-5}±8.61×10^{-6} km^{-2}, N(10)=2.22×10^{-7}±2.18×10^{-8} km^{-2}

A

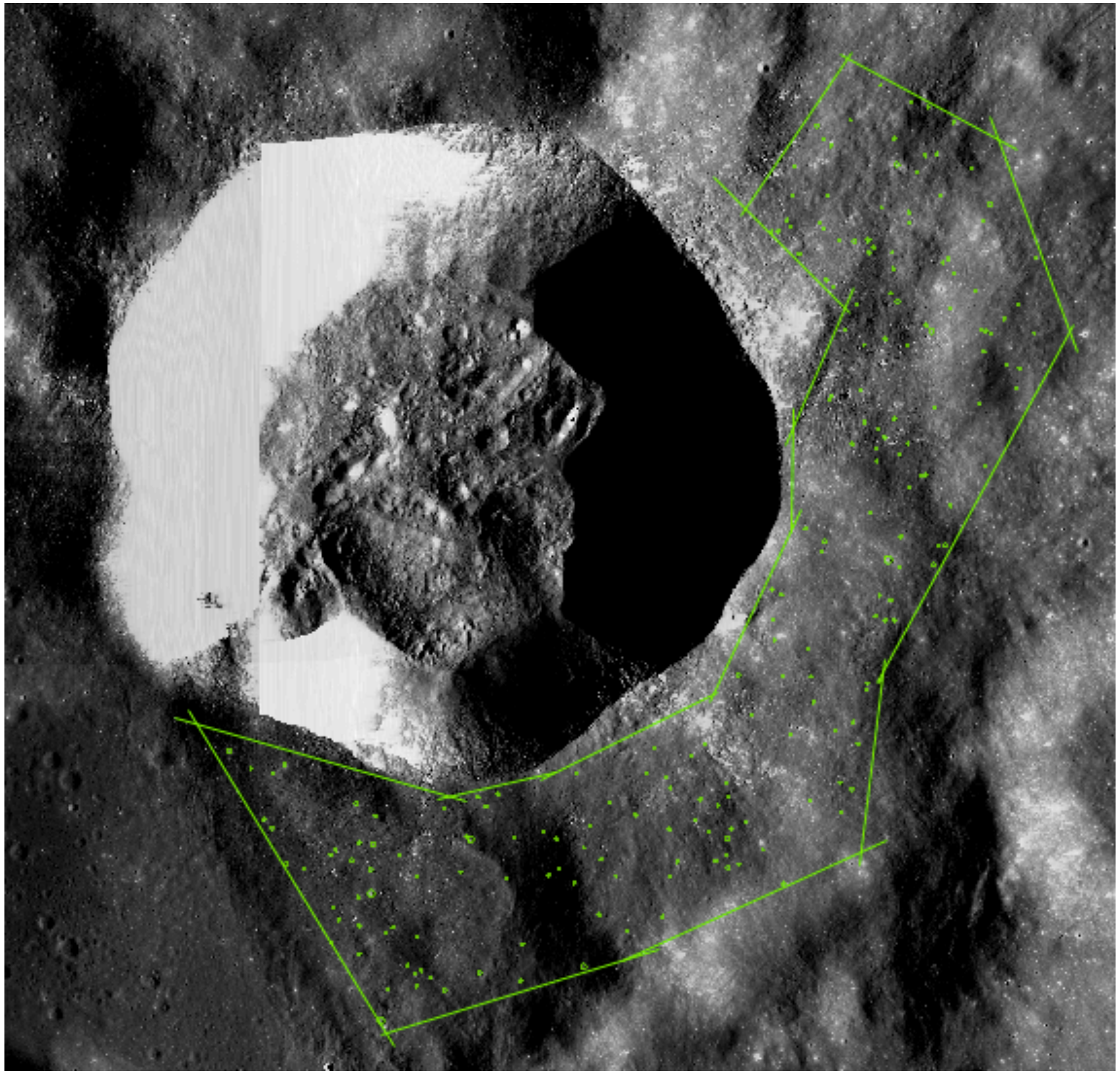

B

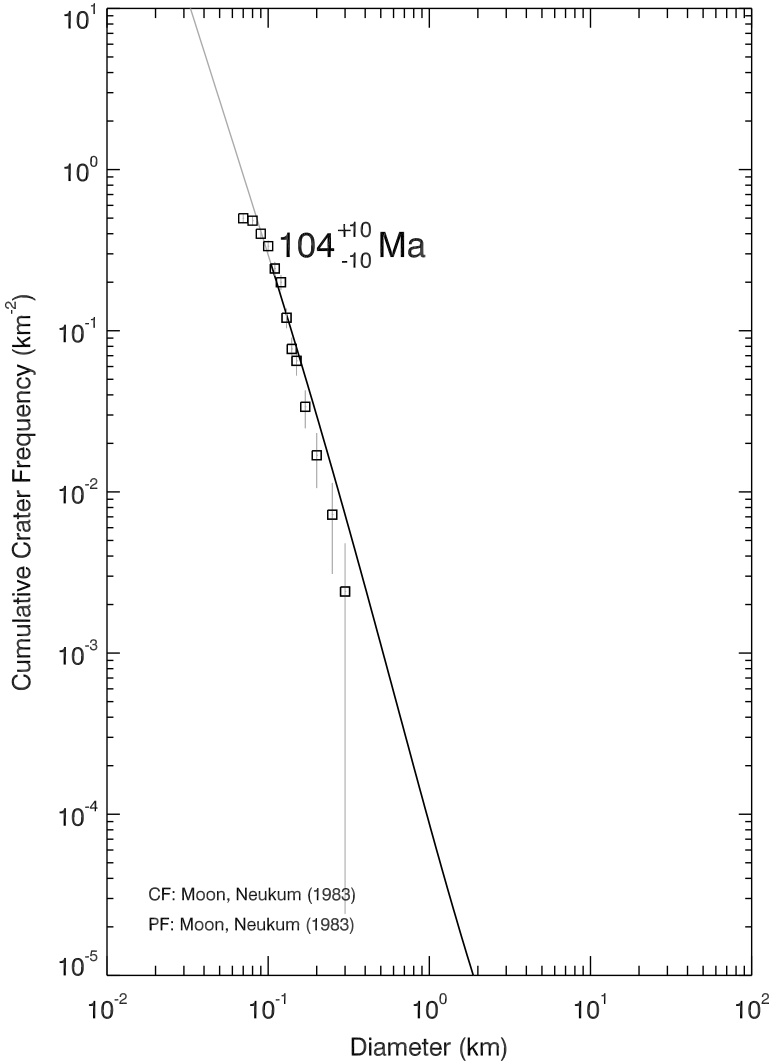

Supplementary Figure 3: The Terrain Camera image and the cumulative size-frequency distribution of Proclus

Aristarchus

Diameter: 40.0 km

Number of craters: 153 Counting Area: 287 km^2

$N(1)=1.10\times10^{-4}\pm8.84\times10^{-6}$ km^{-2}, $N(10)=2.87\times10^{-7}\pm2.24\times10^{-8}$ km^{-2}

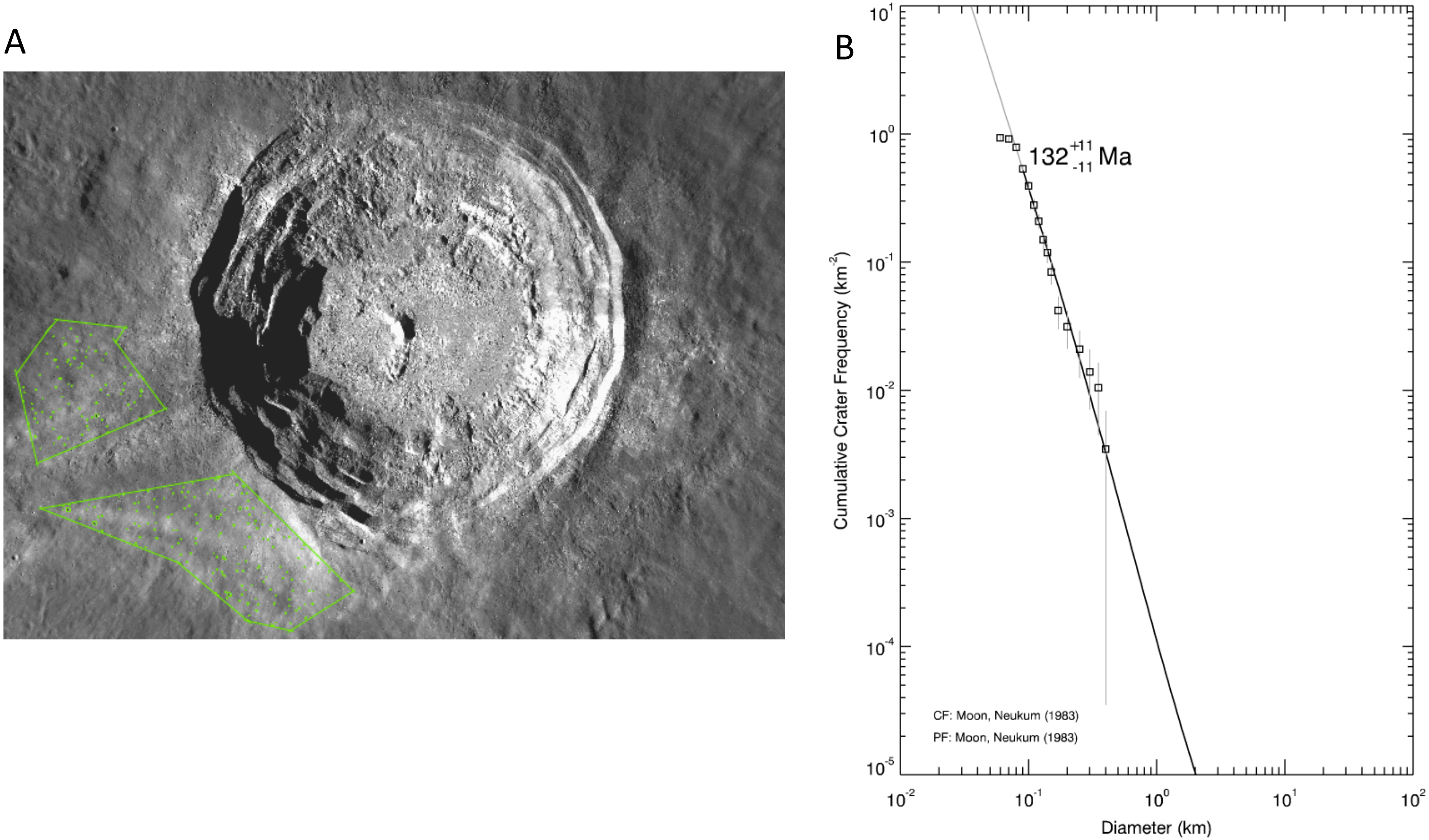

Supplementary Figure 4: The Terrain Camera image and the cumulative size-frequency distribution of Aristarchus

Olber A

Diameter: 41.8 km

Number of craters: 148 Counting area: 513 km^2

$N(1)=1.55\times10^{-4}\pm1.26\times10^{-5}$ km^{-2}, $N(10)=3.93\times10^{-7}\pm3.20\times10^{-8}$ km^{-2}

A

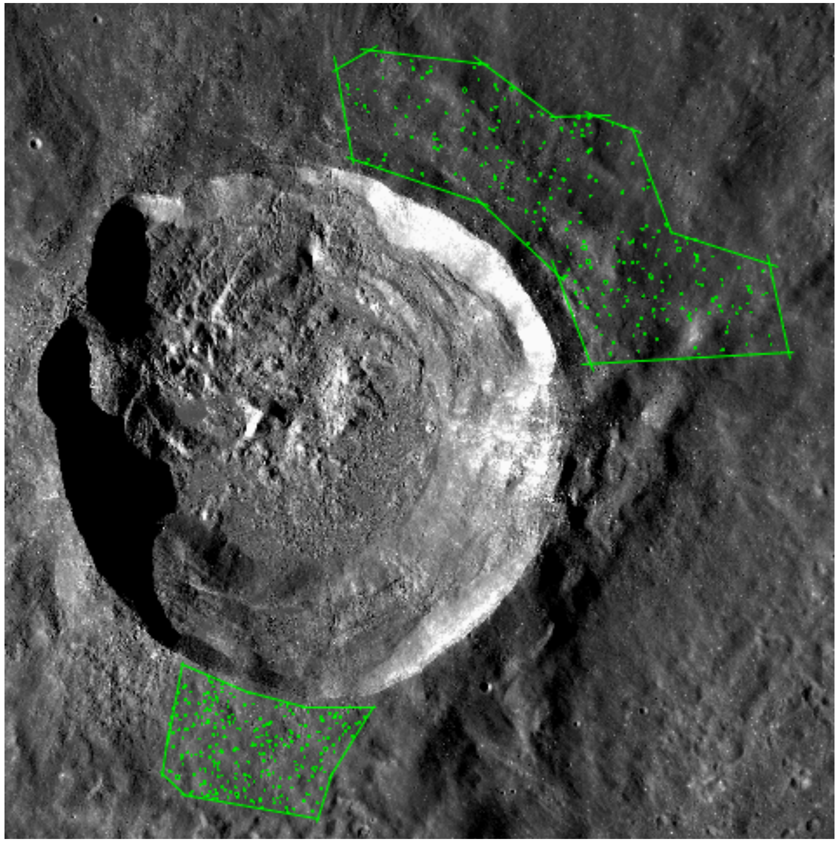

B

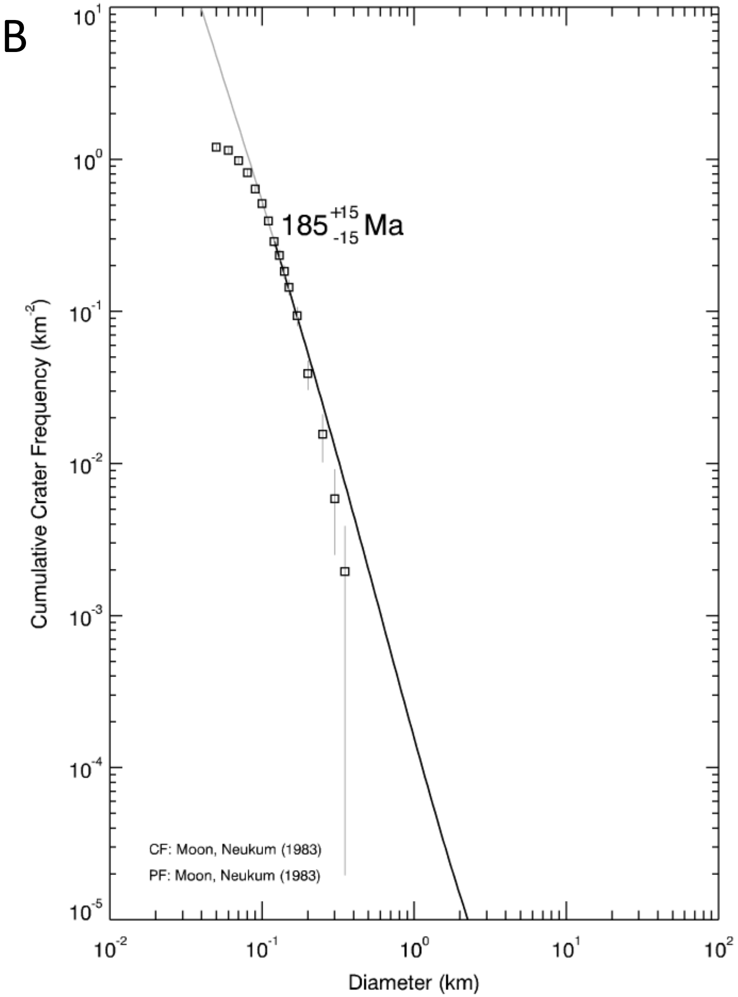

Supplementary Figure 5: The Terrain Camera image and the cumulative size-frequency distribution of Olber A

Petavius B

Diameter: 33.6 km

Number of craters: 43 Counting area: 392 km^2

N(1)=1.90$\times$10^{-4}$\pm$2.87$\times$10^{-5} km^{-2}, N(10)=4.82$\times$10^{-7}$\pm$7.27$\times$10^{-8} km^{-2}

A

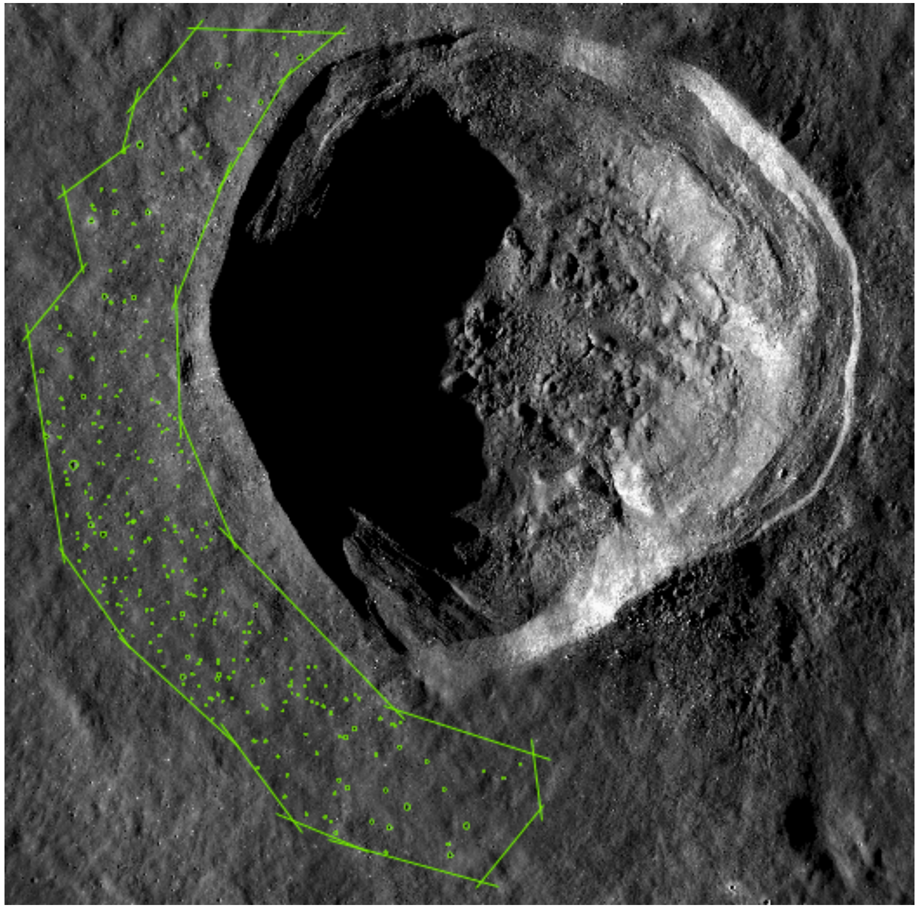

B

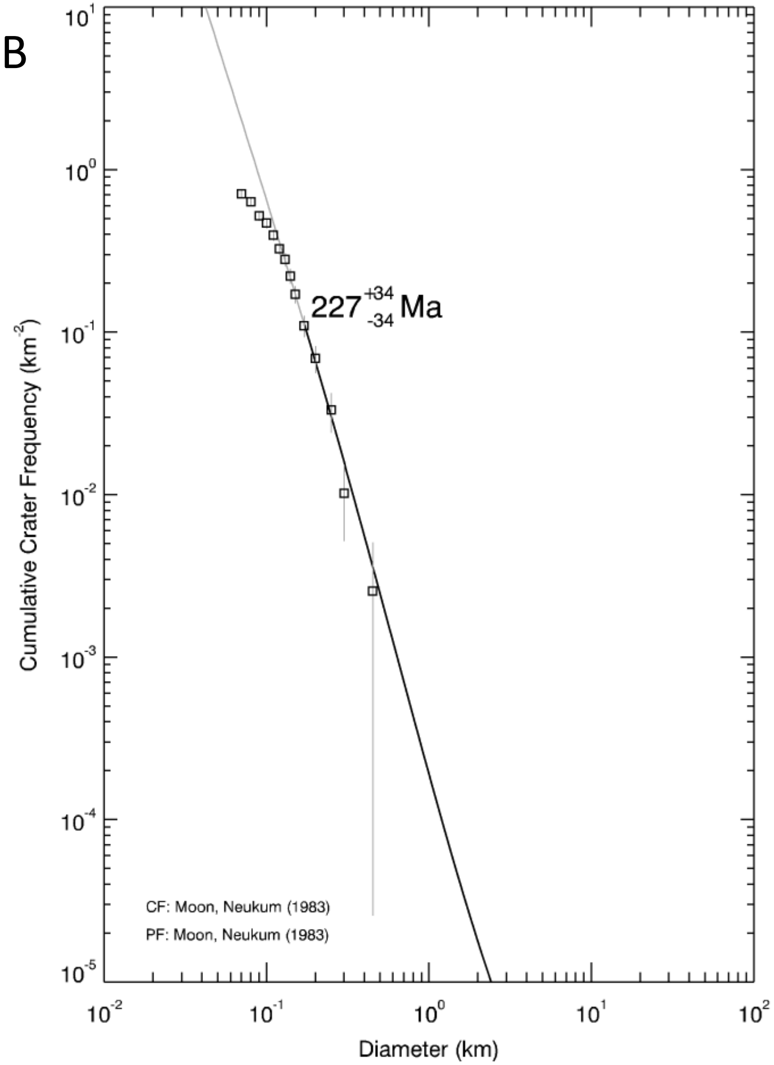

Supplementary Figure 6: The Terrain Camera image and the cumulative size-frequency distribution of Petavius B

Lalande

Diameter: 23.3 km

Number of craters: 148 Counting area: 128 km^2

N(1)=$2.51\times10^{-4}\pm2.04\times10^{-5}$ km^{-2}, N(10)=$6.37\times10^{-7}\pm5.18\times10^{-8}$ km^{-2}

A

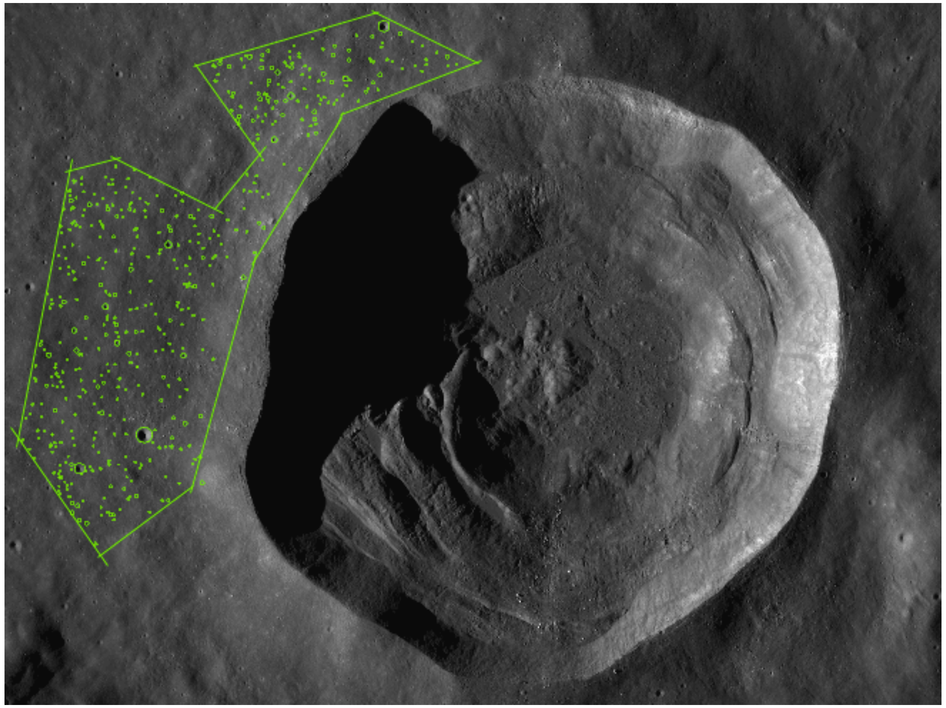

B

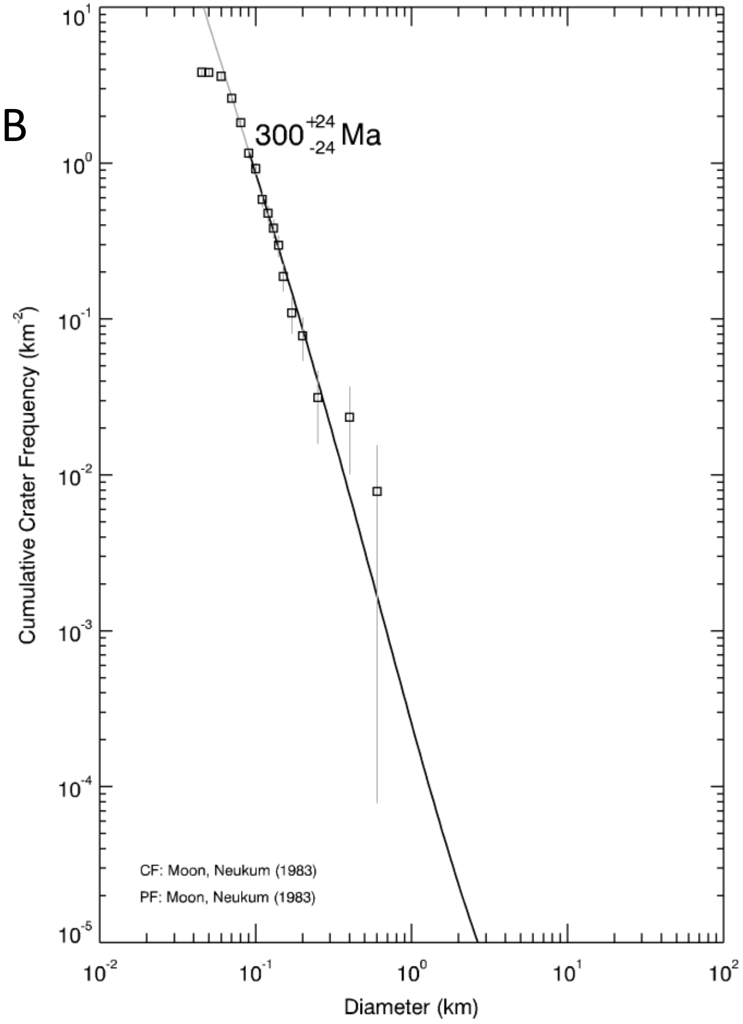

Supplementary Figure 7: The Terrain Camera image and the cumulative size-frequency distribution of Lalande

Kepler

Diameter: 29.9 km

Number of craters: 289 Counting area: 553 km^2

$N(1)=3.80\times10^{-4}\pm2.21\times10^{-5}$ km^{-2}, $N(10)=9.63\times10^{-7}\pm5.61\times10^{-8}$ km^{-2}

A

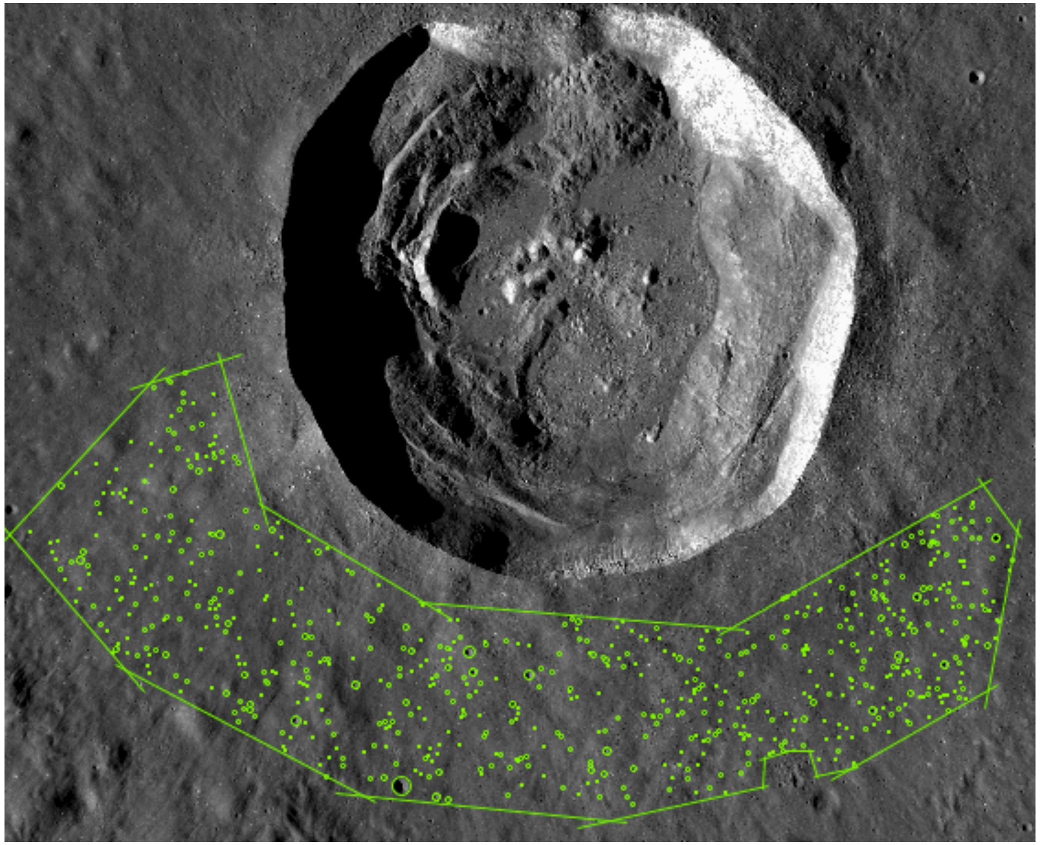

B

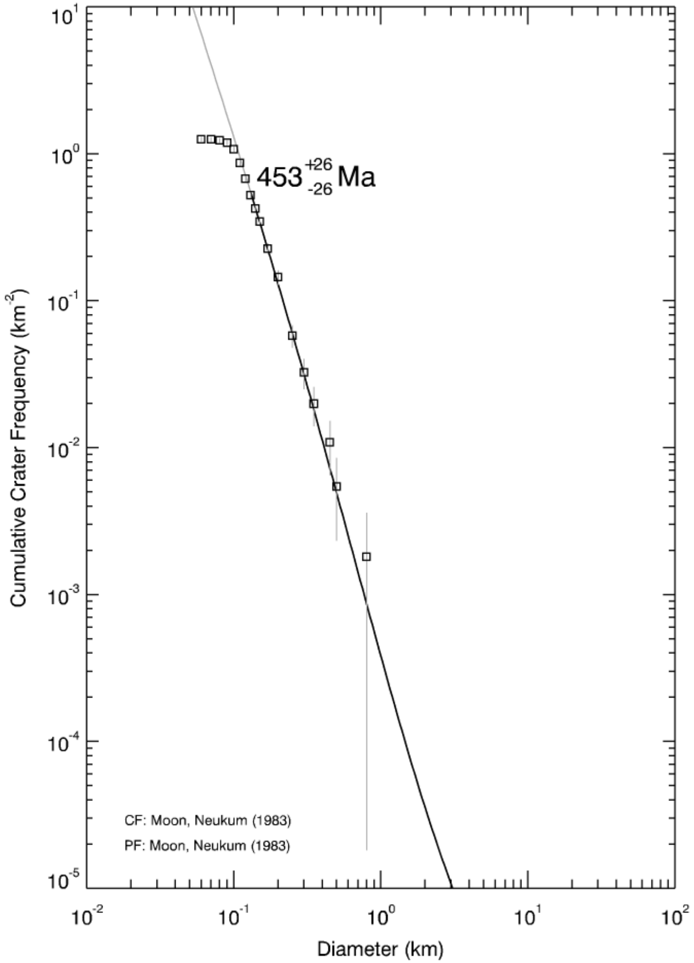

Supplementary Figure 8: The Terrain Camera image and the cumulative size-frequency distribution of Kepler

Godin

Diameter: 35.1 km

Number of craters: 307 Counting Area: 505 km^2

$N(1)=5.44\times10^{-4}\pm3.07\times10^{-5}\ km^{-2}$, $N(10)=1.38\times10^{-6}\pm7.79\times10^{-7}\ km^{-2}$

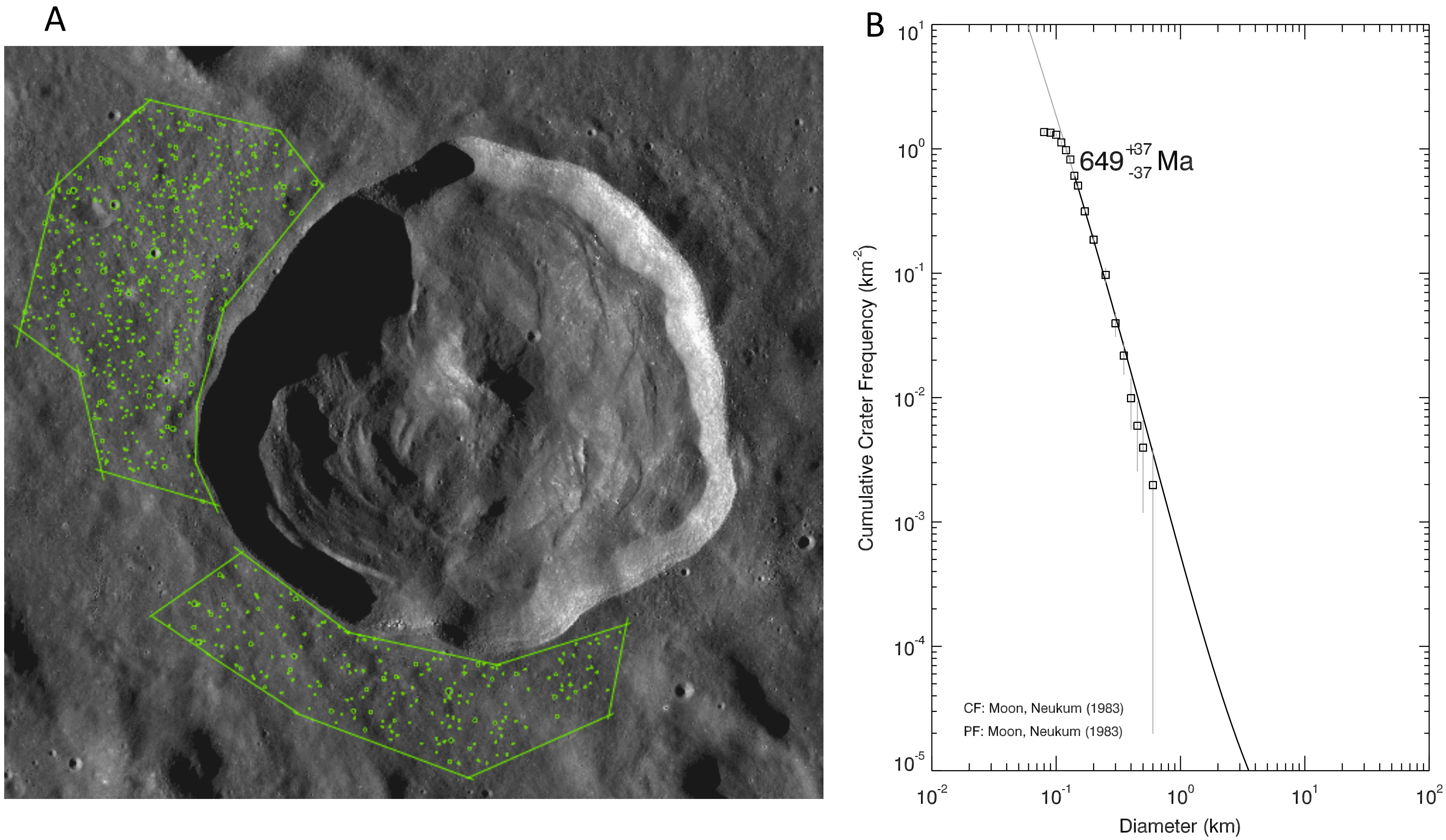

Supplementary Figure 9: The Terrain Camera image and the cumulative size-frequency distribution of Godin

Copernicus

Diameter: 93.1 km

Number of craters: 860 Counting area: 4370 km^2

$N(1)=5.56\times10^{-4}\pm1.88\times10^{-5}$ km^{-2}, $N(10)=1.41\times10^{-6}\pm4.76\times10^{-8}$ km^{-2}

A

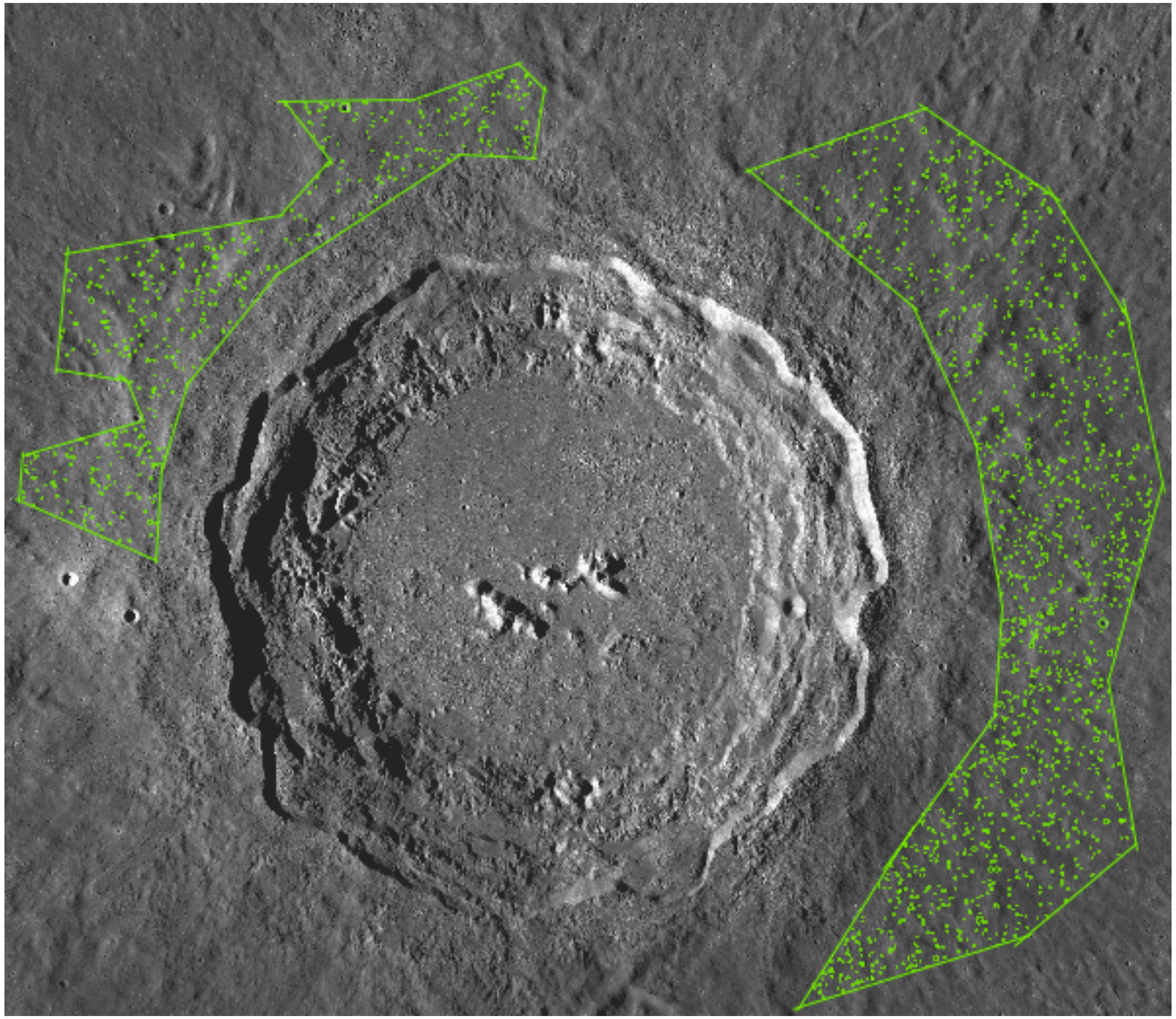

B

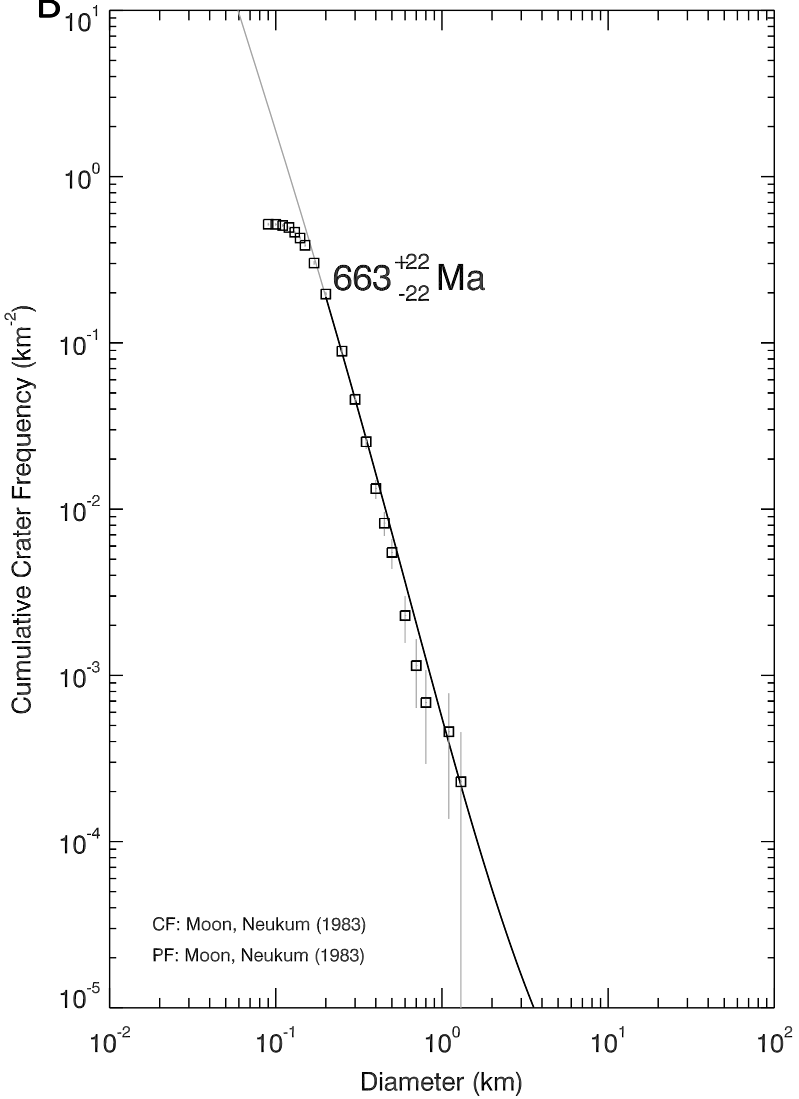

Supplementary Figure 10: The Terrain Camera image and the cumulative size-frequency distribution of Copernicus

Stevinus

Diameter: 70.3 km

Number of craters: 357 Counting area: 1623 km^2

$N(1)=6.42\times10^{-4}\pm3.36\times10^{-5}$ km^{-2}, $N(10)=1.63\times10^{-6}\pm8.53\times10^{-8}$ km^{-2}

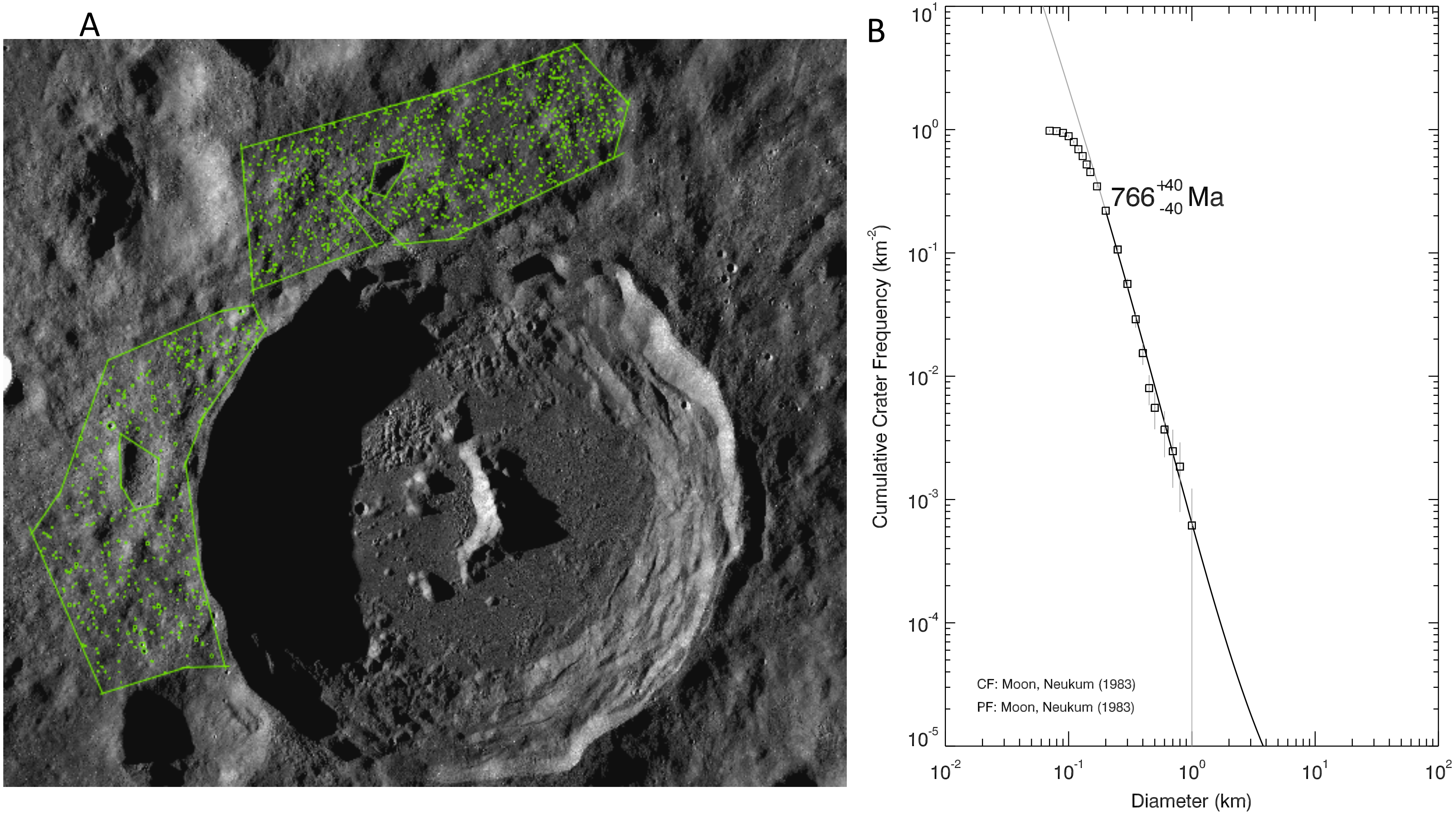

Supplementary Figure 11: The Terrain Camera image and the cumulative size-frequency distribution of Stevinus

Robinson

Diameter: 24.0 km

Number of craters: 38 Counting area: 112 km^2

$N(1)=9.76\times10^{-4}\pm1.57\times10^{-4}$ km^{-2}, $N(10)=2.47\times10^{-6}\pm3.97\times10^{-7}$ km^{-2}

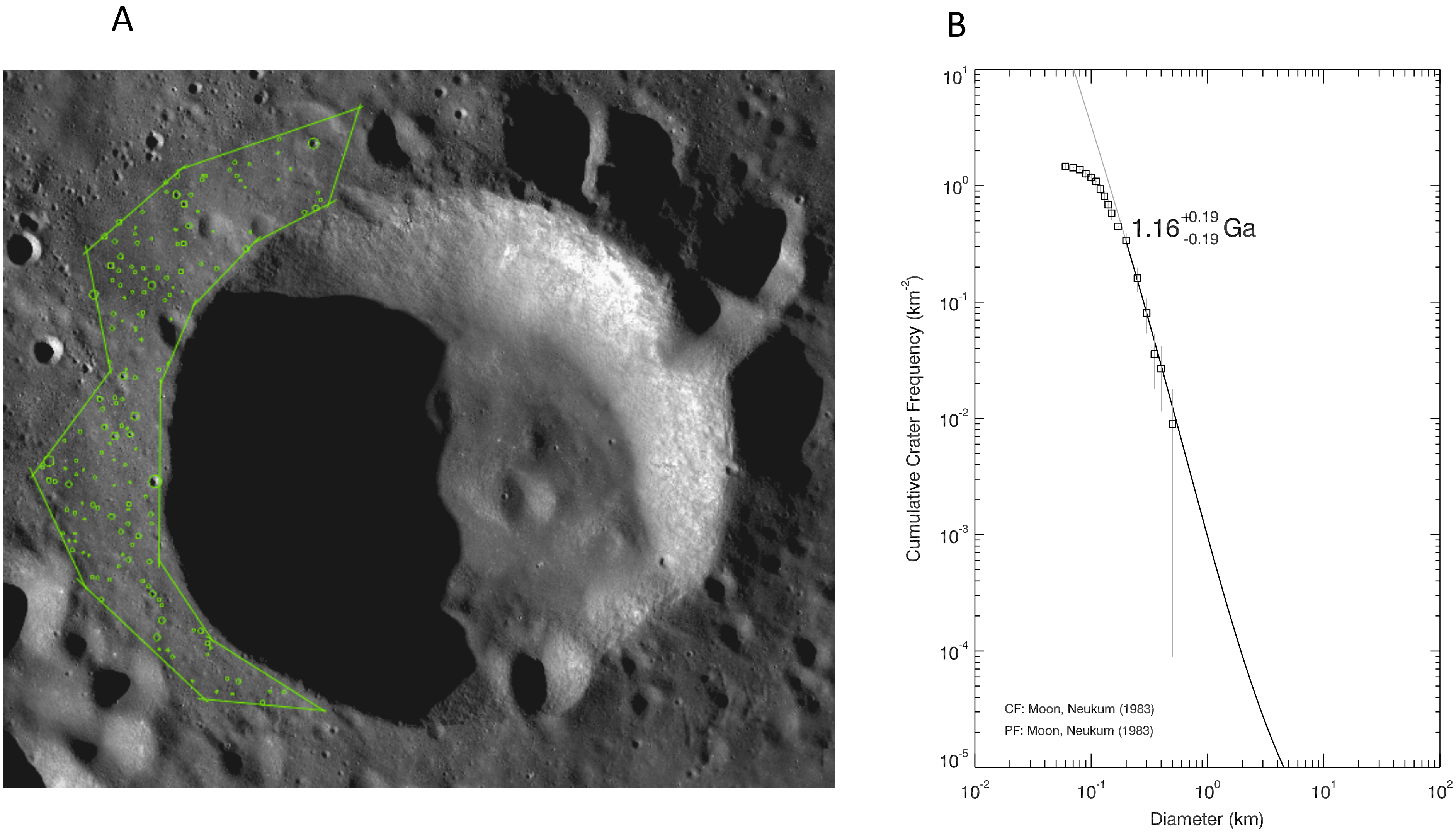

Supplementary Figure 12: The Terrain Camera image and the cumulative size-frequency distribution of Robinson

Harpalus

Diameter: 40.4 km

Number of craters: 368 Counting area: 612 km^2

N(1)=$1.03 \times 10^{-3} \pm 5.31 \times 10^{-5}$ km^{-2}, N(10)=$2.61 \times 10^{-6} \pm 1.35 \times 10^{-7}$ km^{-2}

A

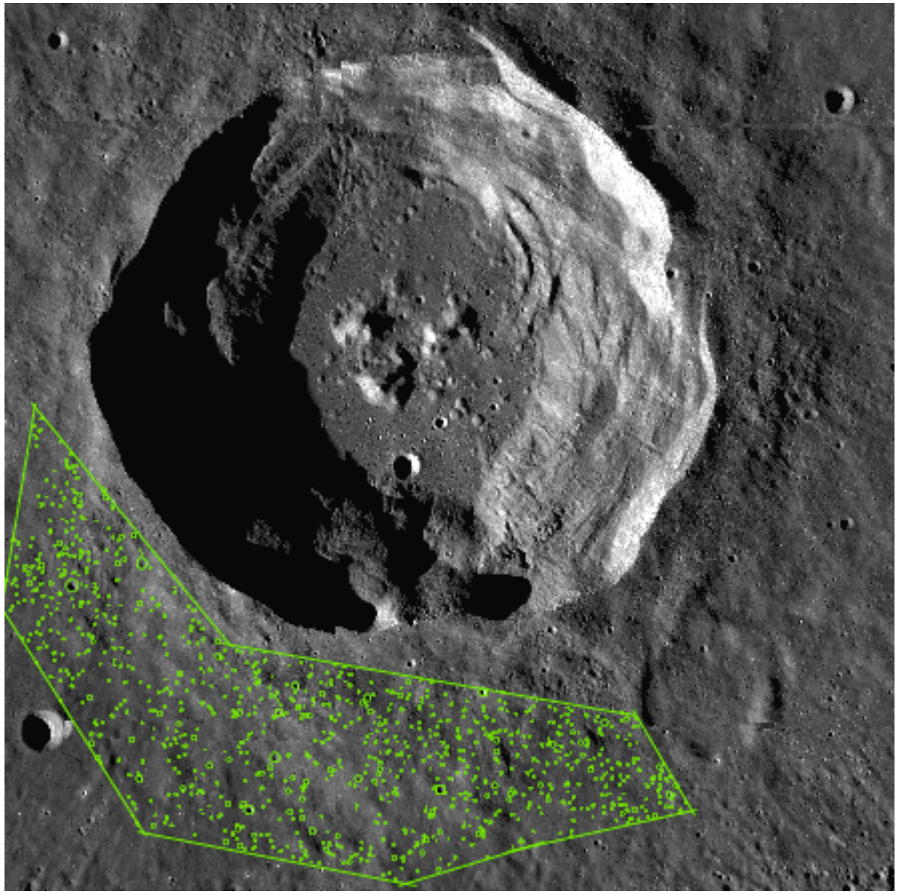

B

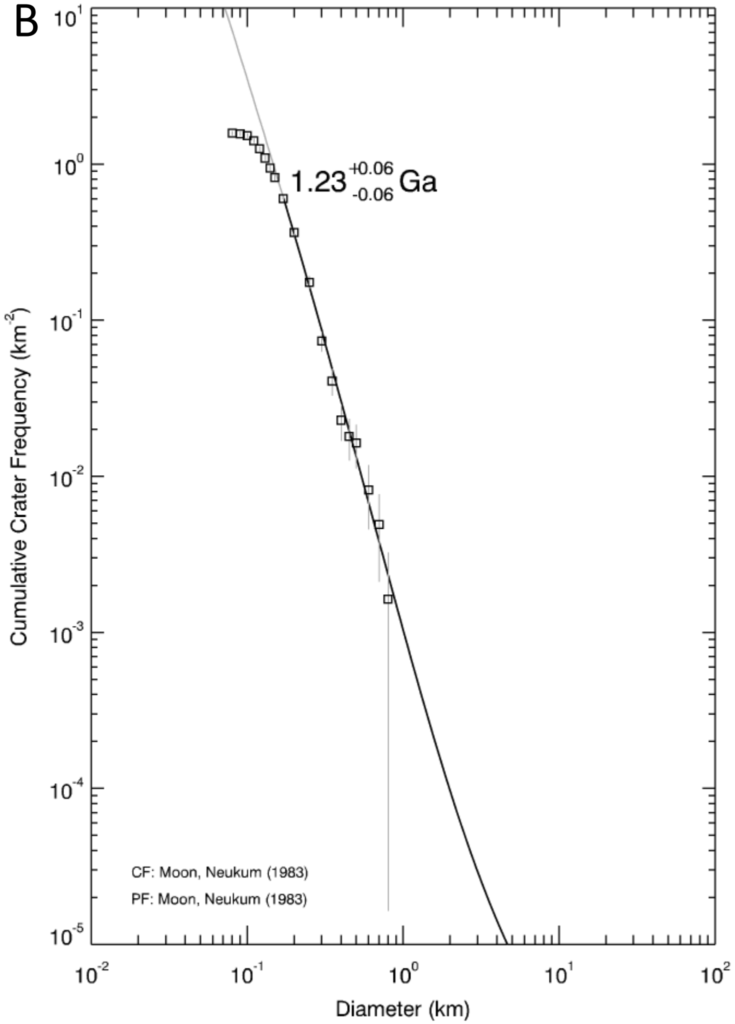

Supplementary Figure 13: The Terrain Camera image and the cumulative size-frequency distribution of Harpalus

Romer

Diameter: 41.7 km

Number of craters: 146   Counting area: 403 km^2

N(1)=$1.08 \times 10^{-3} \pm 8.87 \times 10^{-5}$ km^{-2}, N(10)=$2.74 \times 10^{-6} \pm 2.25 \times 10^{-7}$ km^{-2}

A

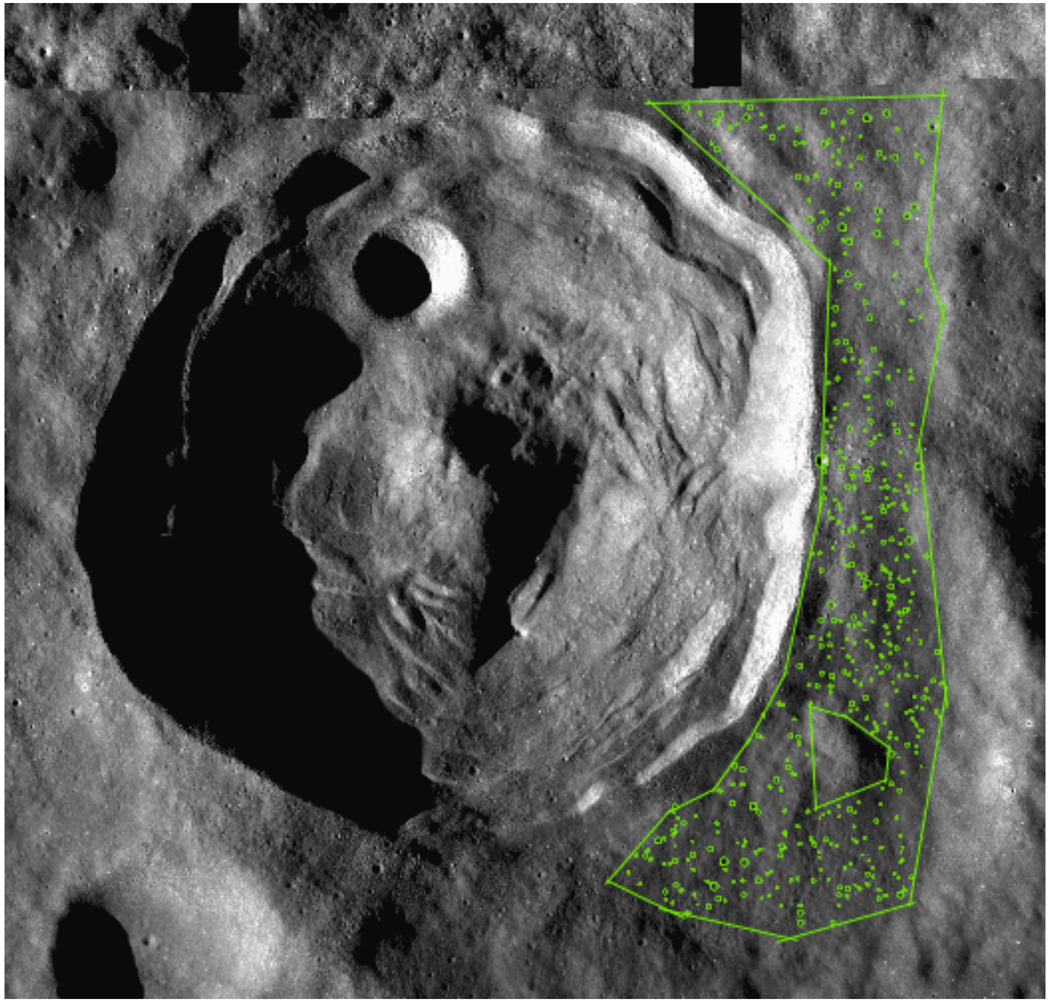

B

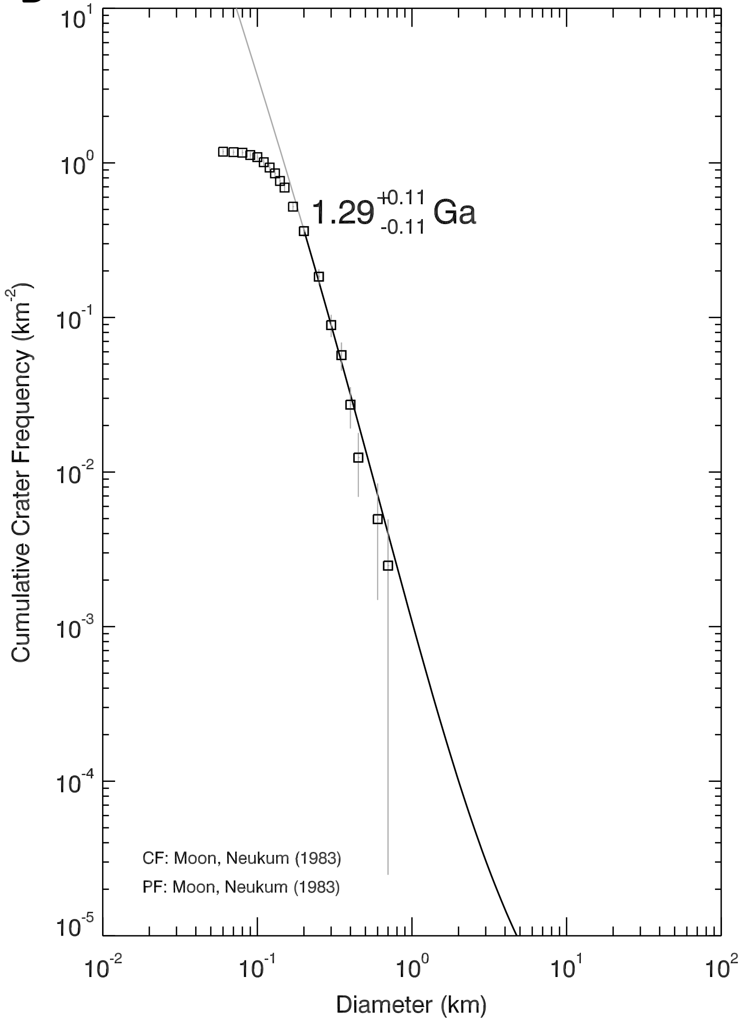

Supplementary Figure 14: The Terrain Camera image and the cumulative size-frequency distribution of Romer

Conon

Diameter: 20.8 km

Number of craters: 61 Counting area: 144 km^2

$N(1)=1.35\times10^{-3}\pm1.71\times10^{-4}$ km^{-2}, $N(10)=3.42\times10^{-6}\pm4.33\times10^{-7}$ km^{-2}

A

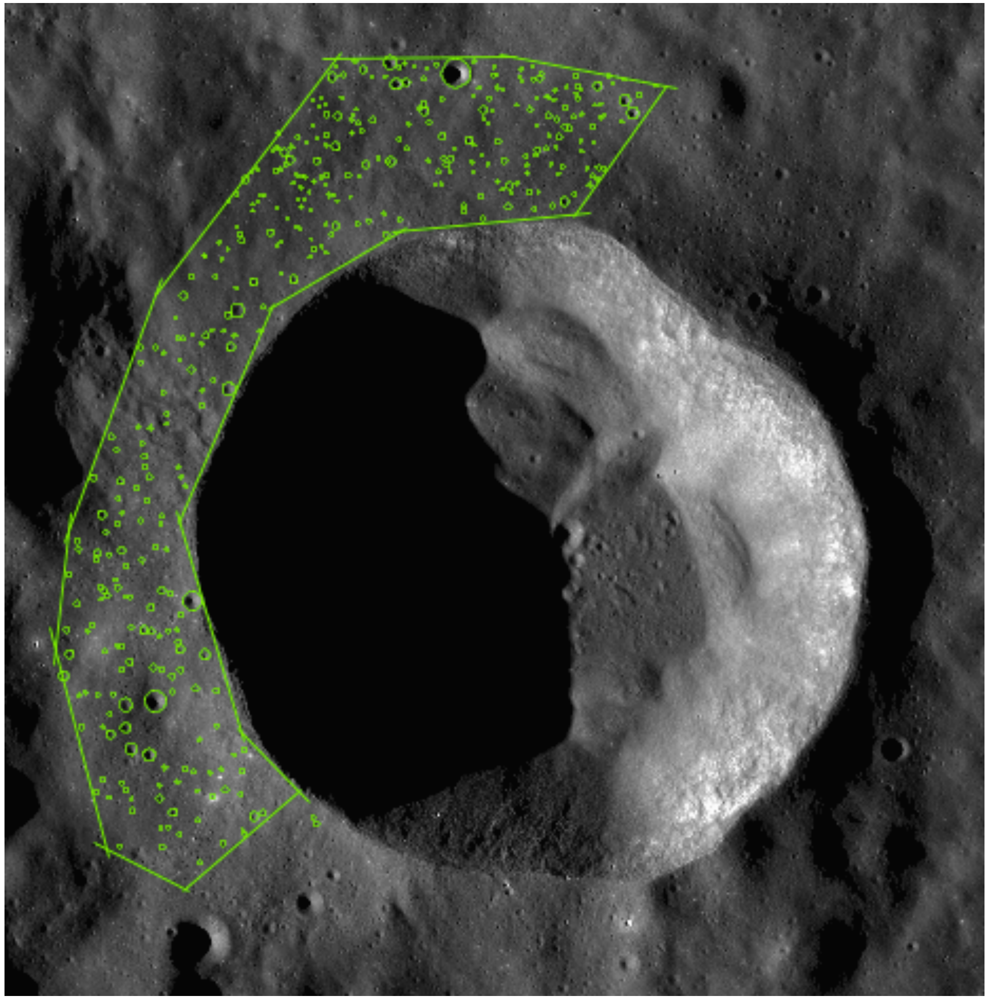

B

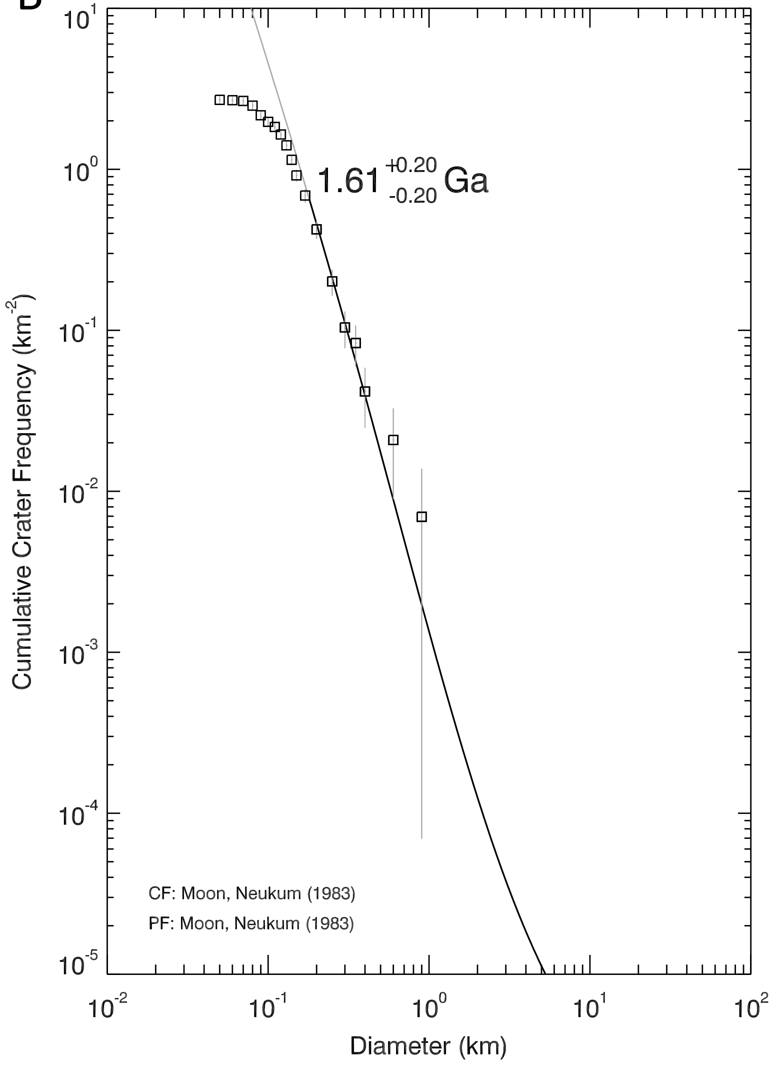

Supplementary Figure 15: The Terrain Camera image and the cumulative size-frequency distribution of Conon

Burg

Diameter: 39.1 km

Number of craters: 107   Counting area: 240 km^2

$N(1)=1.35\times10^{-3}\pm1.29\times10^{-4}$ km^{-2}, $N(10)=3.42\times10^{-6}\pm3.27\times10^{-7}$ km^{-2}

A

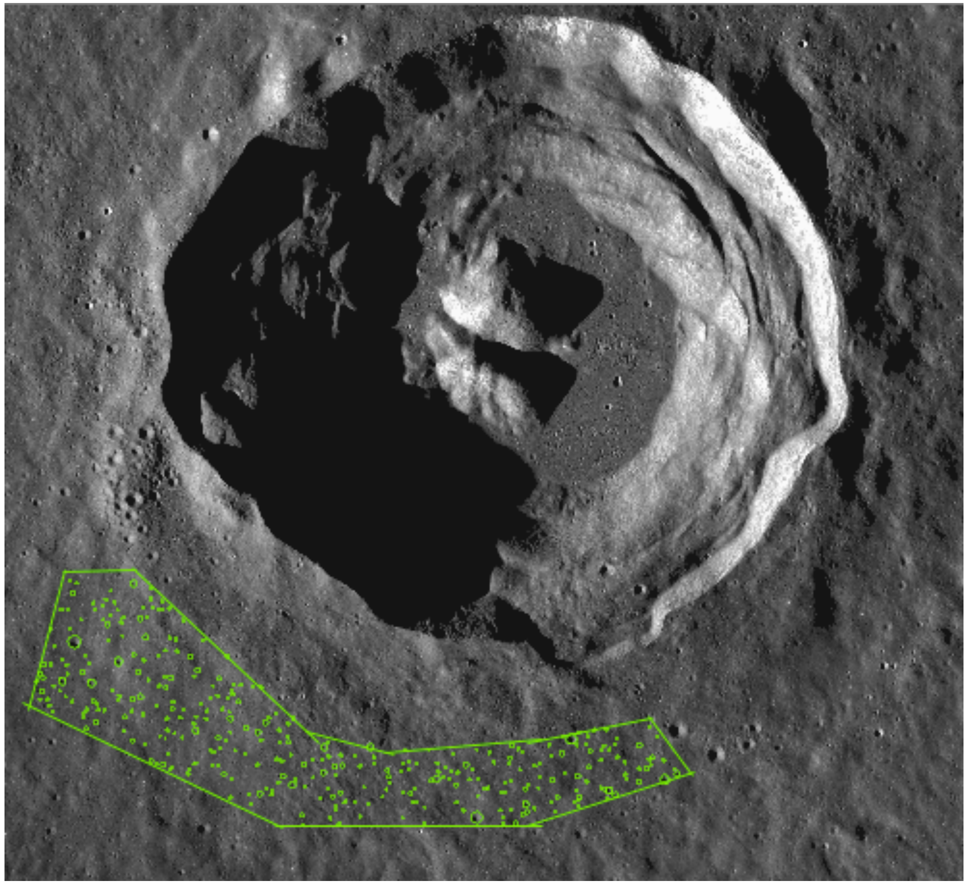

B

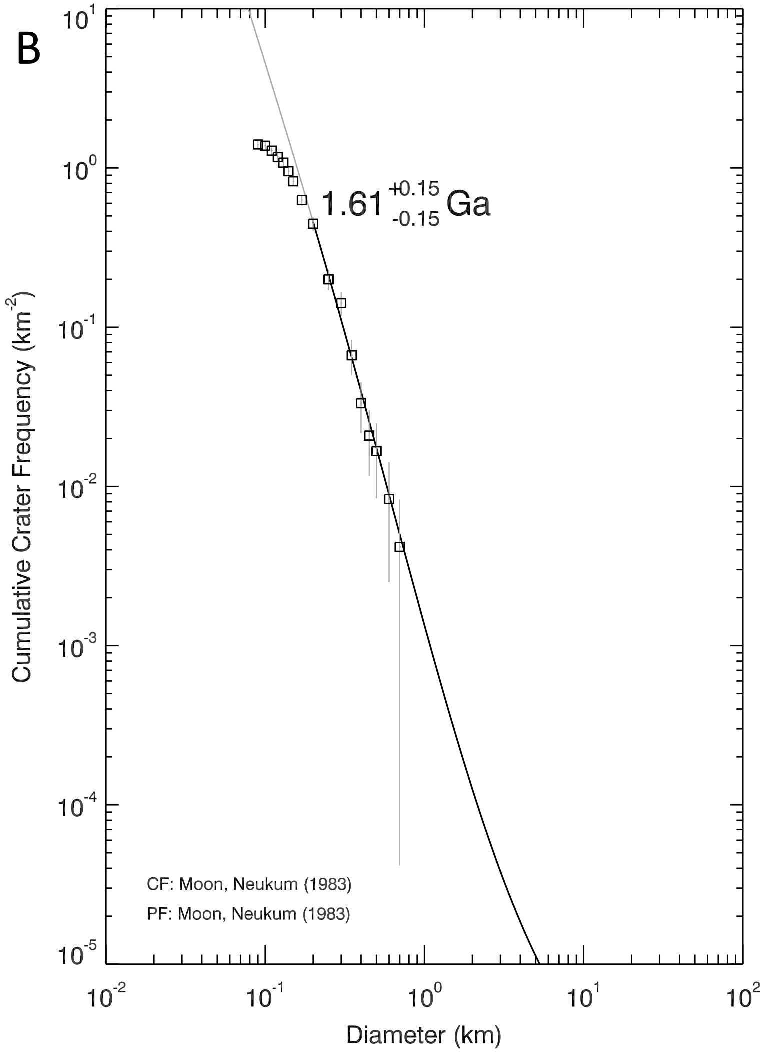

Supplementary Figure 16: The Terrain Camera image and the cumulative size-frequency distribution of Burg

Delisle

Diameter: 25.5 km

Number of craters: 93 Counting area: 497 km^2

$N(1)=1.35\times10^{-3}\pm1.39\times10^{-4}\ km^{-2}$, $N(10)=3.43\times10^{-6}\pm3.52\times10^{-7}\ km^{-2}$

A

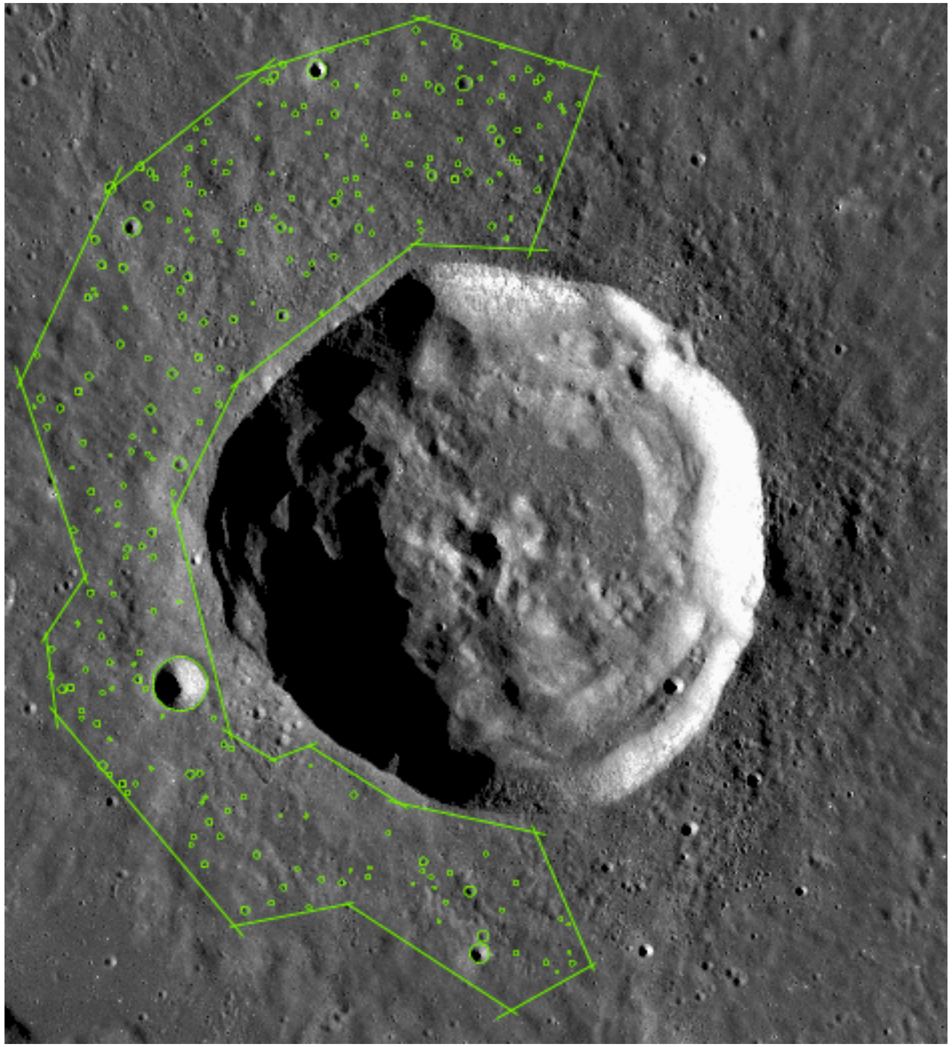

B

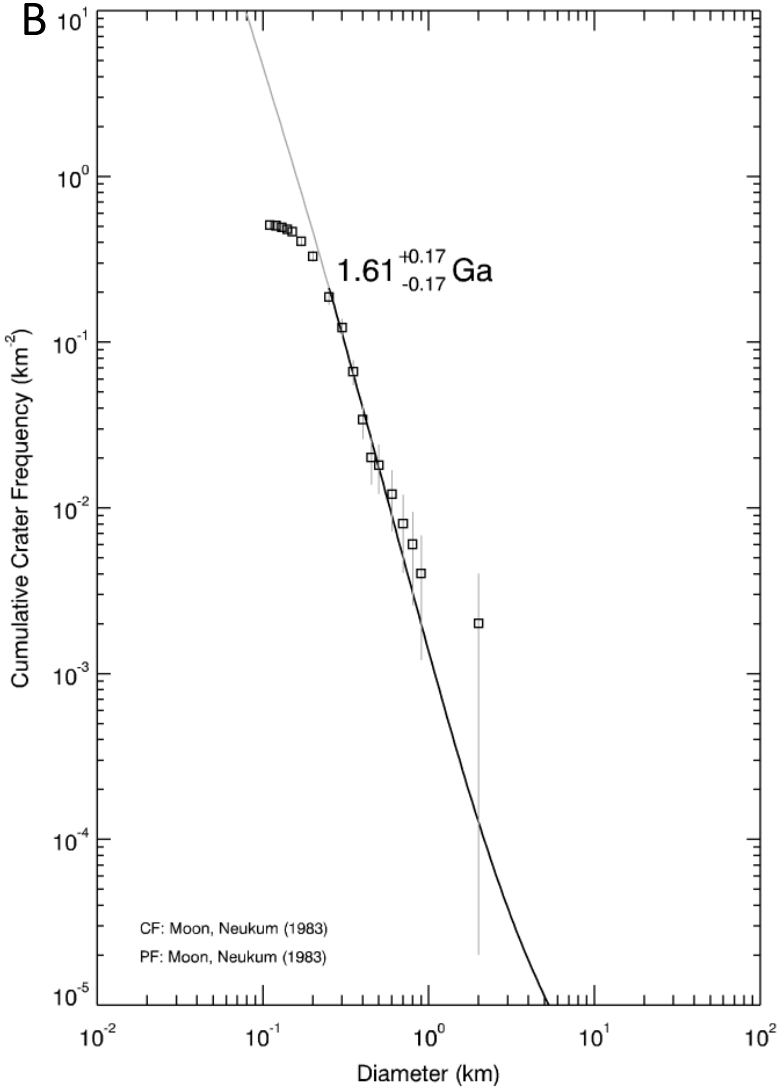

Supplementary Figure 17: The Terrain Camera image and the cumulative size-frequency distribution of Delisle

Triesnecker

Diameter: 25.3 km

Number of craters: 90 Counting area: 196 km^2

$N(1)=1.40\times10^{-3}\pm1.46\times10^{-4}$ km^{-2}, $N(10)=3.54\times10^{-6}\pm3.70\times10^{-7}$ km^{-2}

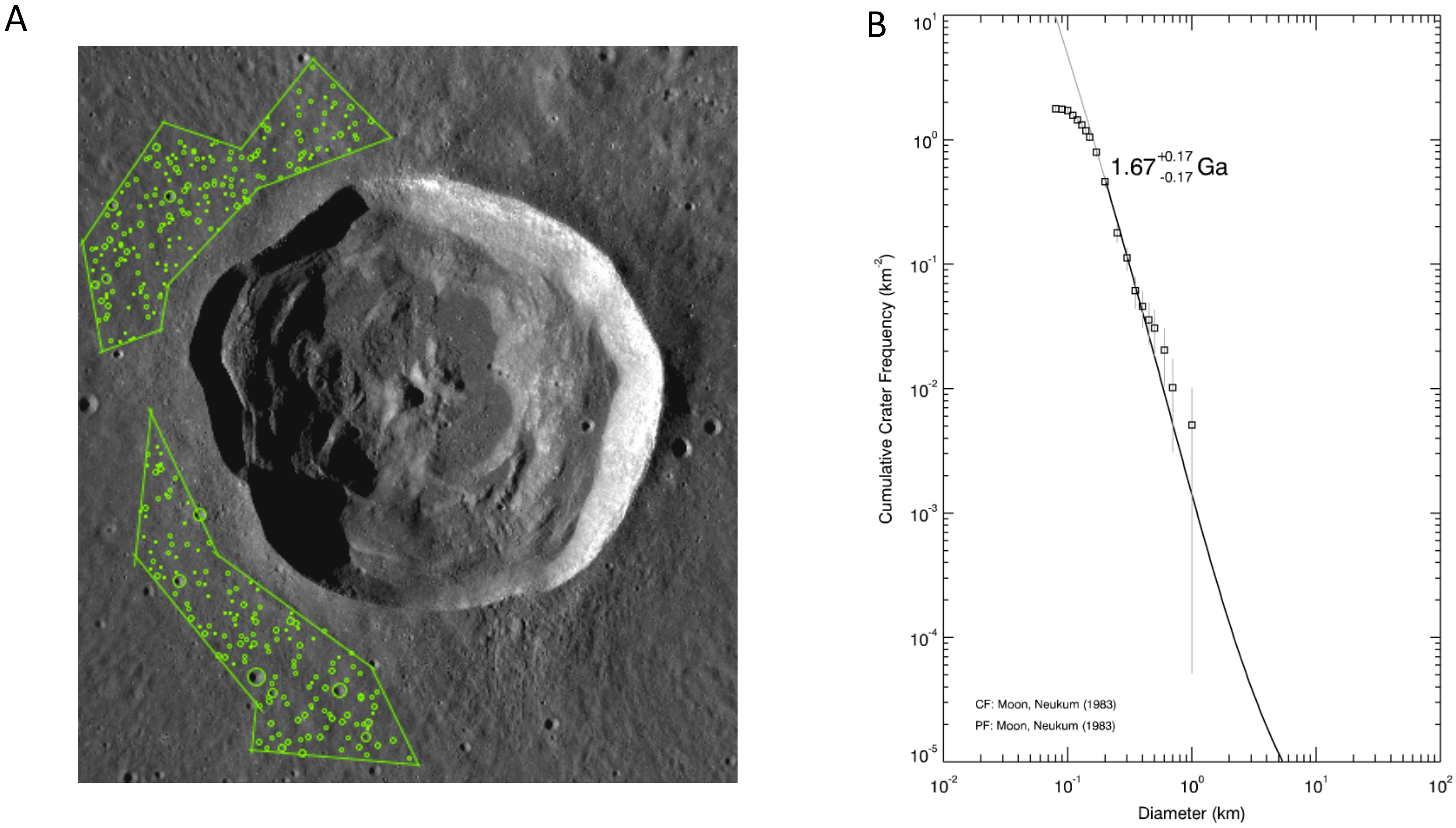

Supplementary Figure 18: The Terrain Camera image and the cumulative size-frequency distribution of Triesnecker

Eudoxus

Diameter: 67.1 km

Number of craters: 214 Counting area: 2040 km^2

N(1)=$1.50\times10^{-3}\pm1.01\times10^{-4}$ km^{-2}, N(10)=$3.80\times10^{-6}\pm2.57\times10^{-7}$ km^{-2}

A

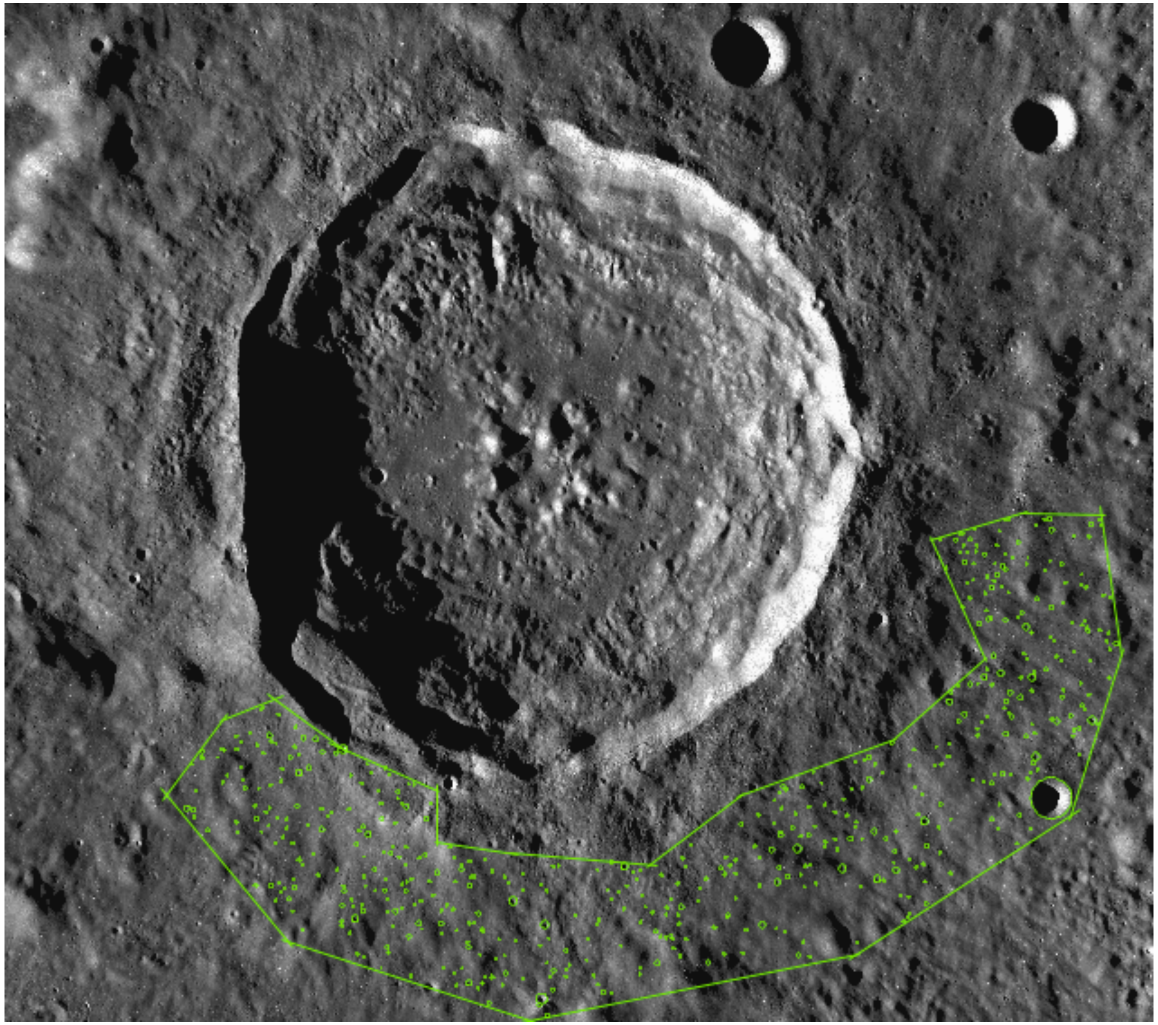

B

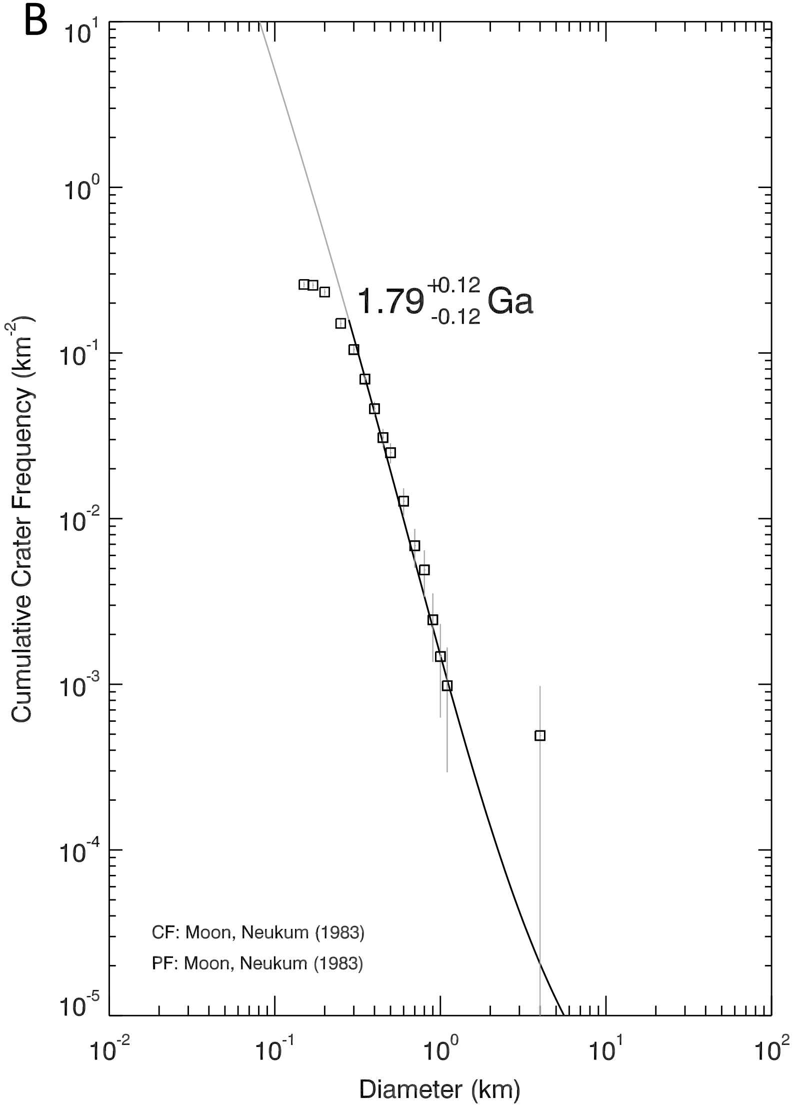

Supplementary Figure 19: The Terrain Camera image and the cumulative size-frequency distribution of Eudoxus

Pytheas

Diameter: 19.7 km

Number of craters: 87   Counting area: 159 km^2

N(1)=1.63$\times$10$^{-3}\pm$1.74$\times$10^{-4} km^{-2}, N(10)=4.15$\times$10$^{-6}\pm$4.40$\times$10^{-7} km^{-2}

A

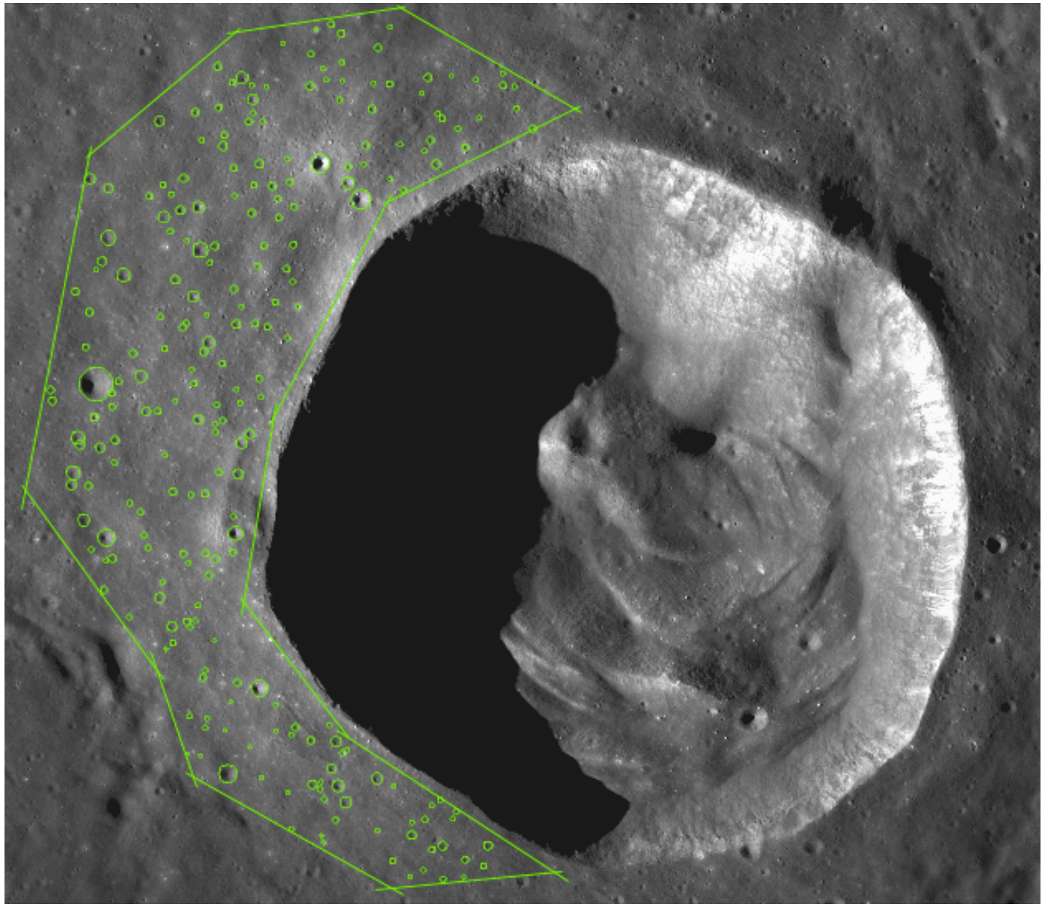

B

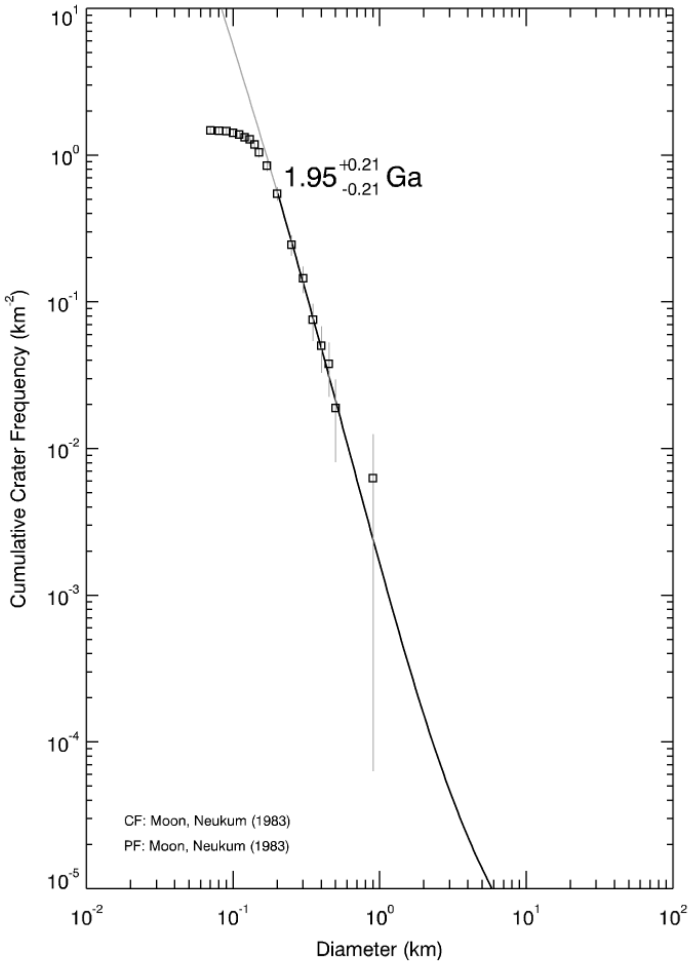

Supplementary Figure 20: The Terrain Camera image and the cumulative size-frequency distribution of Pytheas

Thebit A

Diameter: 20.5 km

Number of craters: 111 Counting area: 223 km^2

$N(1)=1.47\times10^{-3}\pm1.38\times10^{-4}$ km^{-2}, $N(10)=3.73\times10^{-6}\pm3.51\times10^{-7}$ km^{-2}

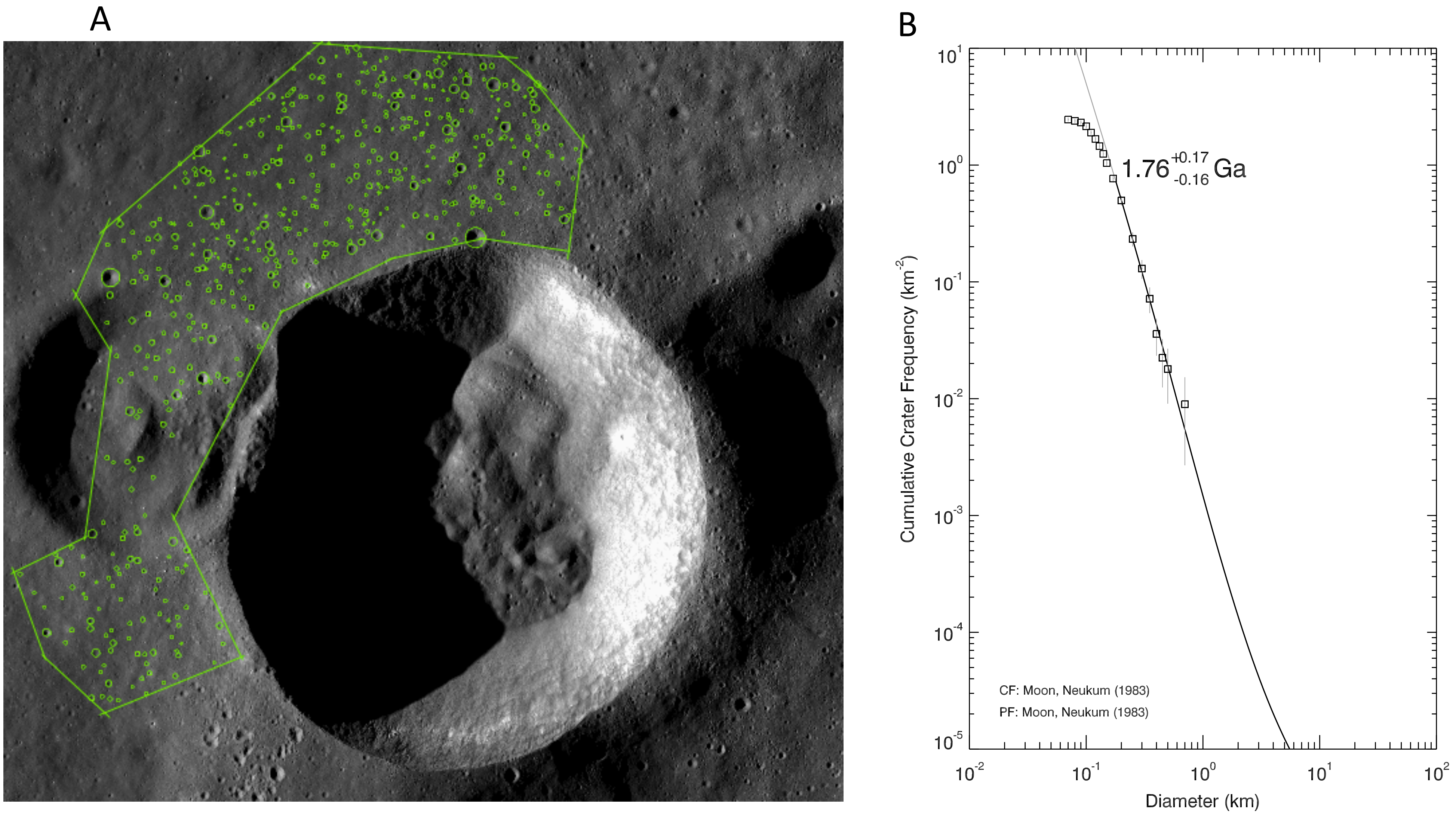

Supplementary Figure 21: The Terrain Camera image and the cumulative size-frequency distribution of Thebit A

Aristillus

Diameter: 54.5 km

Number of craters: Counting area: km^2

N(1)=$1.71\times10^{-3}\pm1.34\times10^{-4}$ km^{-2}, N(10)=$4.34\times10^{-6}\pm3.40\times10^{-7}$ km^{-2}

A

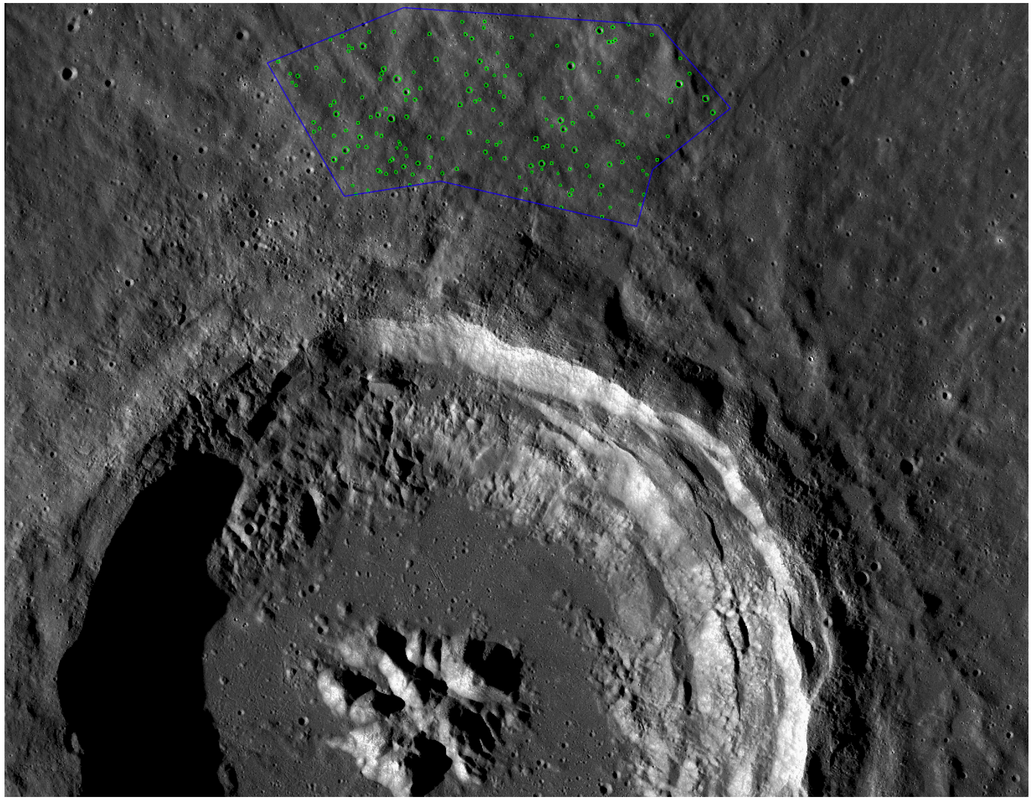

B

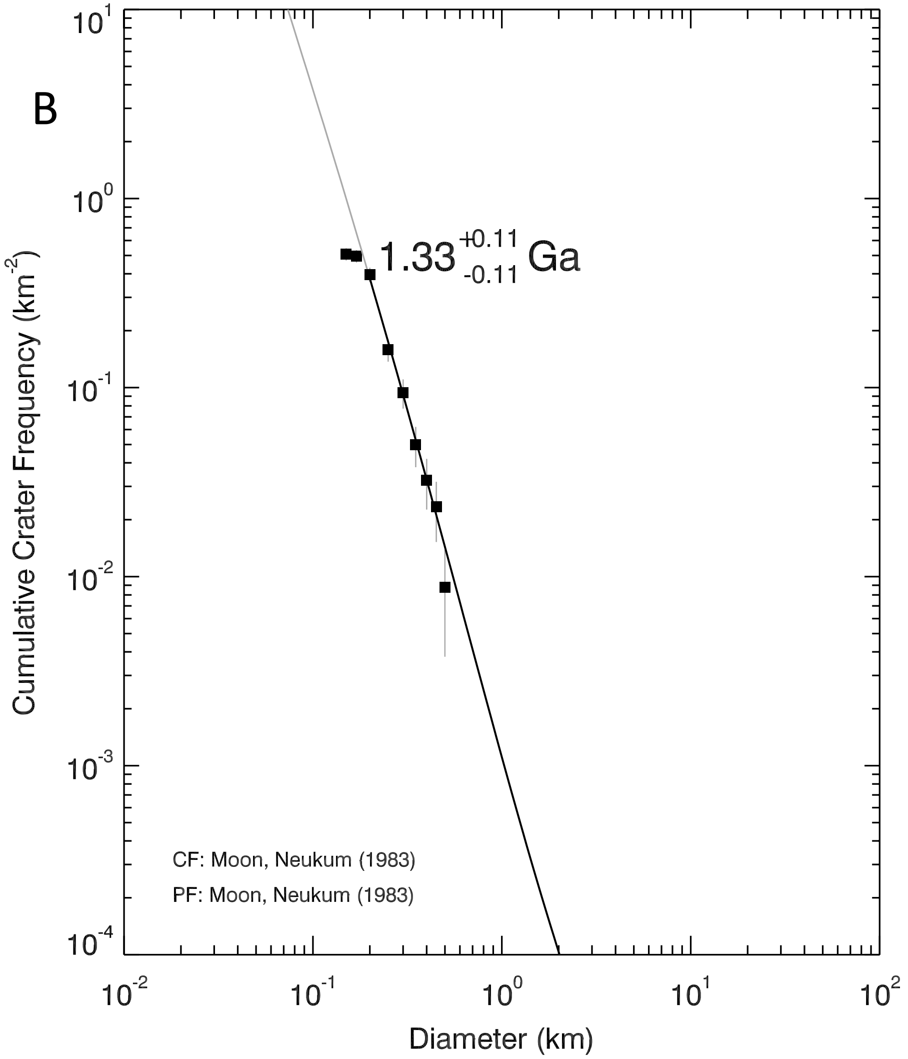

Supplementary Figure 22: The Terrain Camera image and the cumulative size-frequency distribution of Aristillus

Mosting

Diameter: 24.9 km

Number of craters: 172   Counting area: 590 km^2

N(1)=$1.84 \times 10^{-3} \pm 1.39 \times 10^{-4}$ km^{-2}, N(10)=$4.67 \times 10^{-6} \pm 3.53 \times 10^{-7}$ km^{-2}

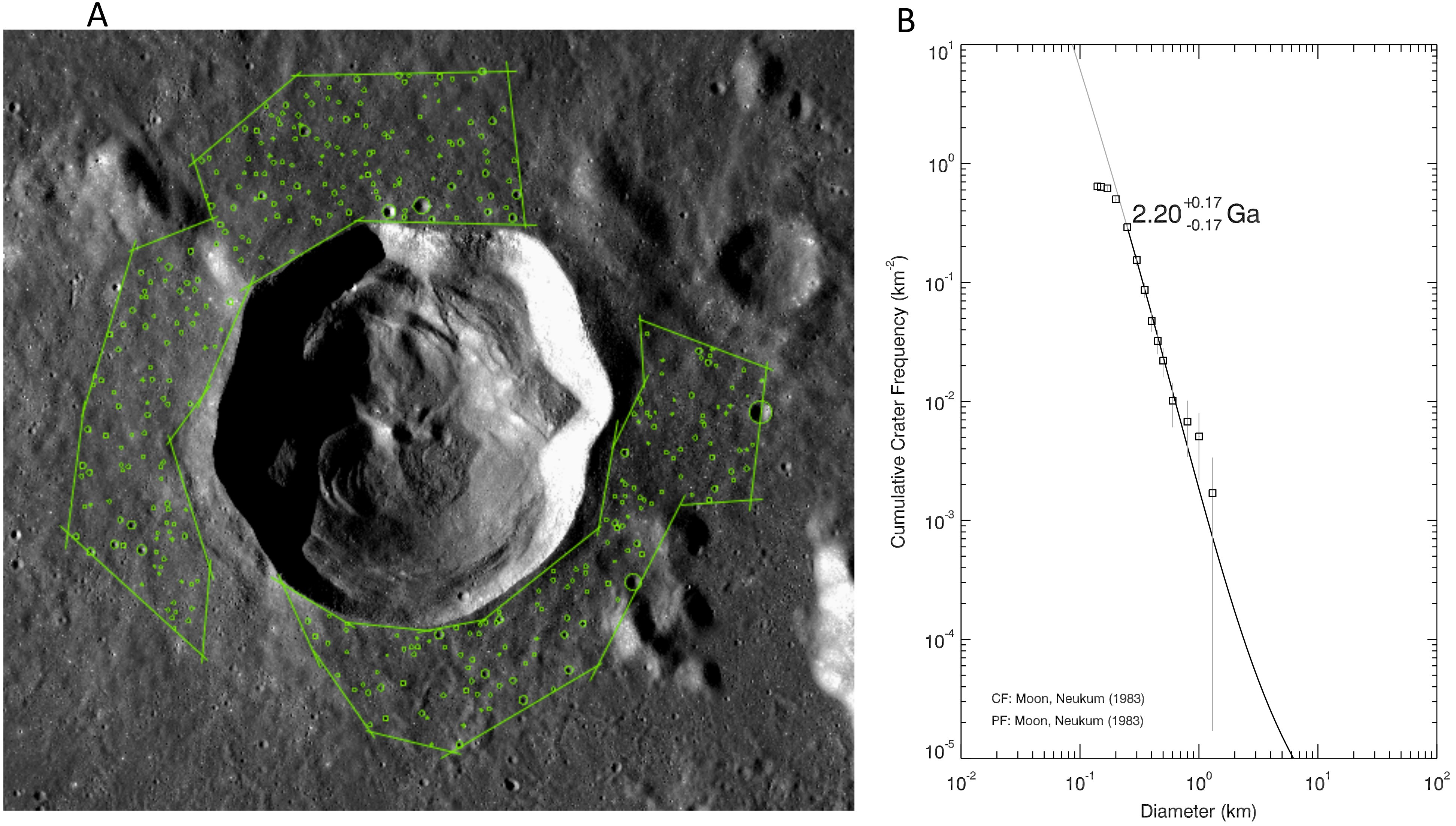

Supplementary Figure 23: The Terrain Camera image and the cumulative size-frequency distribution of Mosting

Briggs B

Diameter: 24.8 km

Number of craters: 111 Counting area: 383 km^2

N(1)=$1.96 \times 10^{-3} \pm 1.84 \times 10^{-4}$ km^{-2}, N(10)=$4.96 \times 10^{-6} \pm 4.66 \times 10^{-7}$ km^{-2}

A B

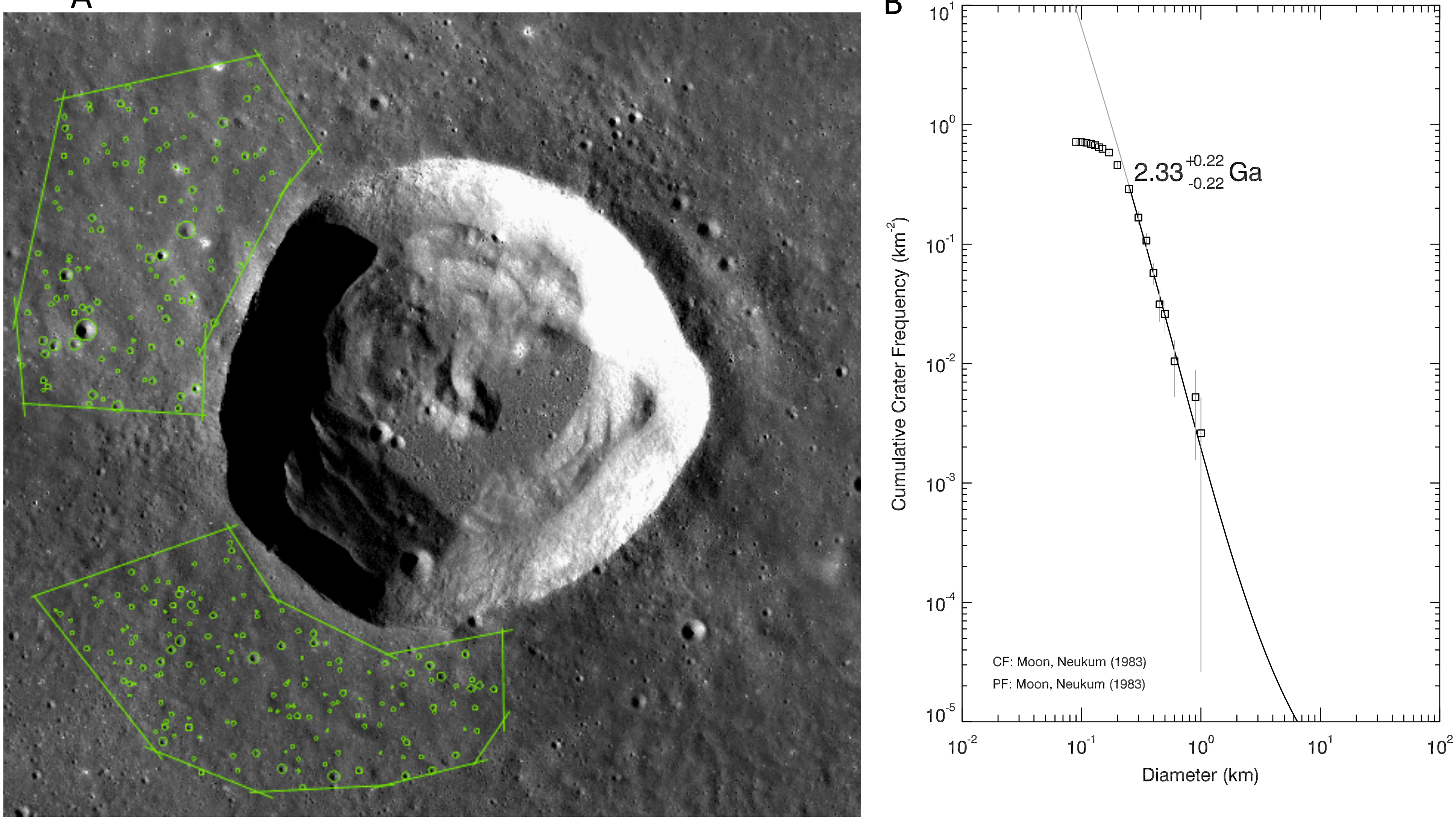

Supplementary Figure 24: The Terrain Camera image and the cumulative size-frequency distribution of Briggs B

Autolycus

Diameter: 38.9 km

Number of craters: 40 Counting area: 720 km^2

N(1)=$4.91 \times 10^{-3} \pm 7.69 \times 10^{-4}$ km^{-2}, N(10)=$1.25 \times 10^{-5} \pm 1.95 \times 10^{-6}$ km^{-2}

A

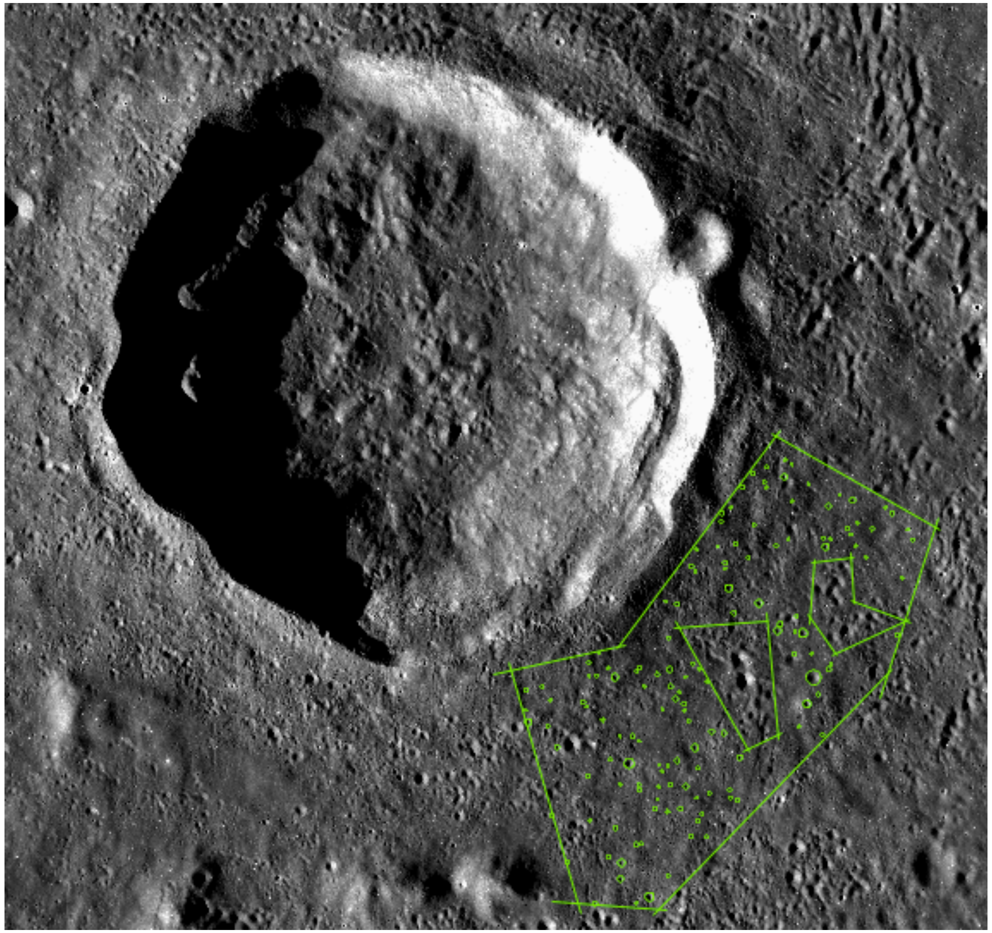

B

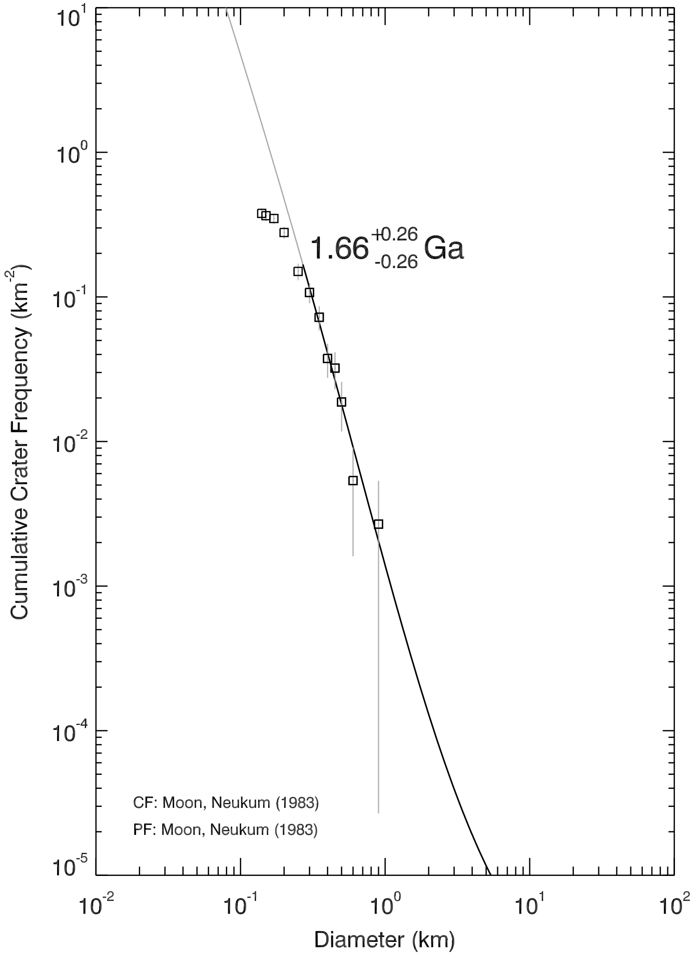

Supplementary Figure 25: The Terrain Camera image and the cumulative size-frequency distribution of Autolycus

Giordano Bruno

Diameter: 21.3 km

Number of craters: 116 Counting area: 362.1 km^2

$N(1)=5.60\times10^{-6}\pm5.15\times10^{-7}\ km^{-2}$, $N(10)=1.42\times10^{-8}\pm1.31\times10^{-9}\ km^{-2}$

A

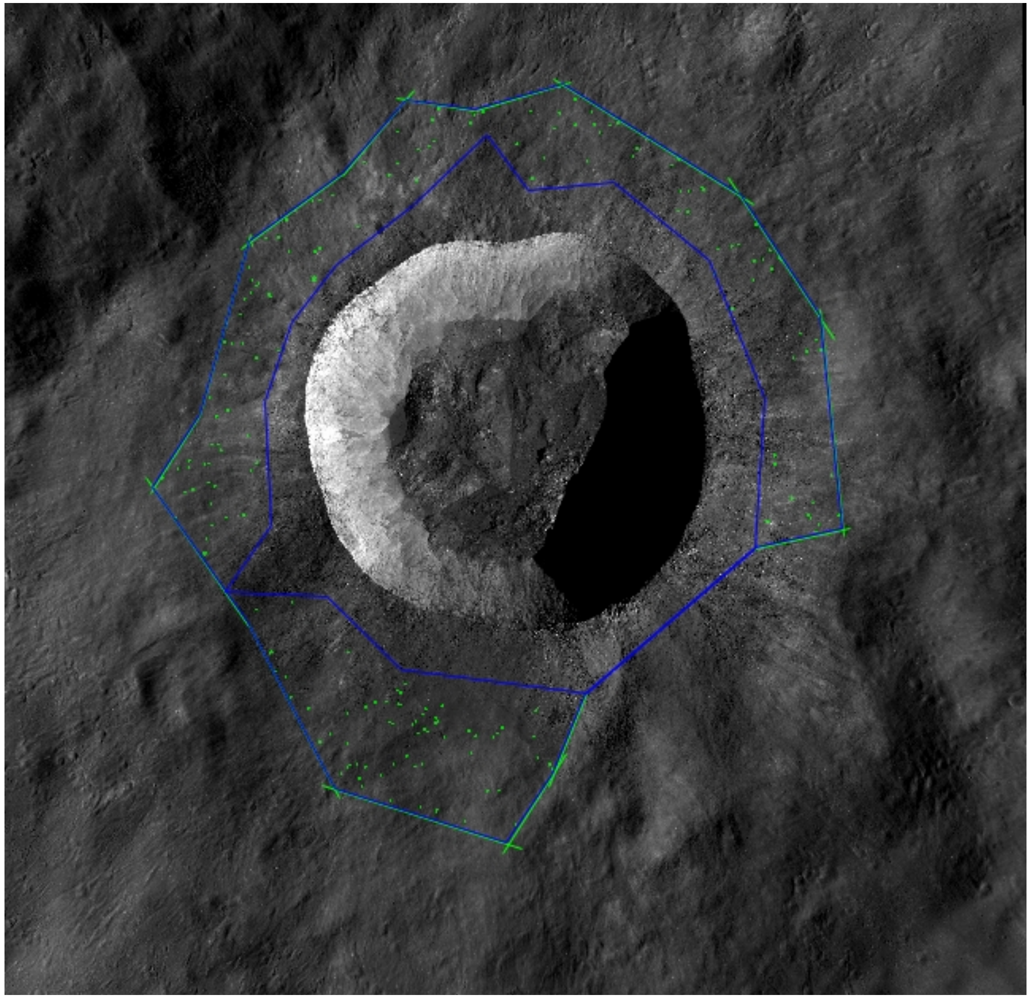

B

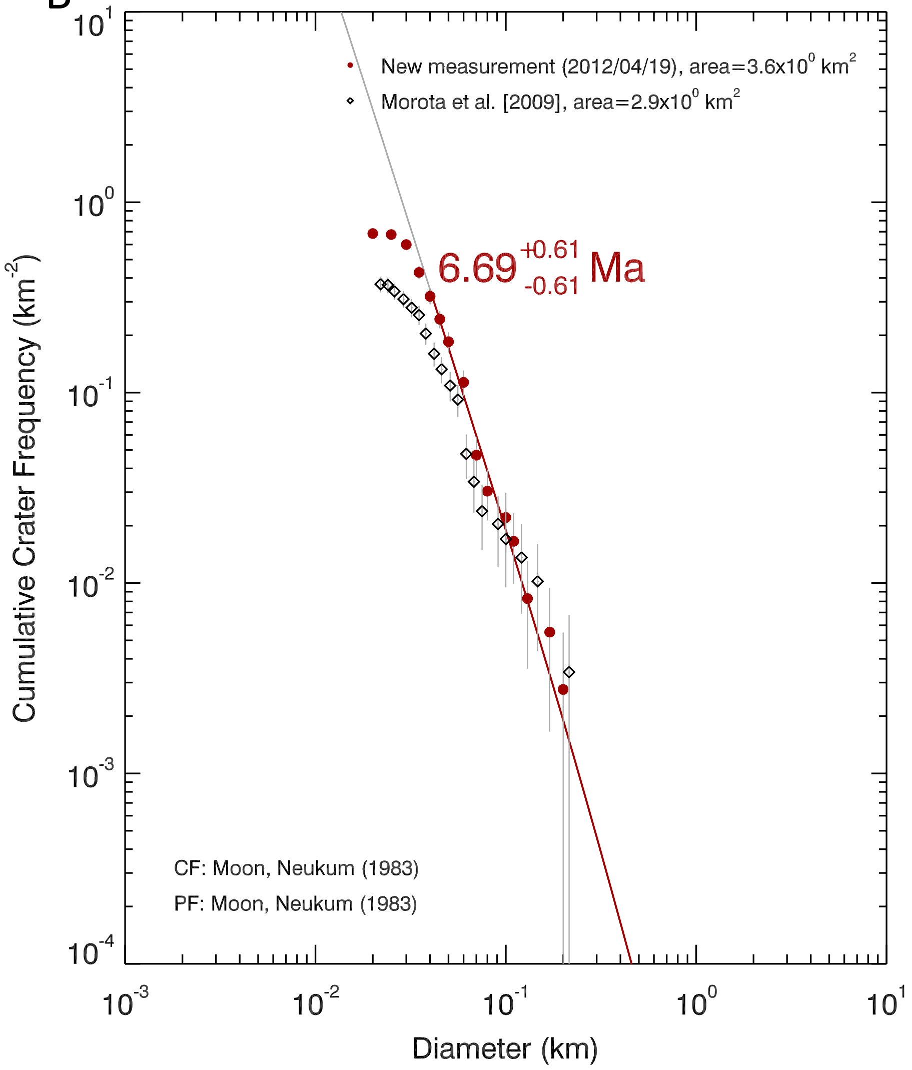

Supplementary Figure 26: The Terrain Camera image and the cumulative size-frequency distribution of Giordano Bruno

Moore F

Diameter: 23.7 km

Number of craters: 19 Counting area: 169.6 km^2

N(1)=$3.52\times10^{-5}\pm7.99\times10^{-6}$ km^{-2}, N(10)=$8.92\times10^{-8}\pm2.03\times10^{-9}$ km^{-2}

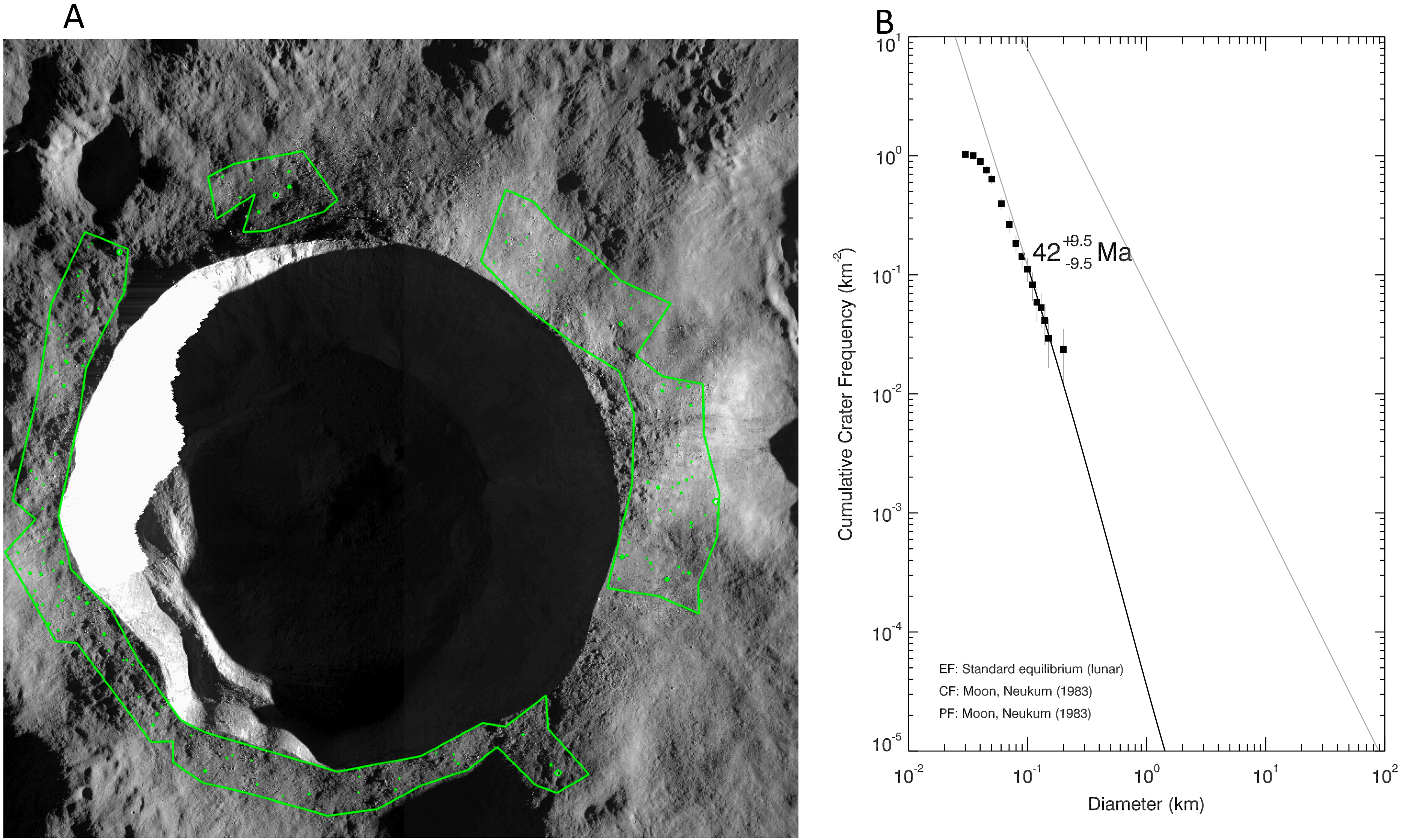

Supplementary Figure 27: The Terrain Camera image and the cumulative size-frequency distribution of Moore F

43S 143E (Ryder)

Diameter: 19.8 km

Number of craters: 43 Counting area: 155 km^2

N(1)=$5.73\times10^{-5}\pm8.56\times10^{-6}$ km^{-2}, N(10)=$1.45\times10^{-7}\pm2.19\times10^{-8}$ km^{-2}

A

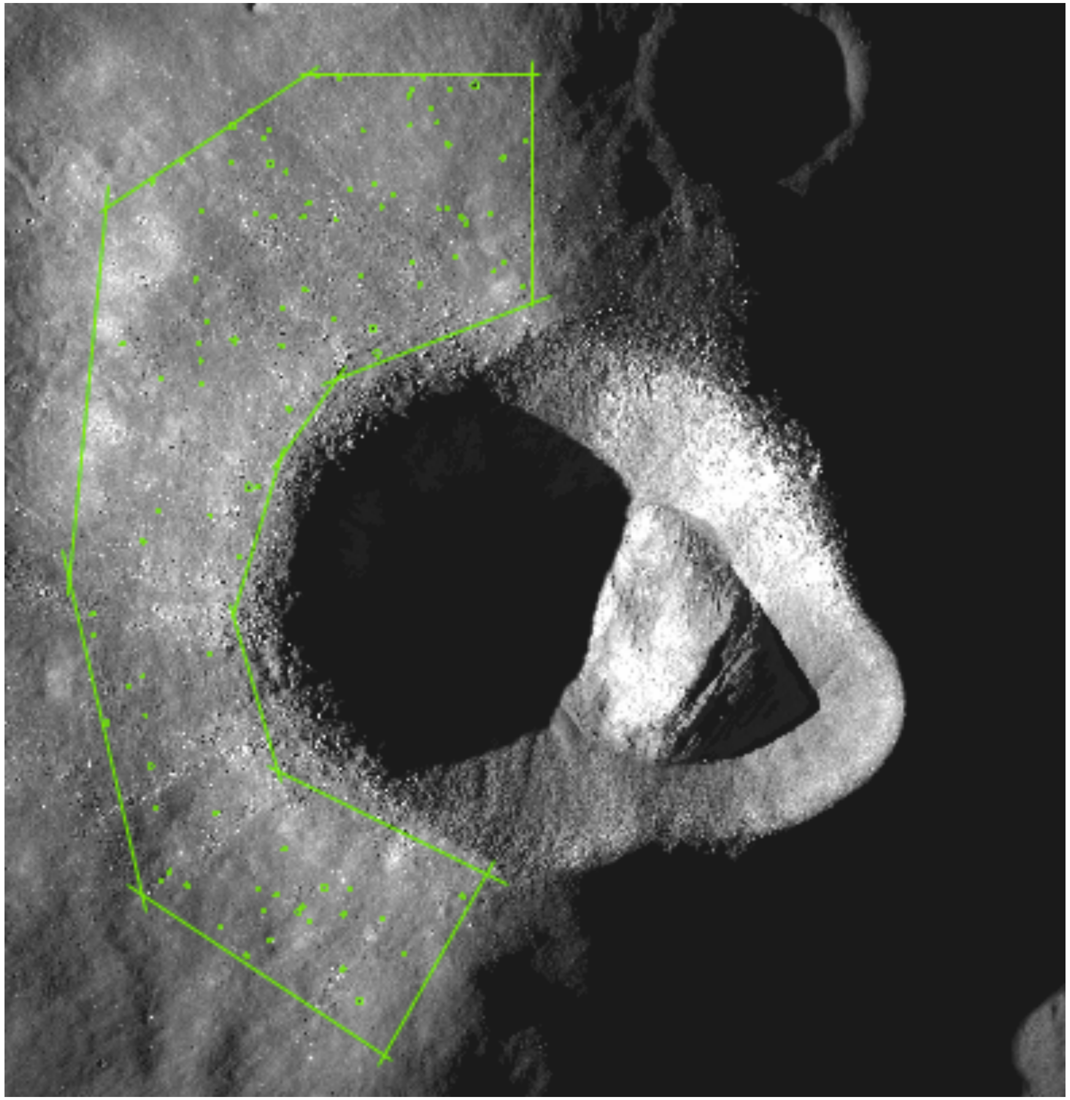

B

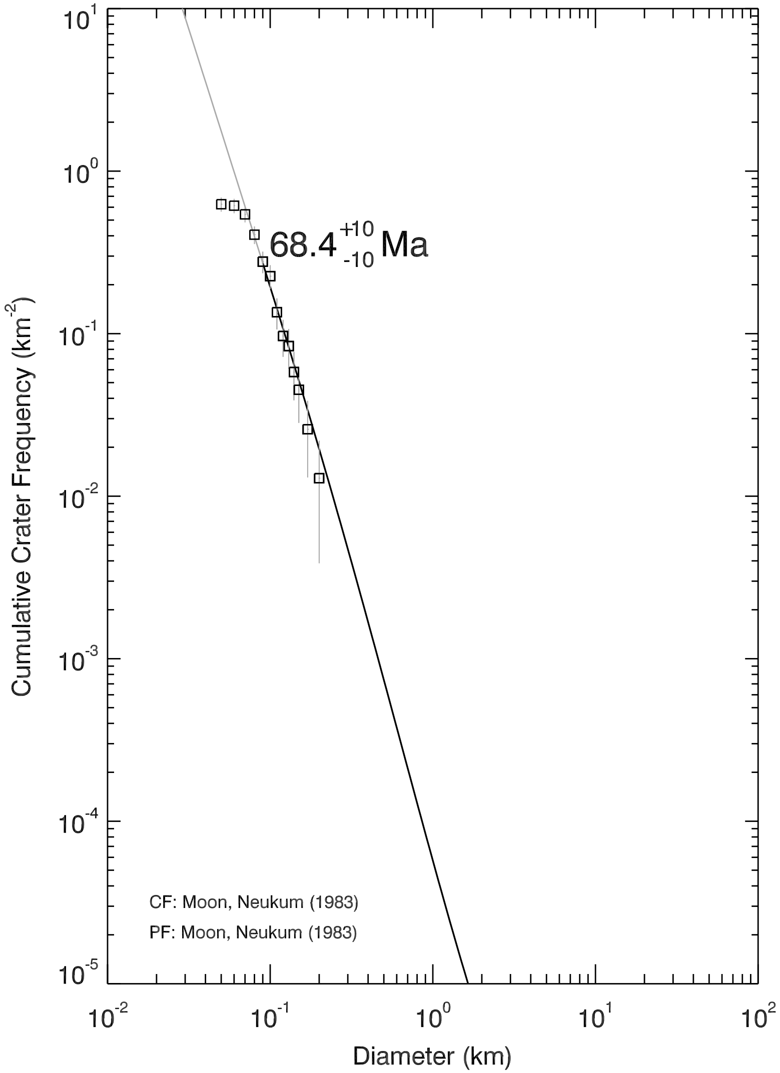

Supplementary Figure 28: The Terrain Camera image and the cumulative size-frequency distribution of 43S 143E (Ryder)

Lamor Q

Diameter: 25.5 km

Number of craters: 58 Counting area: 263 km^2

N(1)=6.83$\times$10$^{-5}\pm$8.88$\times$10^{-6} km^{-2}, N(10)=1.73$\times$10$^{-7}\pm$2.25$\times$10^{-8} km^{-2}

A

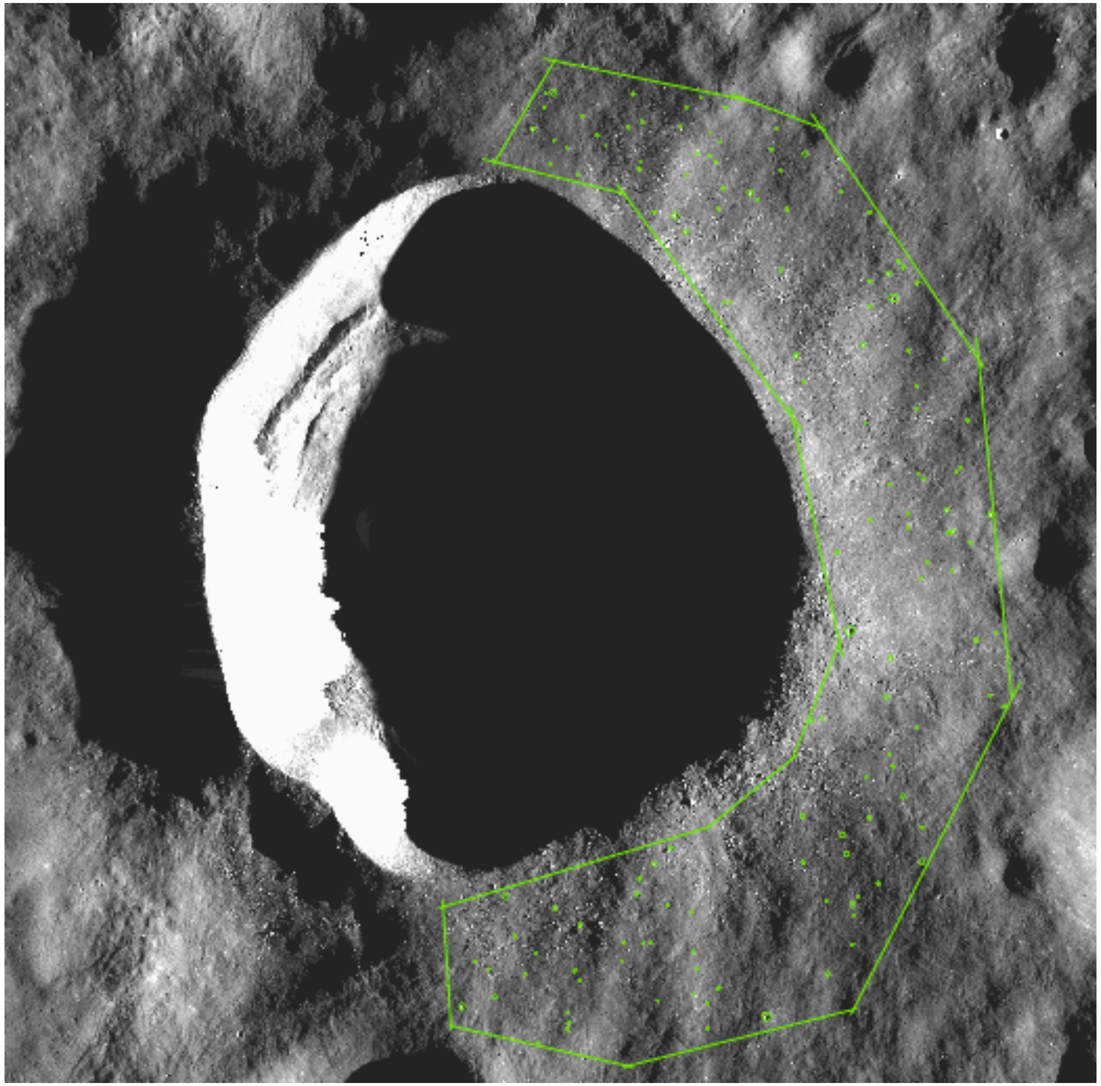

B

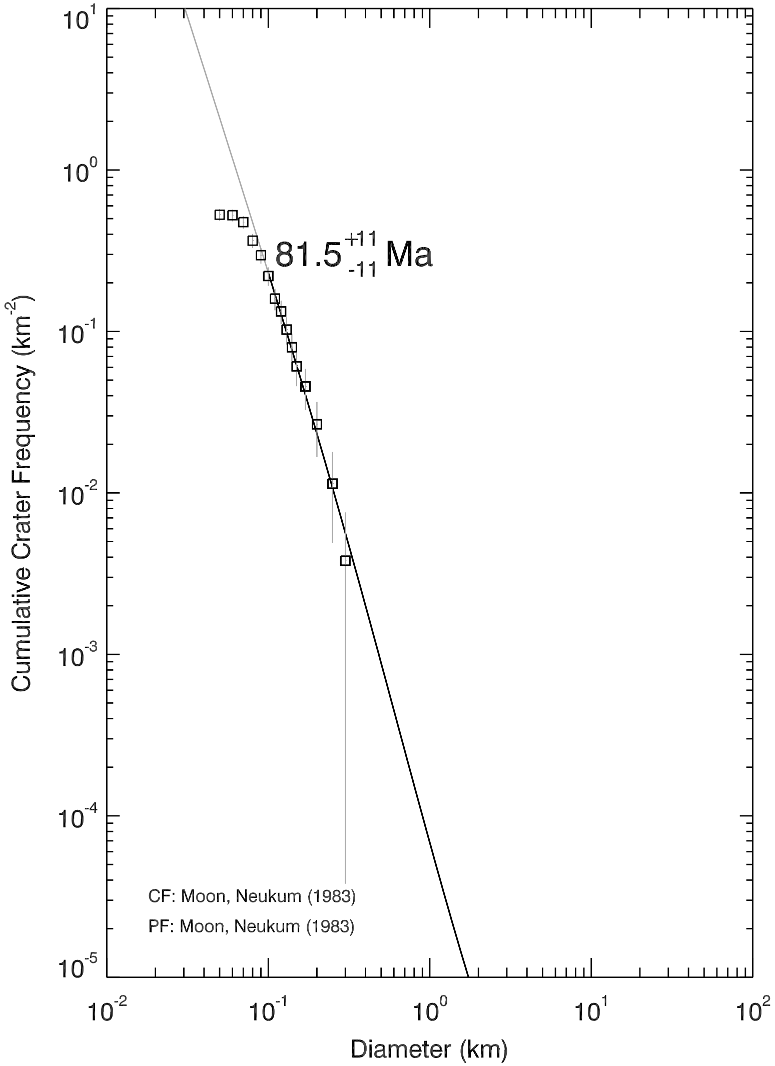

Supplementary Figure 29: The Terrain Camera image and the cumulative size-frequency distribution of Lamor Q

Joule T

Diameter: 38.1 km

Number of craters: 43 Counting area: 295 km^2

N(1)=$2.00\times10^{-3}\pm3.02\times10^{-4}$ km^{-2}, N(10)=$5.07\times10^{-6}\pm7.66\times10^{-7}$ km^{-2}

A

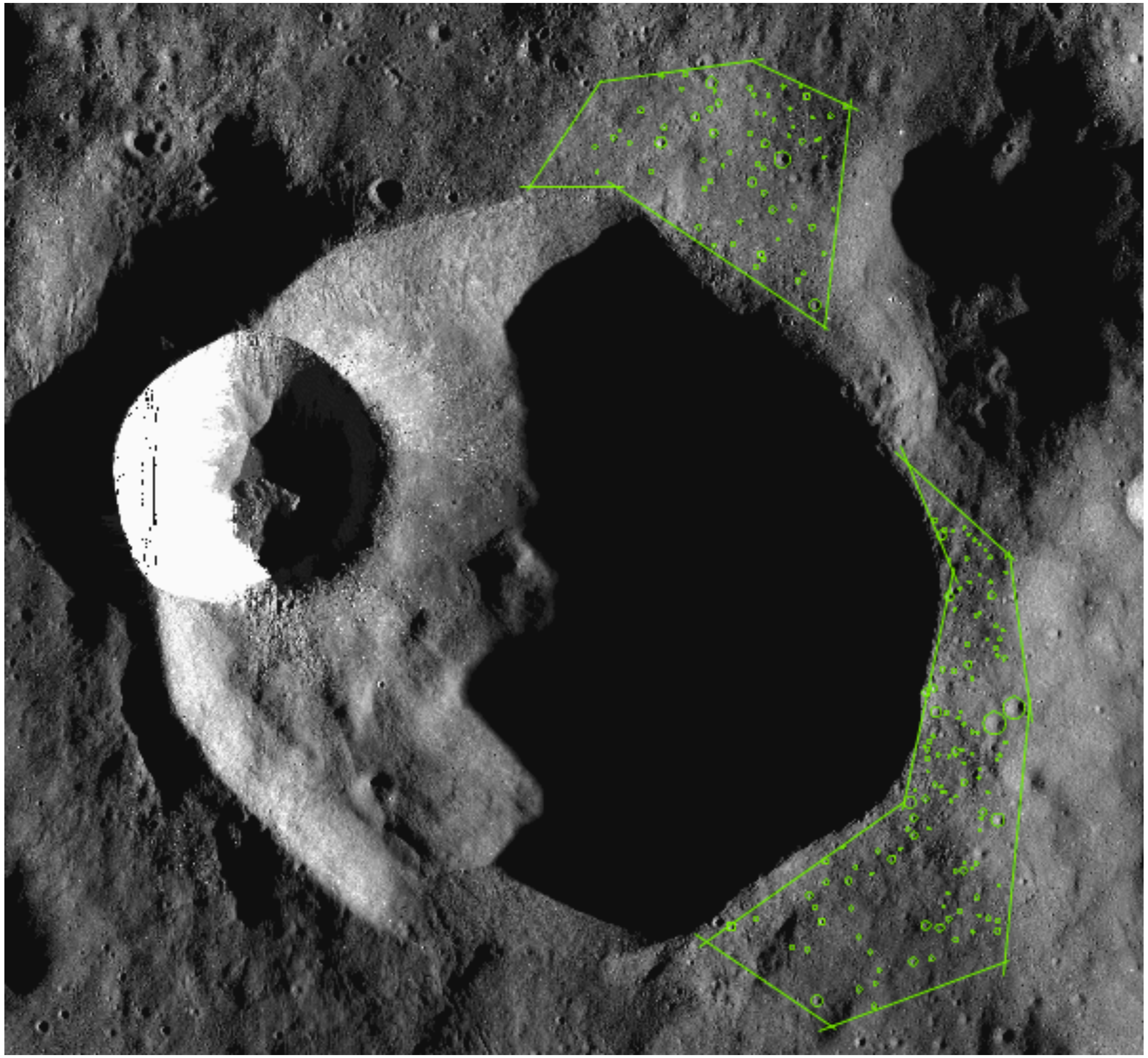

B

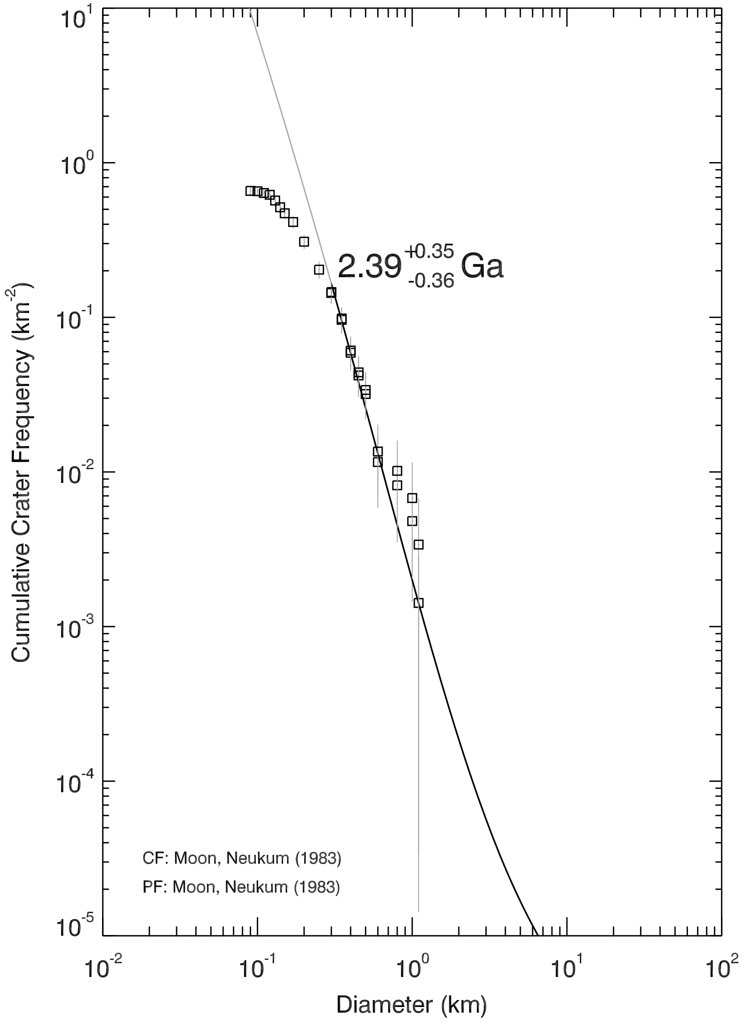

Supplementary Figure 30: The Terrain Camera image and the cumulative size-frequency distribution of Joule T

Necho

Diameter: 31.2 km

Number of craters: 382   Counting area: 195.4 km^2

N(1)=$6.86 \times 10^{-5} \pm 3.47 \times 10^{-6}$ km^{-2}, N(10)=$1.74 \times 10^{-7} \pm 0.88 \times 10^{-8}$ km^{-2}

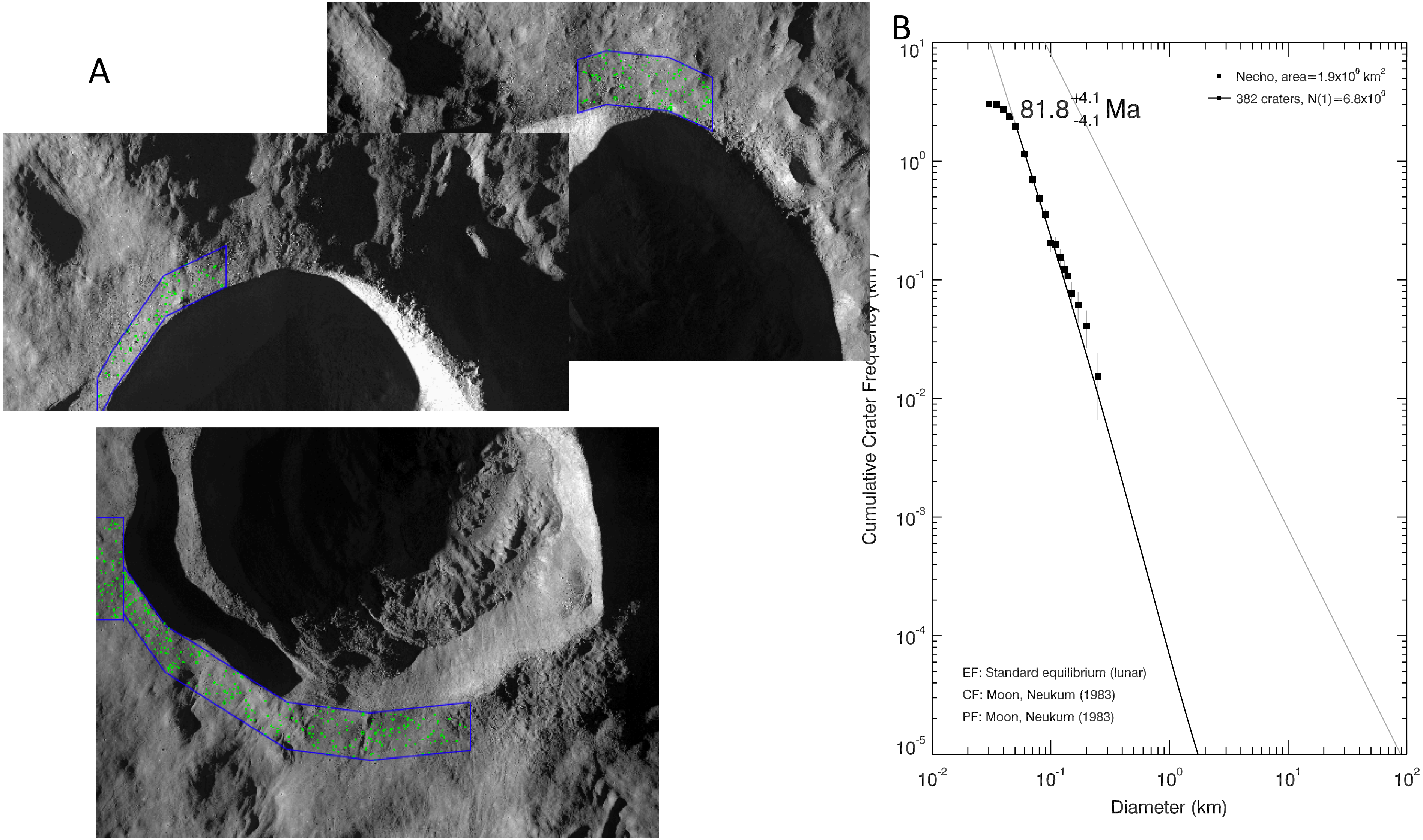

Supplementary Figure 31: The Terrain Camera images and the cumulative size-frequency distribution of Necho

Jackson

Diameter: 70.9 km

Number of craters: 55 Counting area: 466 km^2

N(1)=$1.21\times10^{-4}\pm1.61\times10^{-5}$ km^{-2}, N(10)=$3.07\times10^{-7}\pm4.09\times10^{-8}$ km^{-2}

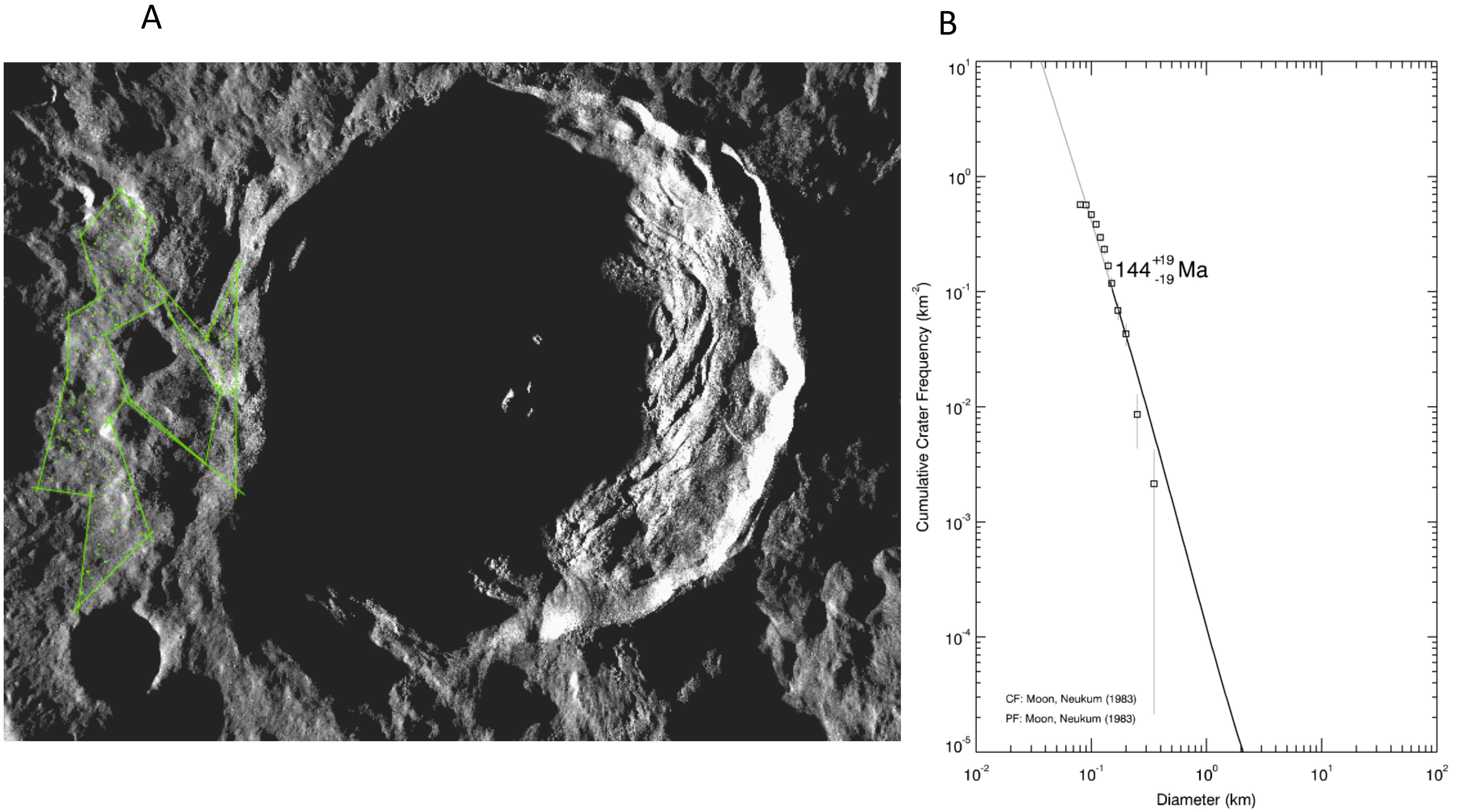

Supplementary Figure 32: The Terrain Camera image and the cumulative size-frequency distribution of Jackson

Ohm

Diameter: 63.9 km

Number of craters: 192 Counting area: 1468 km^2

$N(1)=2.05\times10^{-4}\pm1.46\times10^{-5}$ km^{-2}, $N(10)=5.20\times10^{-7}\pm3.71\times10^{-8}$ km^{-2}

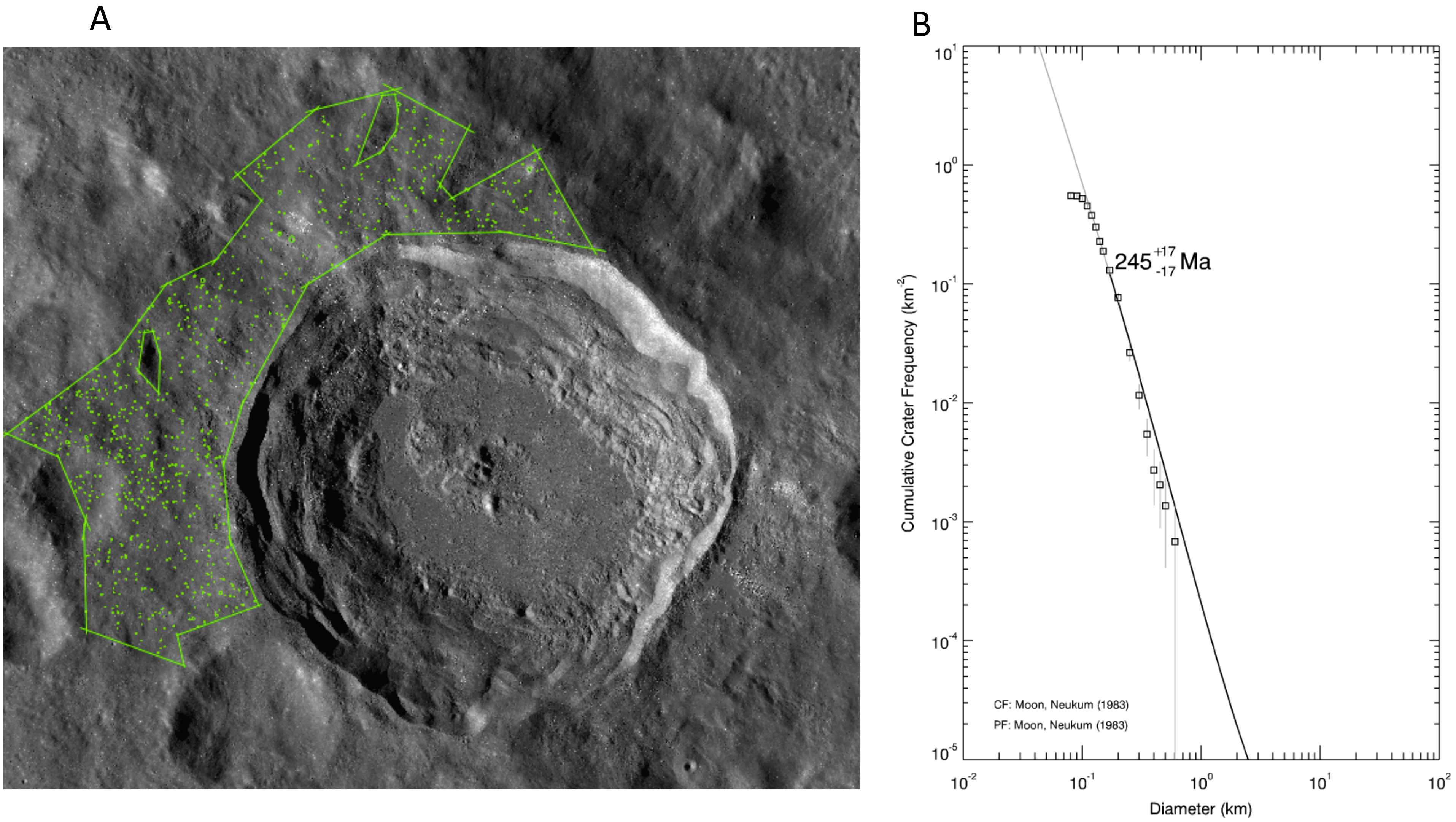

Supplementary Figure 33: The Terrain Camera image and the cumulative size-frequency distribution of Ohm

Van Newman F

Diameter: 32.4 km

Number of craters: 47   Counting area: 641 km^2

$N(1)=2.34\times10^{-4}\pm3.38\times10^{-5}\ km^{-2}$, $N(10)=5.94\times10^{-7}\pm8.57\times10^{-8}\ km^{-2}$

A

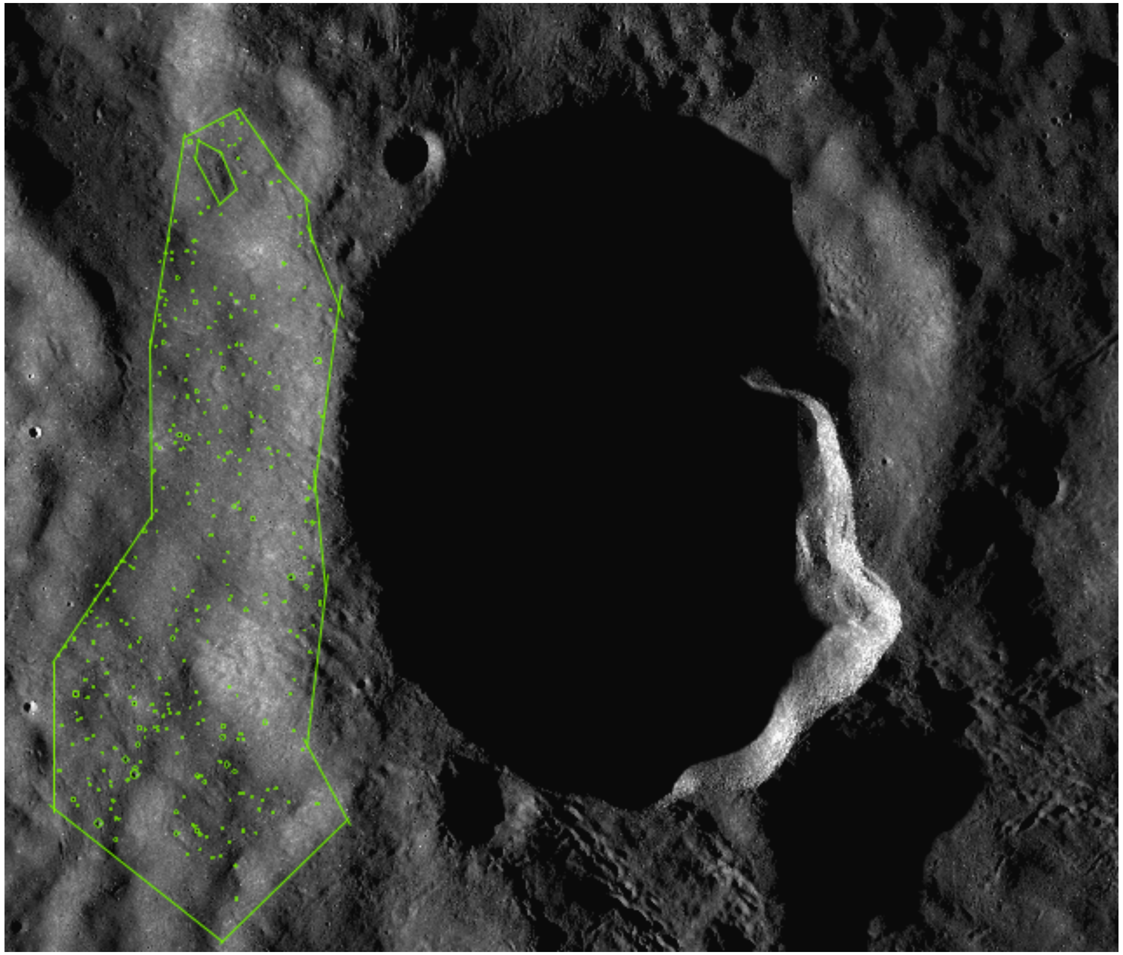

B

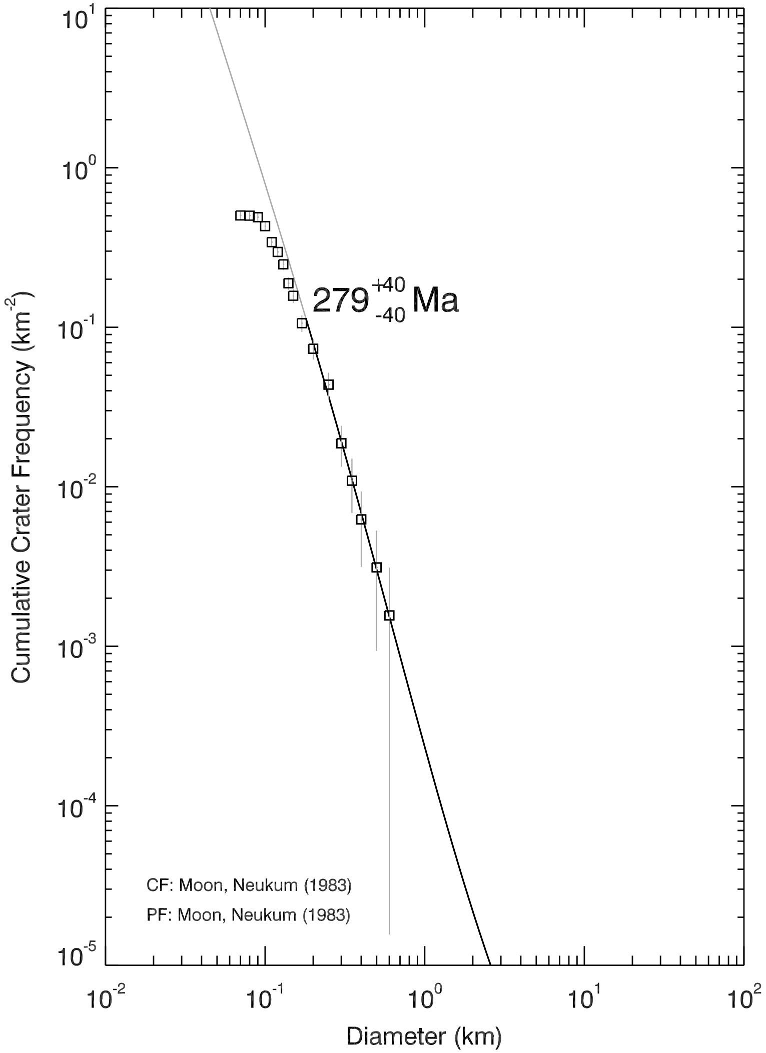

Supplementary Figure 34: The Terrain Camera image and the cumulative size-frequency distribution of Van Newman F

Crookes

Diameter: 48.8 km

Number of craters: 214 Counting area: 296 km^2

$N(1)=4.02\times10^{-4}\pm2.72\times10^{-5}$ km^{-2}, $N(10)=1.02\times10^{-6}\pm6.09\times10^{-8}$ km^{-2}

A B

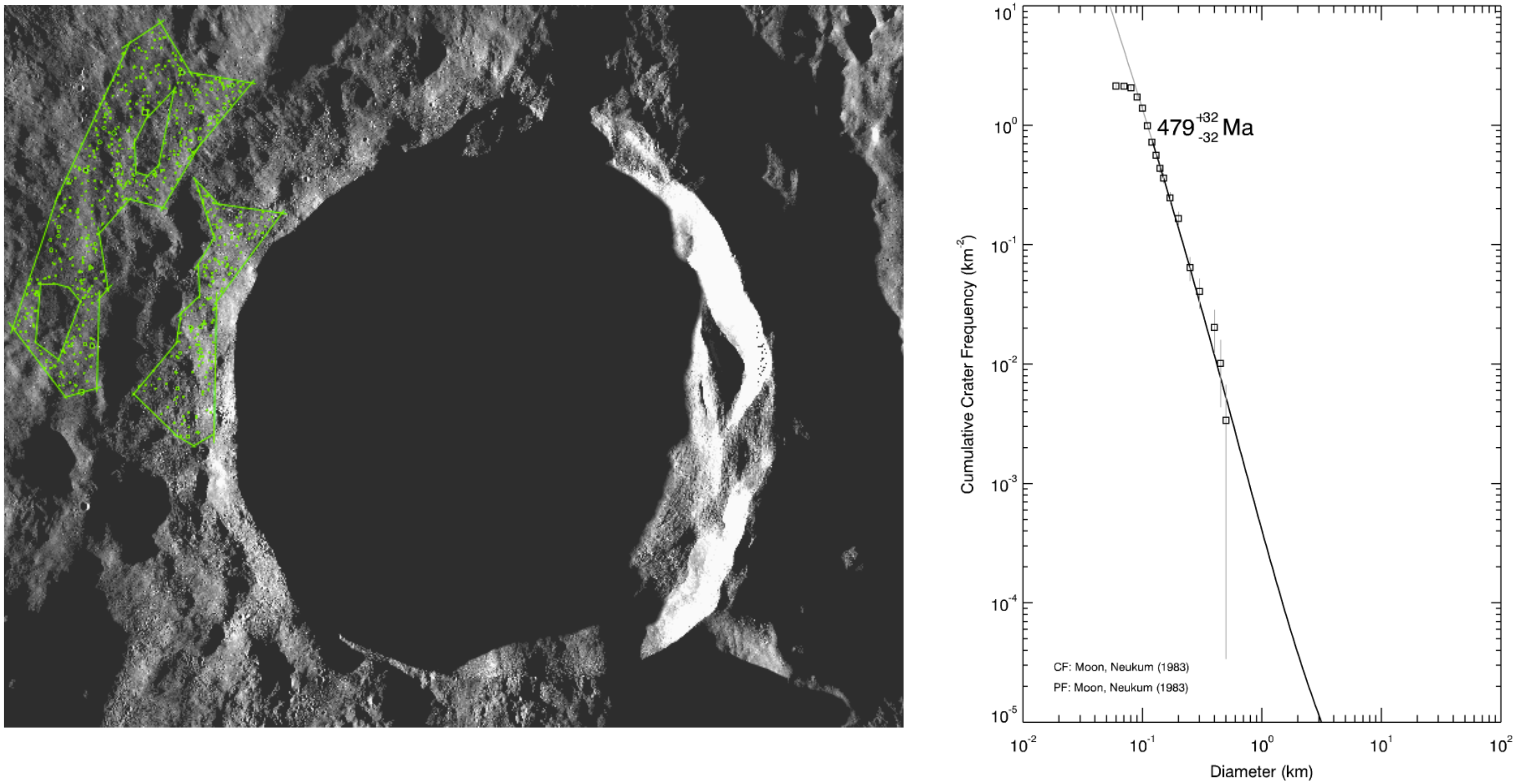

Supplementary Figure 35: The Terrain Camera image and the cumulative size-frequency distribution of Crookes

King

Diameter: 76.5 km

Number of craters: 198 Counting area: 1324 km^2

$N(1)=4.49\times10^{-4}\pm3.16\times10^{-5}$ km^{-2}, $N(10)=1.14\times10^{-6}\pm8.02\times10^{-8}$ km^{-2}

A B

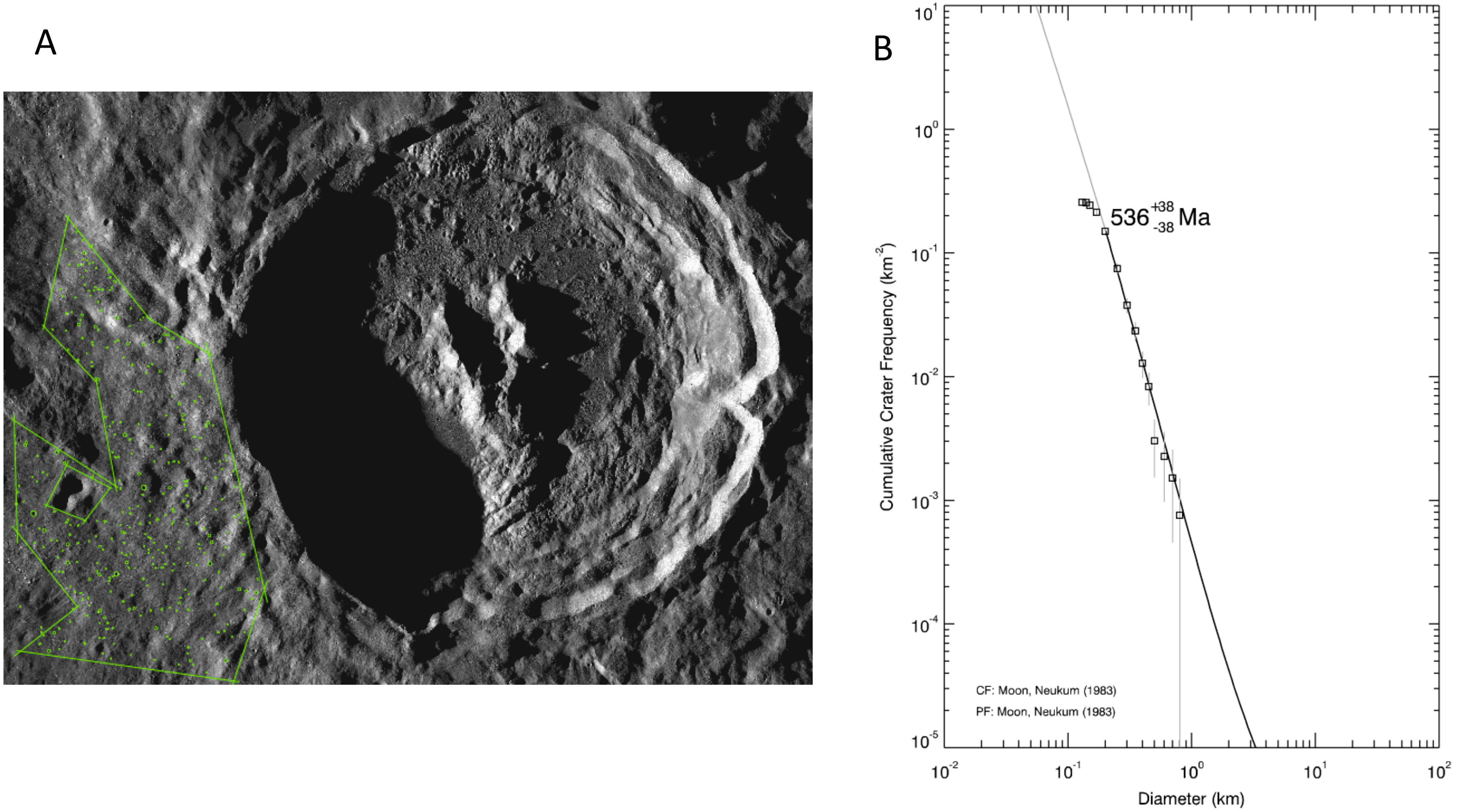

Supplementary Figure 36: The Terrain Camera image and the cumulative size-frequency distribution of King

Al-Khwarizmi K

Diameter: 22.5 km

Number of craters: 180 Counting area: 190.3 km^2

$N(1)=5.70\times10^{-4}\pm6.94\times10^{-5}$ km^{-2}, $N(10)=1.45\times10^{-6}\pm1.76\times10^{-7}$ km^{-2}

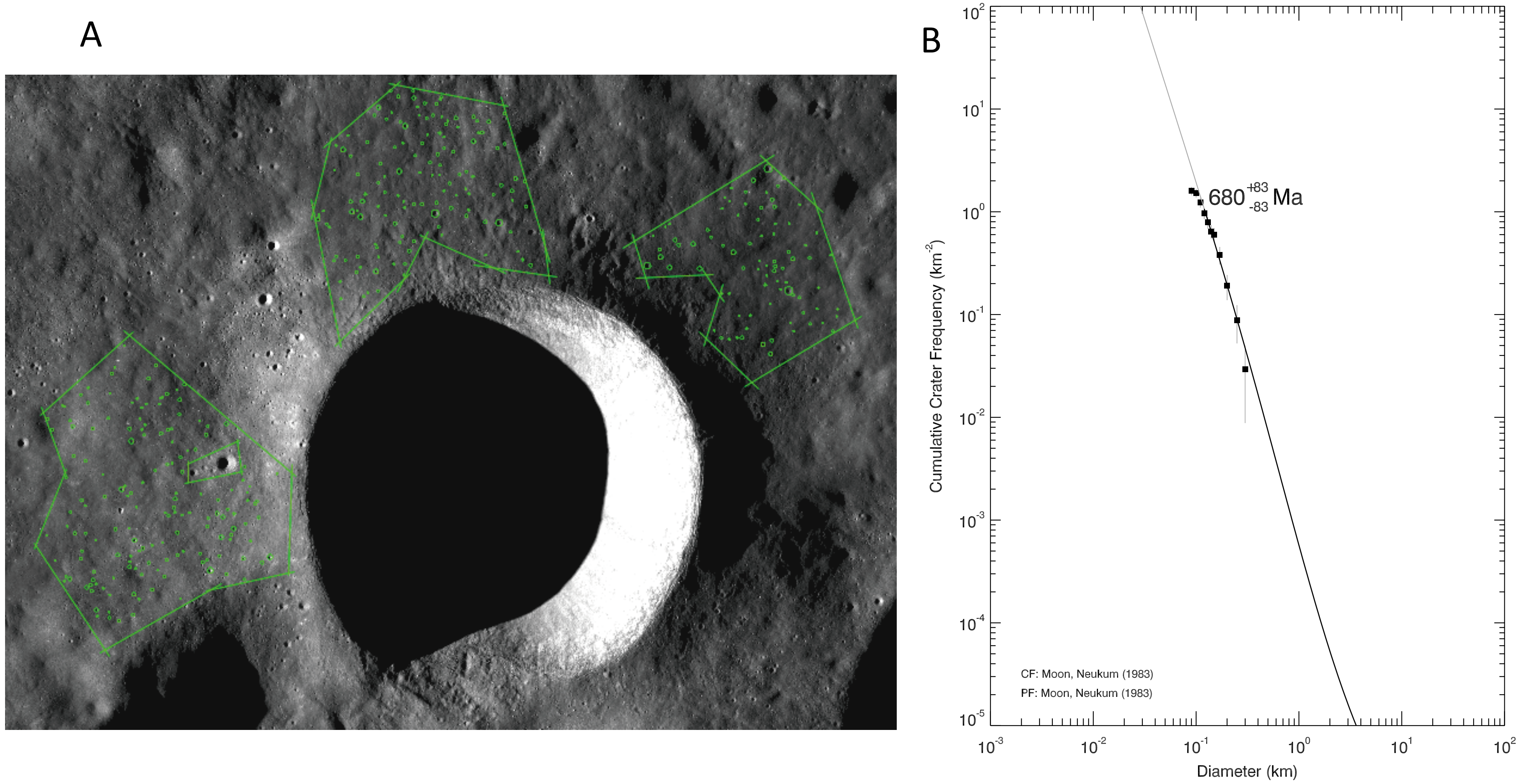

Supplementary Figure 37: The Terrain Camera image and the cumulative size-frequency distribution of Al-Khwarizmi K

Klute W

Diameter: 30.2 km

Number of craters: 90 Counting area: 163.9 km^2

N(1)=$6.44\times10^{-4}\pm6.72\times10^{-5}$ km^{-2}, N(10)=$1.63\times10^{-6}\pm1.70\times10^{-7}$ km^{-2}

A

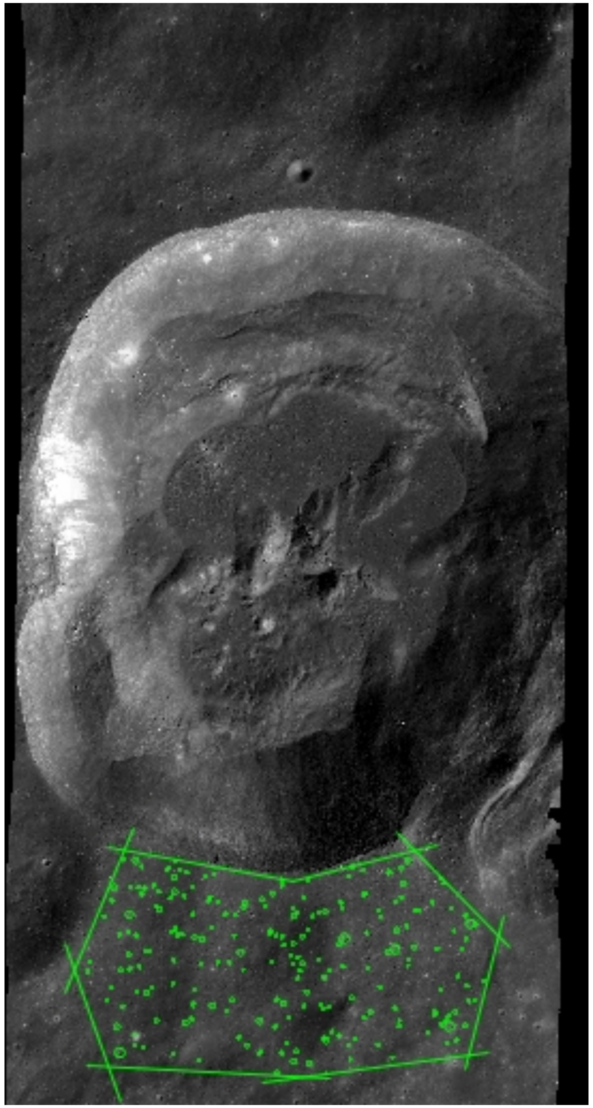

B

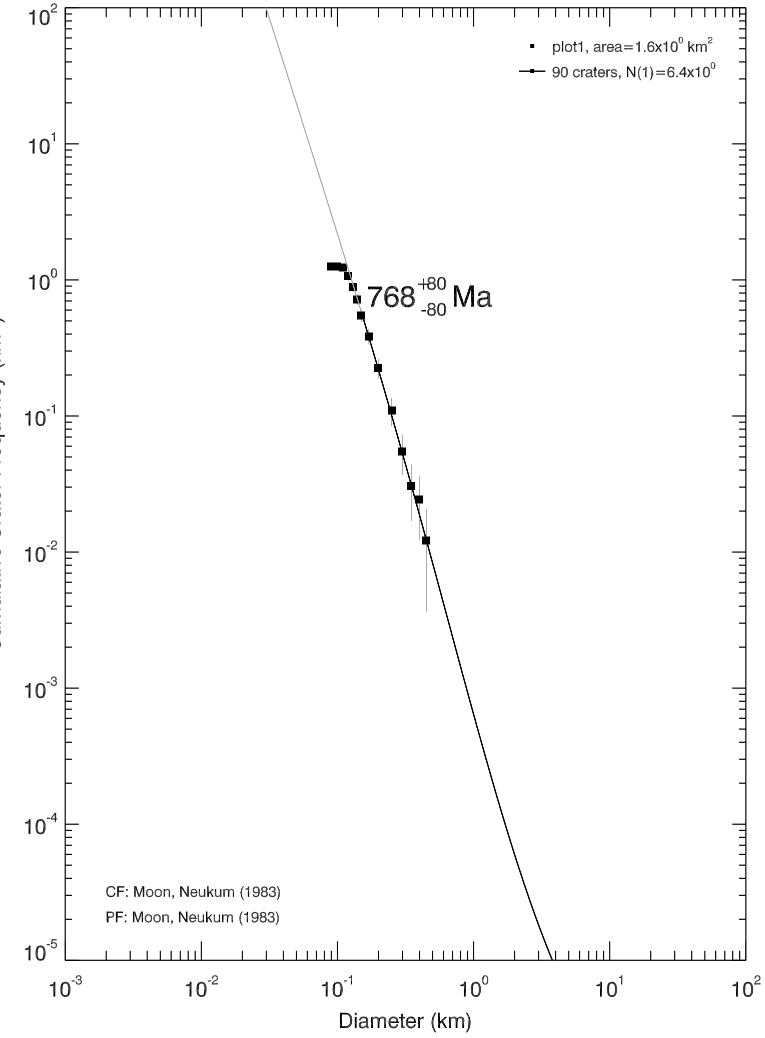

Supplementary Figure 38: The Terrain Camera image and the cumulative size-frequency distribution of Klute W

Saha E

Diameter: 29.4 km

Number of craters: 66 Counting area: 321 km^2

N(1)=$5.29 \times 10^{-4} \pm 6.45 \times 10^{-5}$ km^{-2}, N(10)=$1.34 \times 10^{-6} \pm 1.64 \times 10^{-7}$ km^{-2}

A

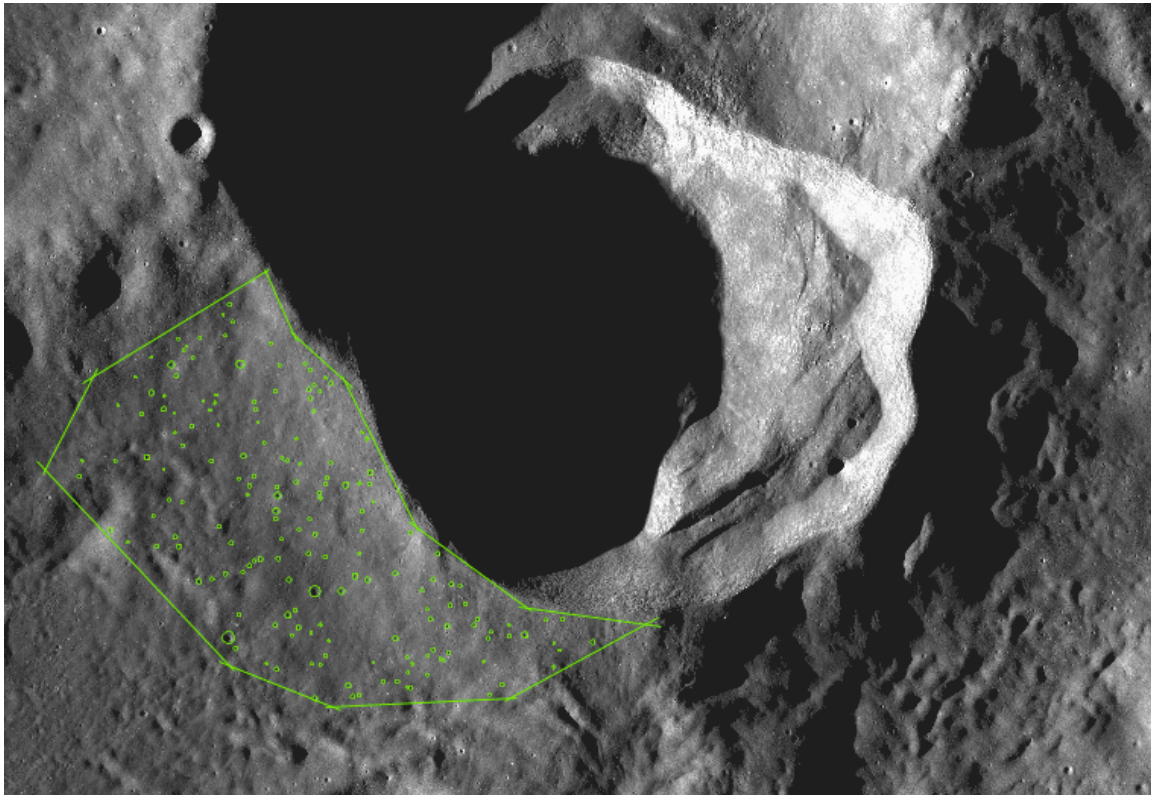

B

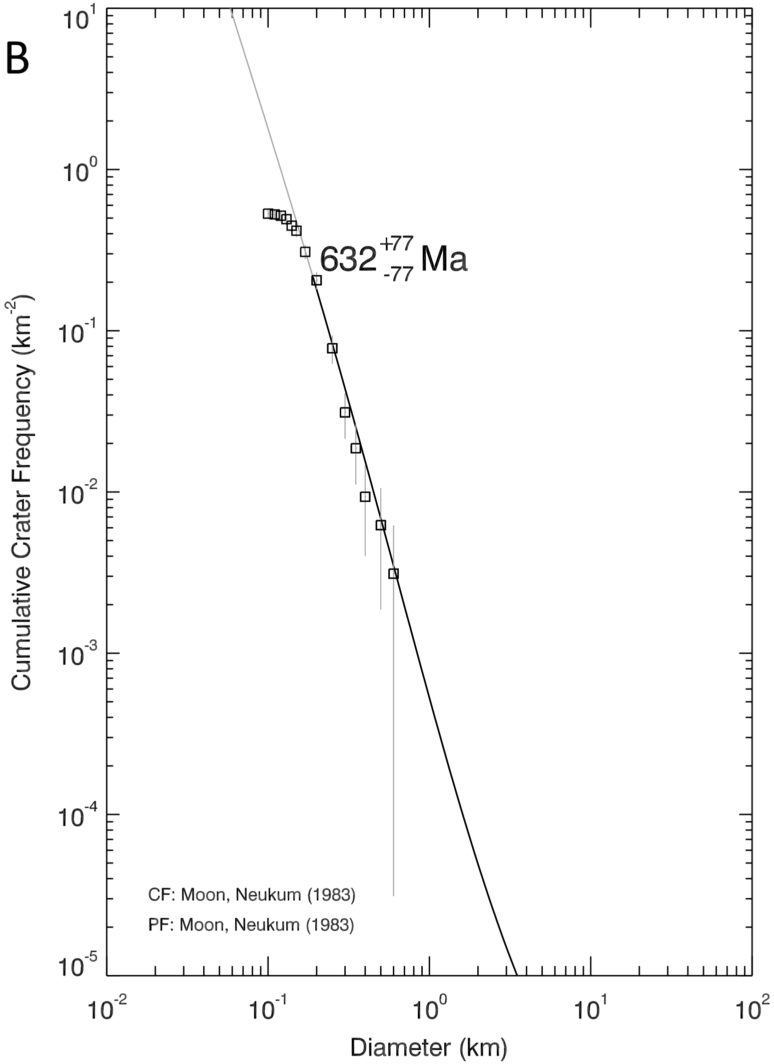

Supplementary Figure 39: The Terrain Camera image and the cumulative size-frequency distribution of Saha E

Lowell

Diameter: 65.6 km

Number of craters: 187 Counting area: 2319 km^2

N(1)=5.46$\times$10$^{-4}\pm$3.96$\times$10^{-5} km^{-2}, N(10)=1.39$\times$10$^{-6}\pm$1.00$\times$10^{-7} km^{-2}

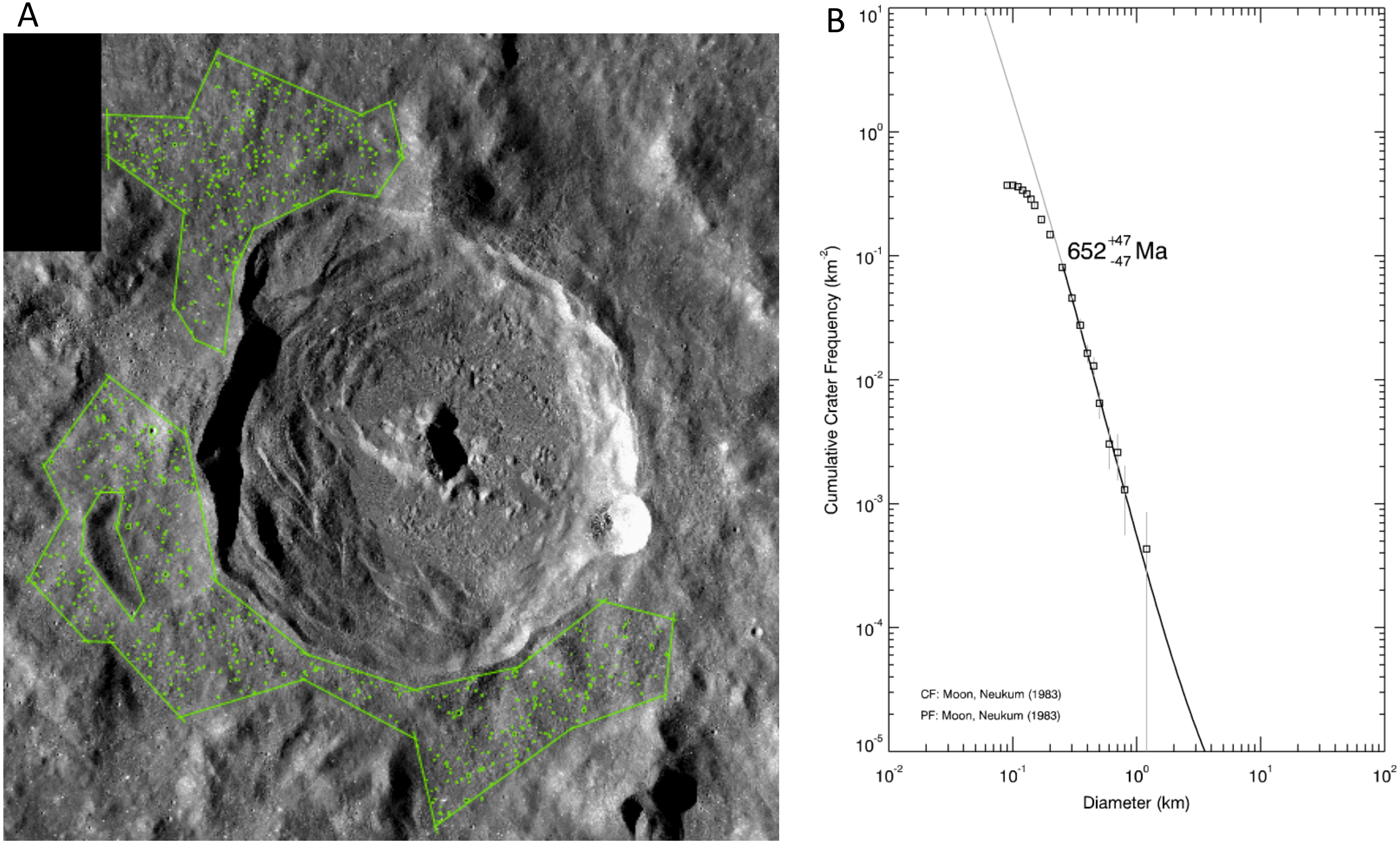

Supplementary Figure 40: The Terrain Camera image and the cumulative size-frequency distribution of Lowell

Stefan L

Diameter: 26.0 km

Number of craters: 133 Counting area: 660 km^2

N(1)=5.50$\times$10^{-4}$\pm$4.72$\times$10^{-5} km^{-2}, N(10)=1.40$\times$10^{-6}$\pm$1.20$\times$10^{-7} km^{-2}

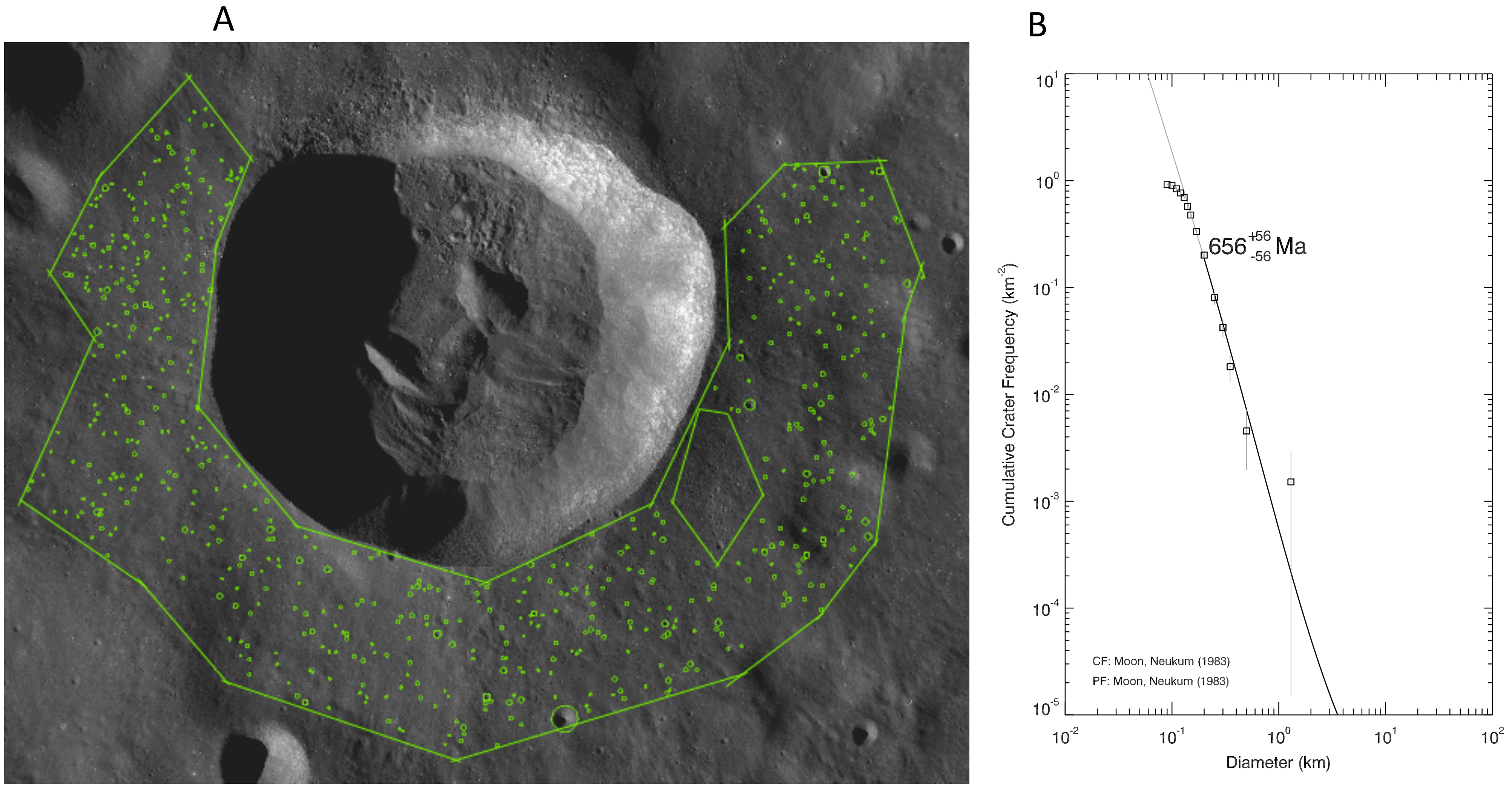

Supplementary Figure 41: The Terrain Camera image and the cumulative size-frequency distribution of Stefan L

Das

Diameter: 36.6 km

Number of craters: 104   Counting area: 549 km^2

$N(1)=5.52\times10^{-4}\pm5.36\times10^{-5}$ km^{-2}, $N(10)=1.40\times10^{-6}\pm1.36\times10^{-7}$ km^{-2}

A

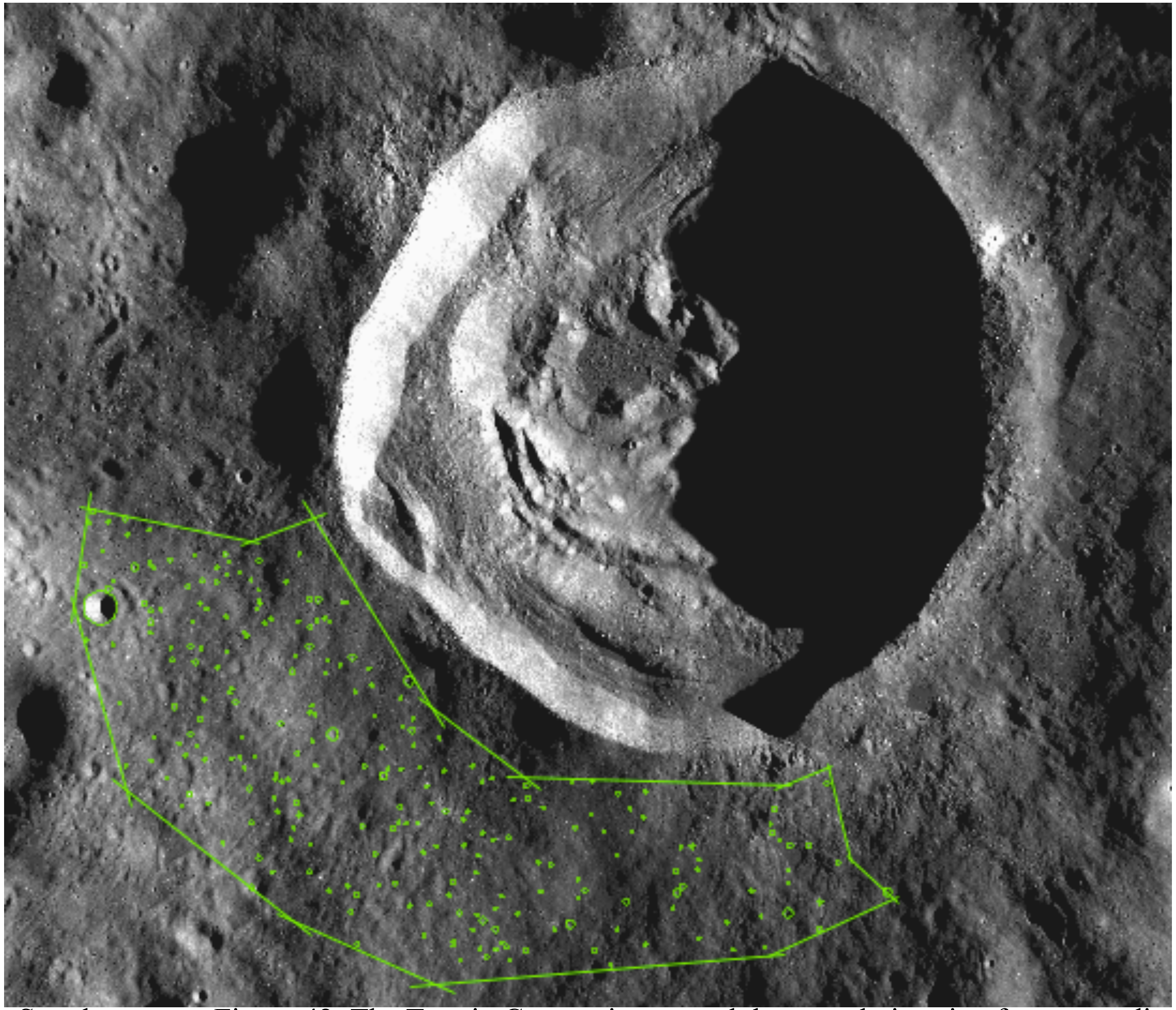

B

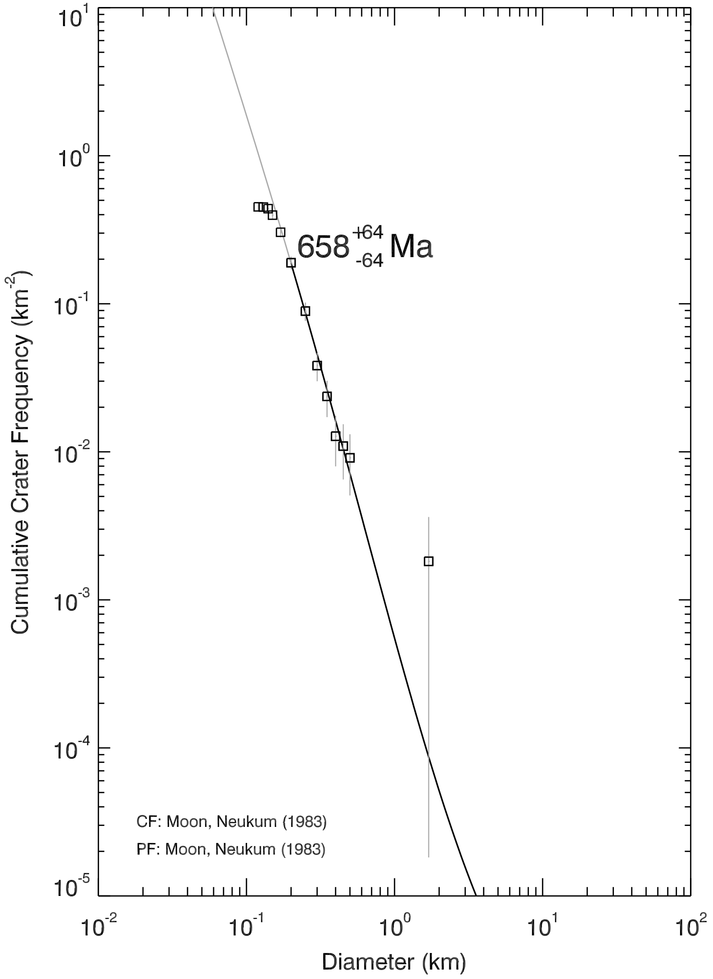

Supplementary Figure 42: The Terrain Camera image and the cumulative size-frequency distribution of Das

54S 161E

Diameter: 19.7 km

Number of craters: 64 Counting area: 99 km^2

$N(1)=6.26\times10^{-4}\pm7.75\times10^{-5}$ km^{-2}, $N(10)=1.59\times10^{-6}\pm1.96\times10^{-7}$ km^{-2}

A

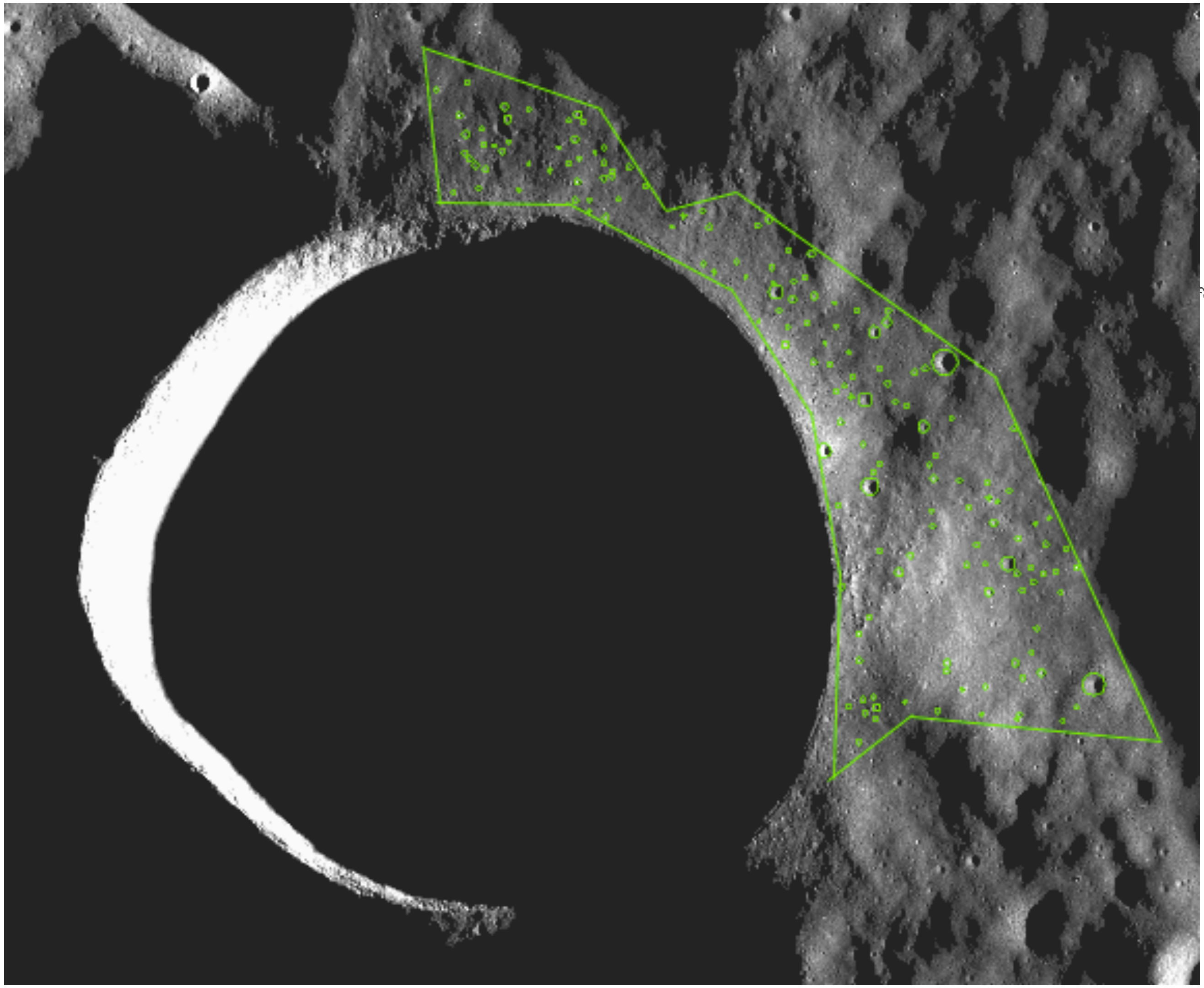

B

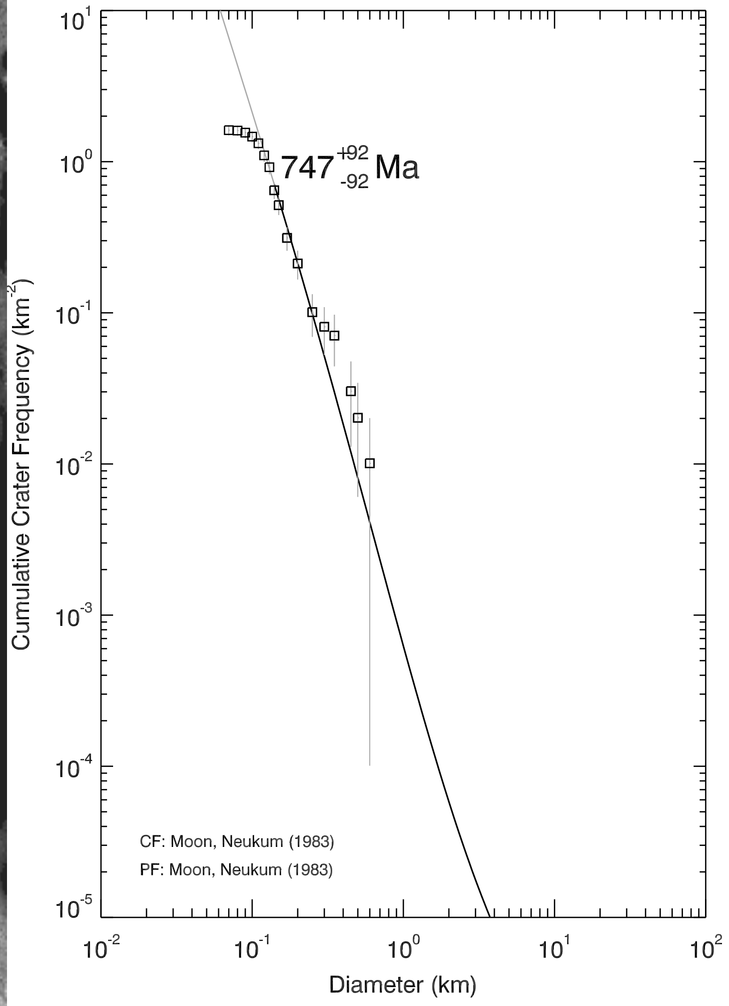

Supplementary Figure 43: The Terrain Camera image and the cumulative size-frequency distribution of 54S 161E

Zhukovsky Z

Diameter: 33.9 km

Number of craters: 46 Counting area: 153 km^2

N(1)=7.45$\times$10^{-4}$\pm$1.09$\times$10^{-4} km^{-2}, N(10)=1.89$\times$10^{-6}$\pm$2.76$\times$10^{-7} km^{-2}

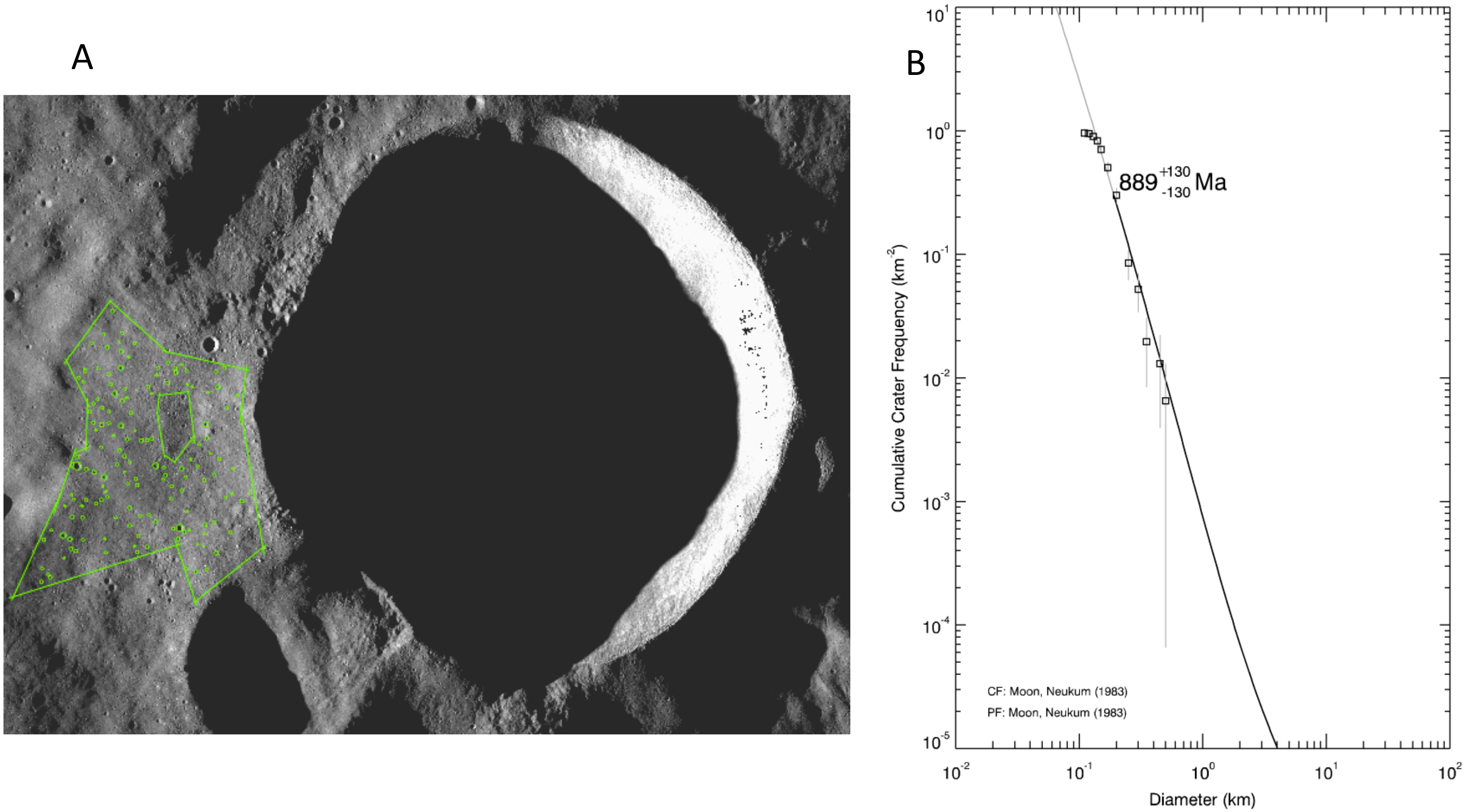

Supplementary Figure 44: The Terrain Camera image and the cumulative size-frequency distribution of Zhukovsky Z

Golitsyn

Diameter: 37.2 km

Number of craters: 76 Counting area: 62.3 km^2

N(1)=9.05×10^{-4}±1.04×10^{-4} km^{-2}, N(10)=2.30×10^{-6}±2.61×10^{-7} km^{-2}

A

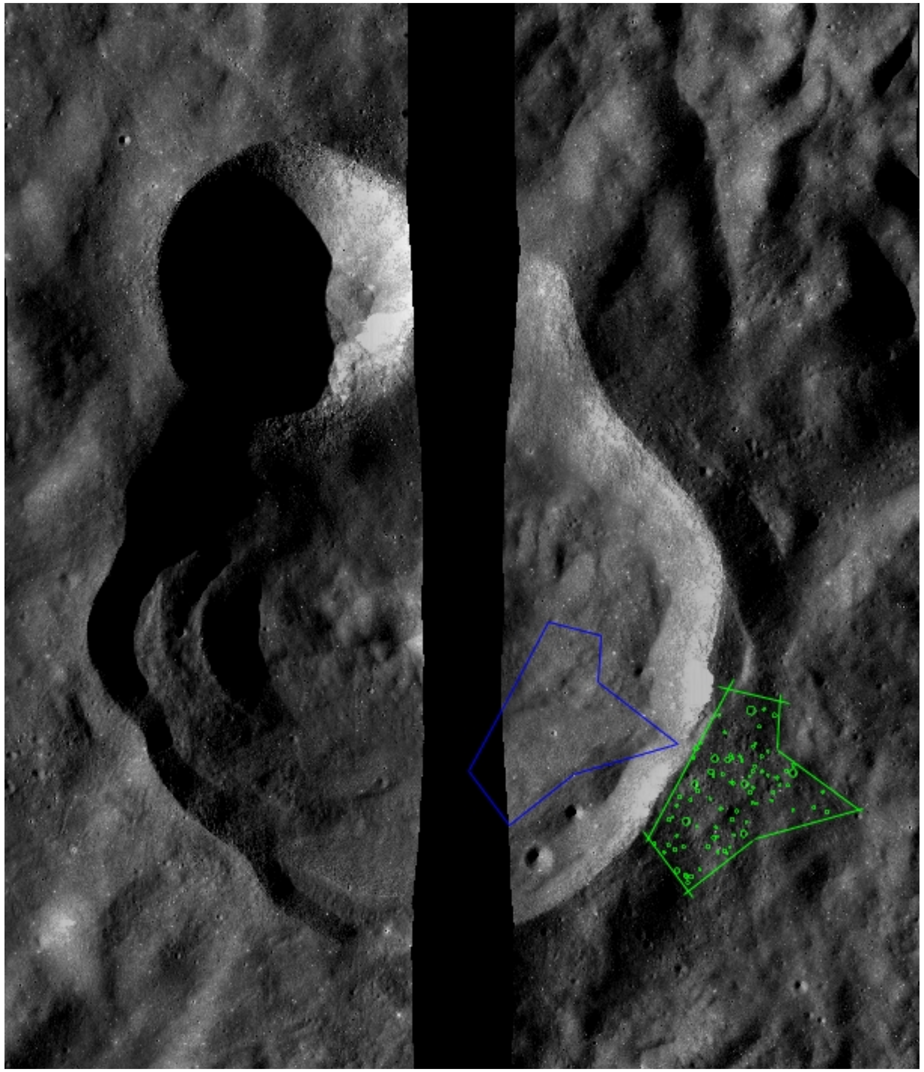

B

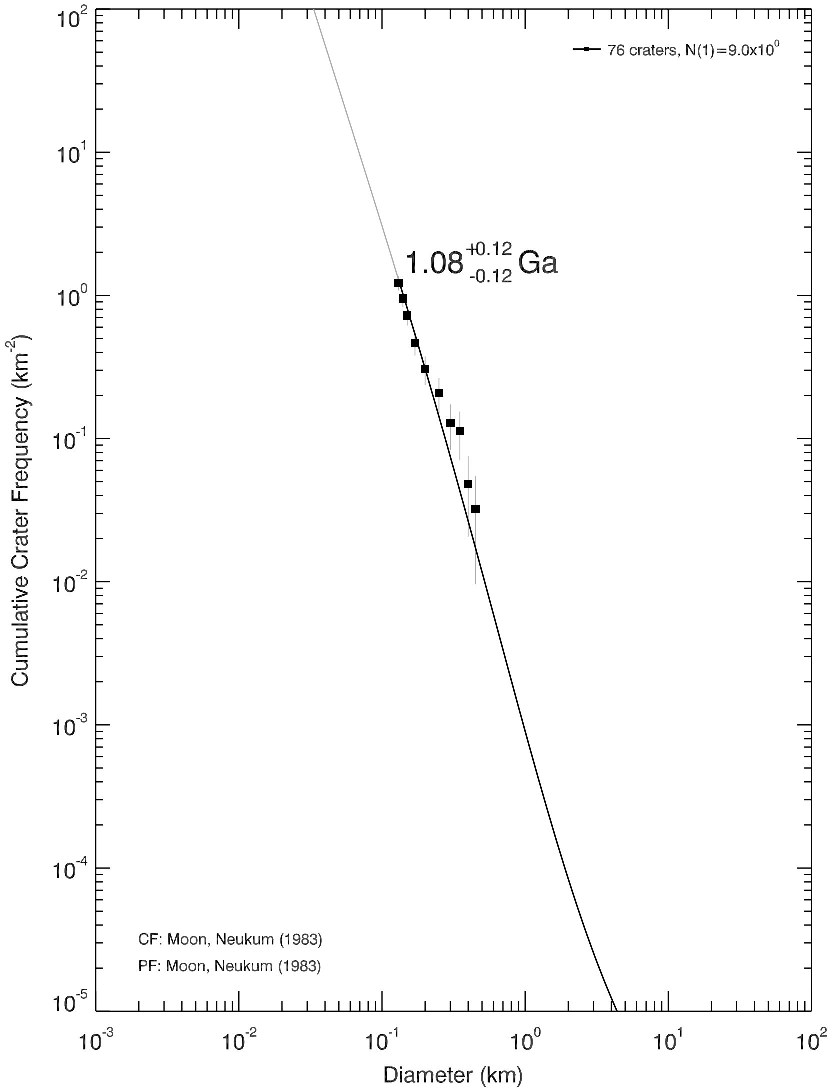

Supplementary Figure 45: The Terrain Camera image and the cumulative size-frequency distribution of Golitsyn

Milne N

Diameter: 32.9 km

Number of craters: 49   Counting area: 330 km^2

N(1)=$9.08 \times 10^{-4} \pm 1.28 \times 10^{-4}$ km^{-2}, N(10)=$2.30 \times 10^{-6} \pm 3.26 \times 10^{-7}$ km^{-2}

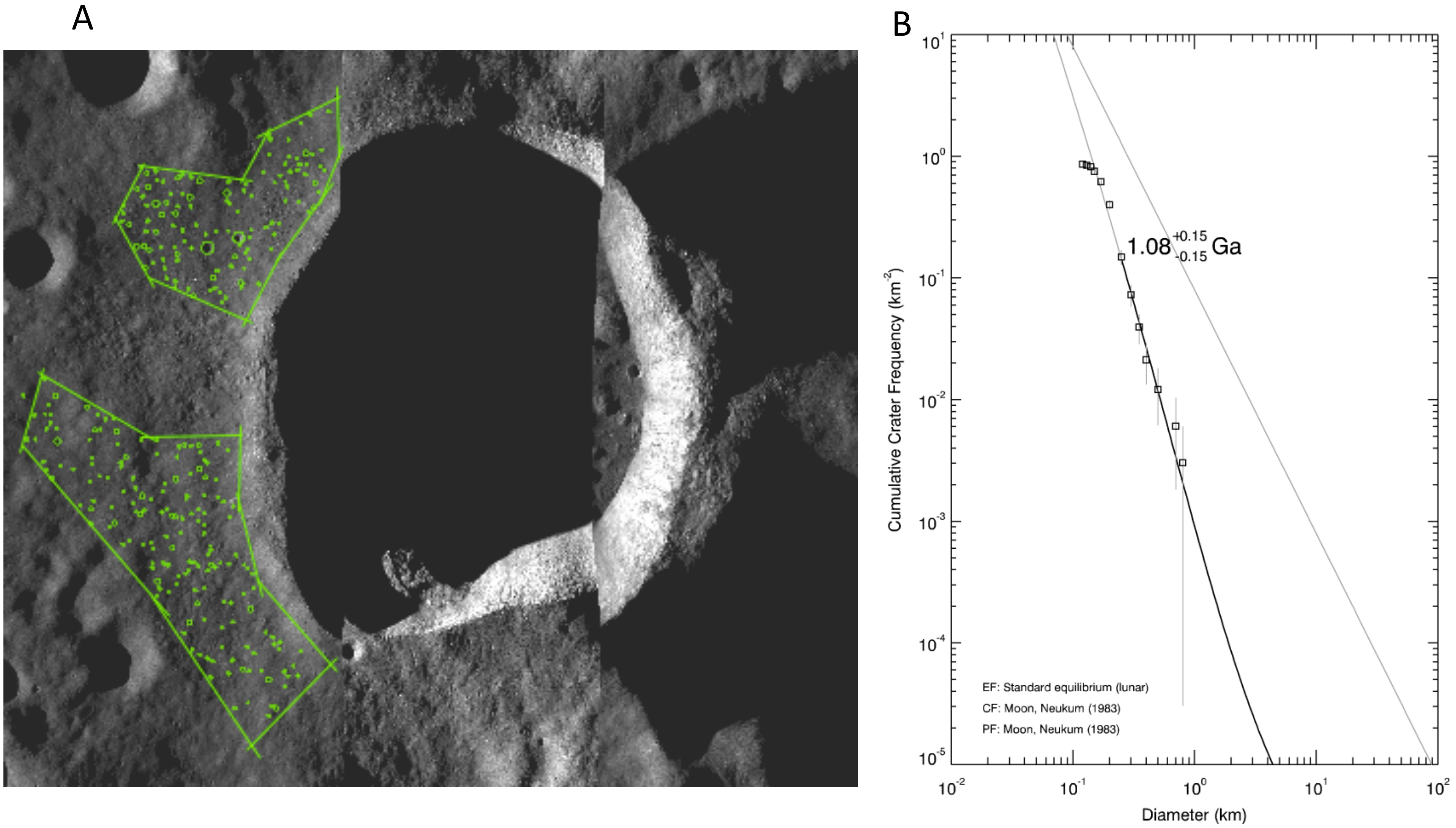

Supplementary Figure 46: The Terrain Camera image and the cumulative size-frequency distribution of Milne N

Guthnic

Diameter: 36.2 km

Number of craters: 183 Counting area: 1140 km^2

$N(1)=9.23\times10^{-4}\pm6.75\times10^{-5}$ km^{-2}, $N(10)=2.34\times10^{-6}\pm1.71\times10^{-7}$ km^{-2}

A B

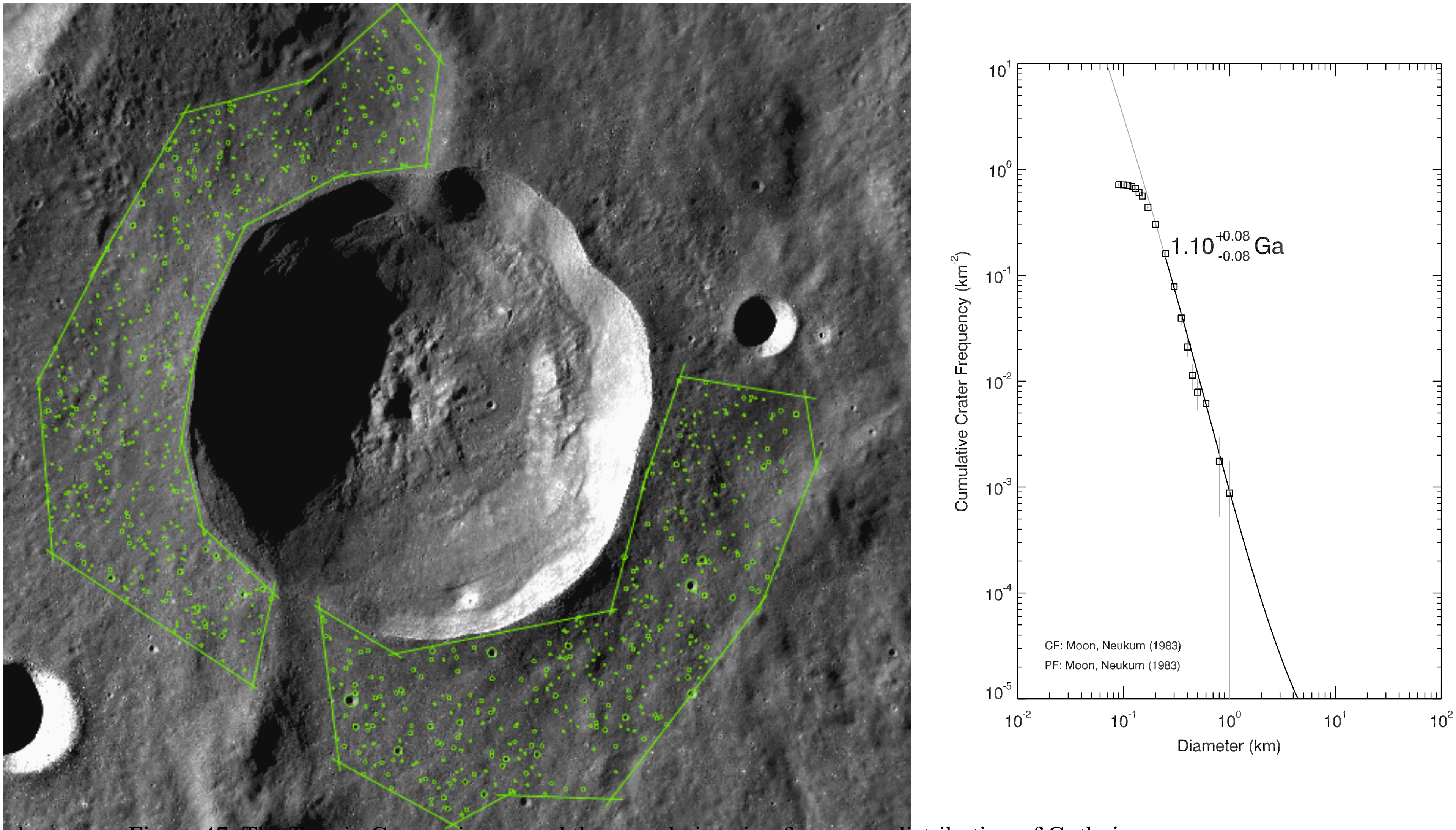

Supplementary Figure 47: The Terrain Camera image and the cumulative size-frequency distribution of Guthnic

Steno Q

Diameter: 32.0 km

Number of craters: 63   Counting area: 430 km^2

N(1)=$9.59 \times 10^{-4} \pm 1.20 \times 10^{-4}$ km^{-2}, N(10)=$2.43 \times 10^{-6} \pm 3.04 \times 10^{-7}$ km^{-2}

A

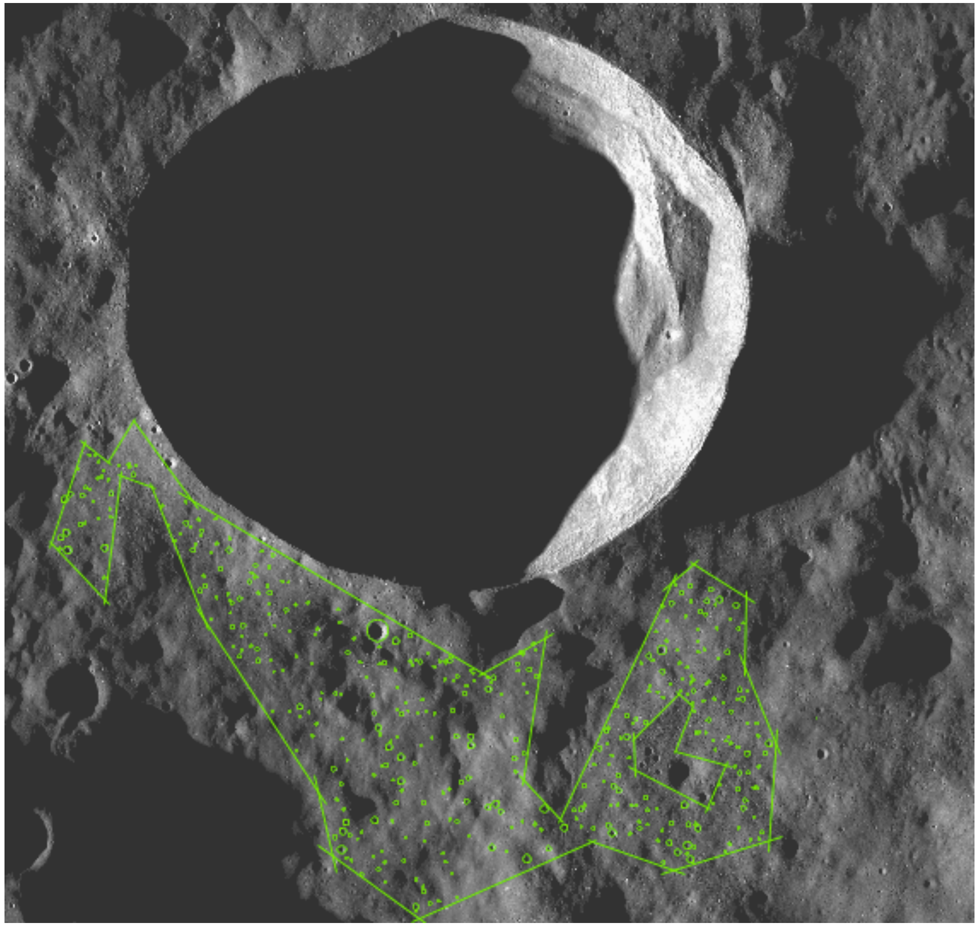

B

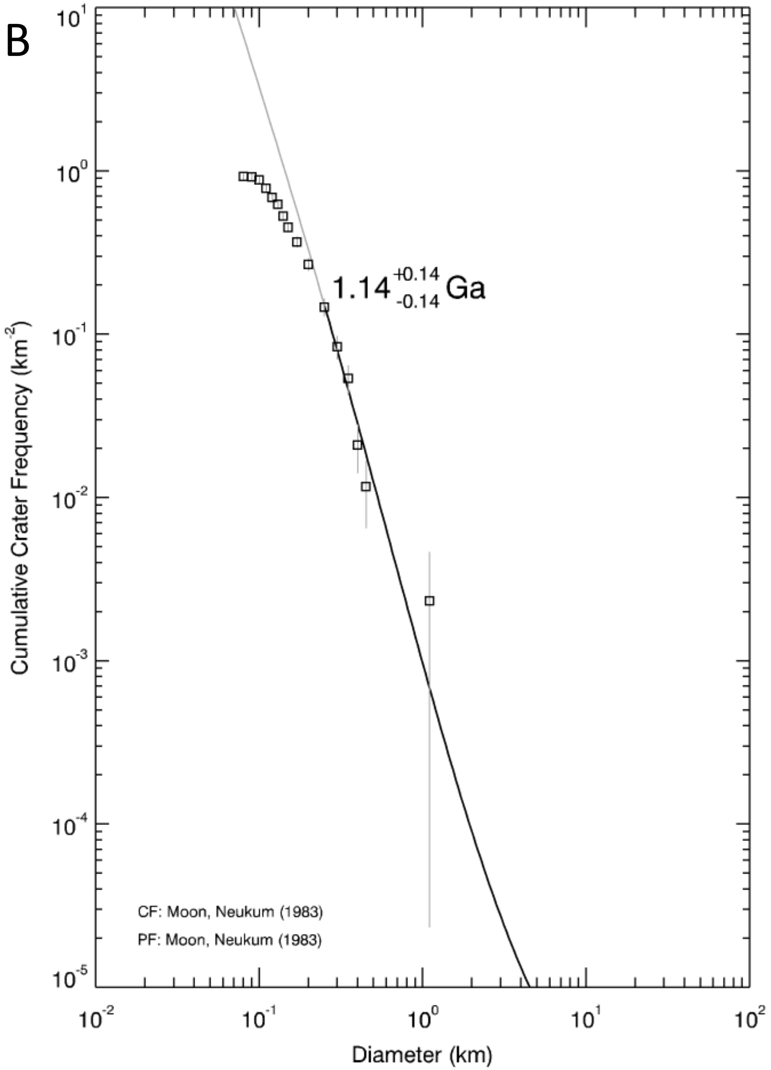

Supplementary Figure 48: The Terrain Camera image and the cumulative size-frequency distribution of Steno Q

Vavilov

Diameter: 96.6 km

Number of craters: 318 Counting area: 4139 km^2

N(1)=$1.06 \times 10^{-3} \pm 5.87 \times 10^{-5}$ km^{-2}, N(10)=$2.68 \times 10^{-6} \pm 1.49 \times 10^{-7}$ km^{-2}

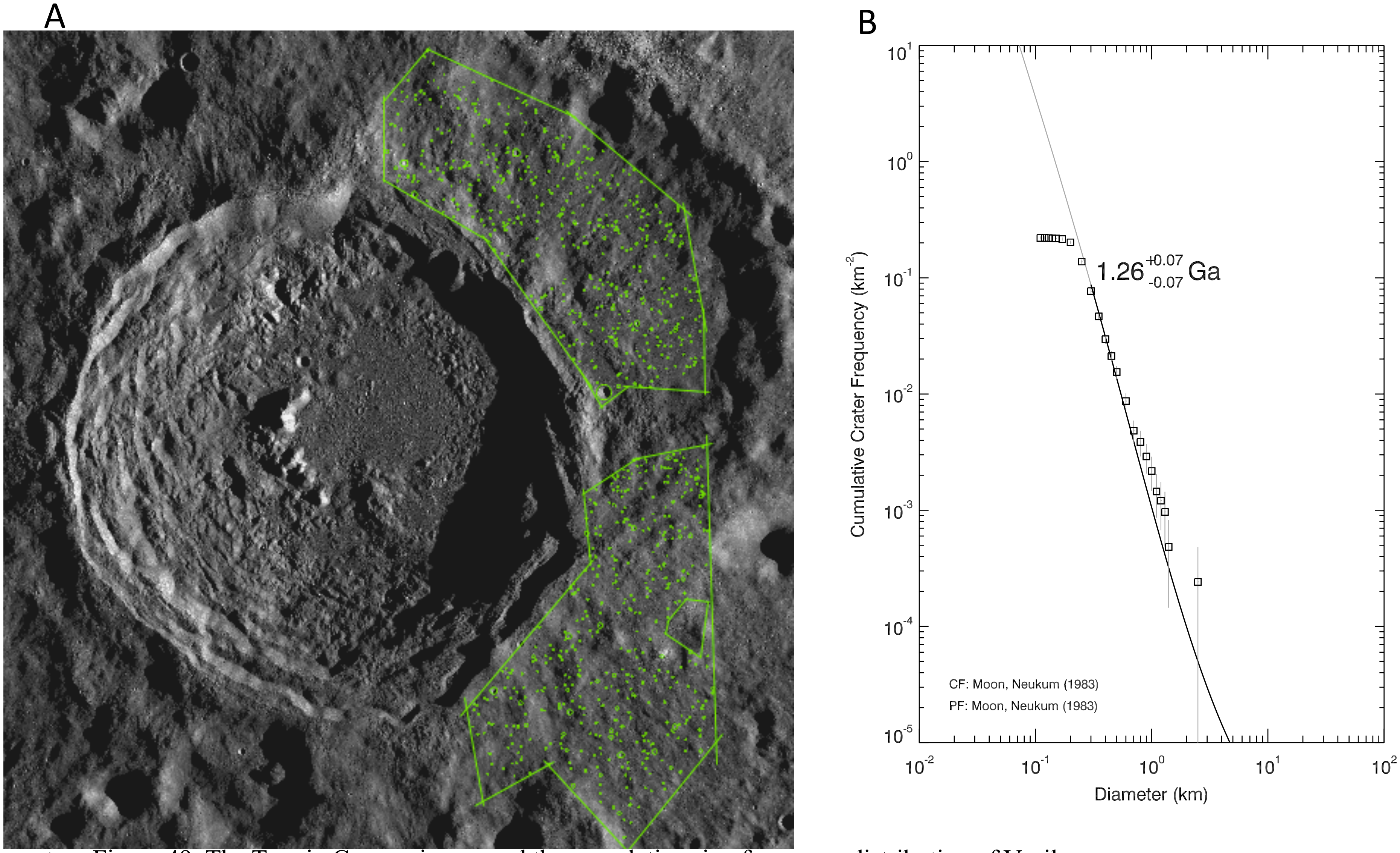

Supplementary Figure 49: The Terrain Camera image and the cumulative size-frequency distribution of Vavilov

Plante

Diameter: 35.3 km

Number of craters: 55 Counting area: 286 km^2

$N(1)=1.15\times10^{-3}\pm1.53\times10^{-4}$ km^{-2}, $N(10)=2.91\times10^{-6}\pm3.88\times10^{-7}$ km^{-2}

A

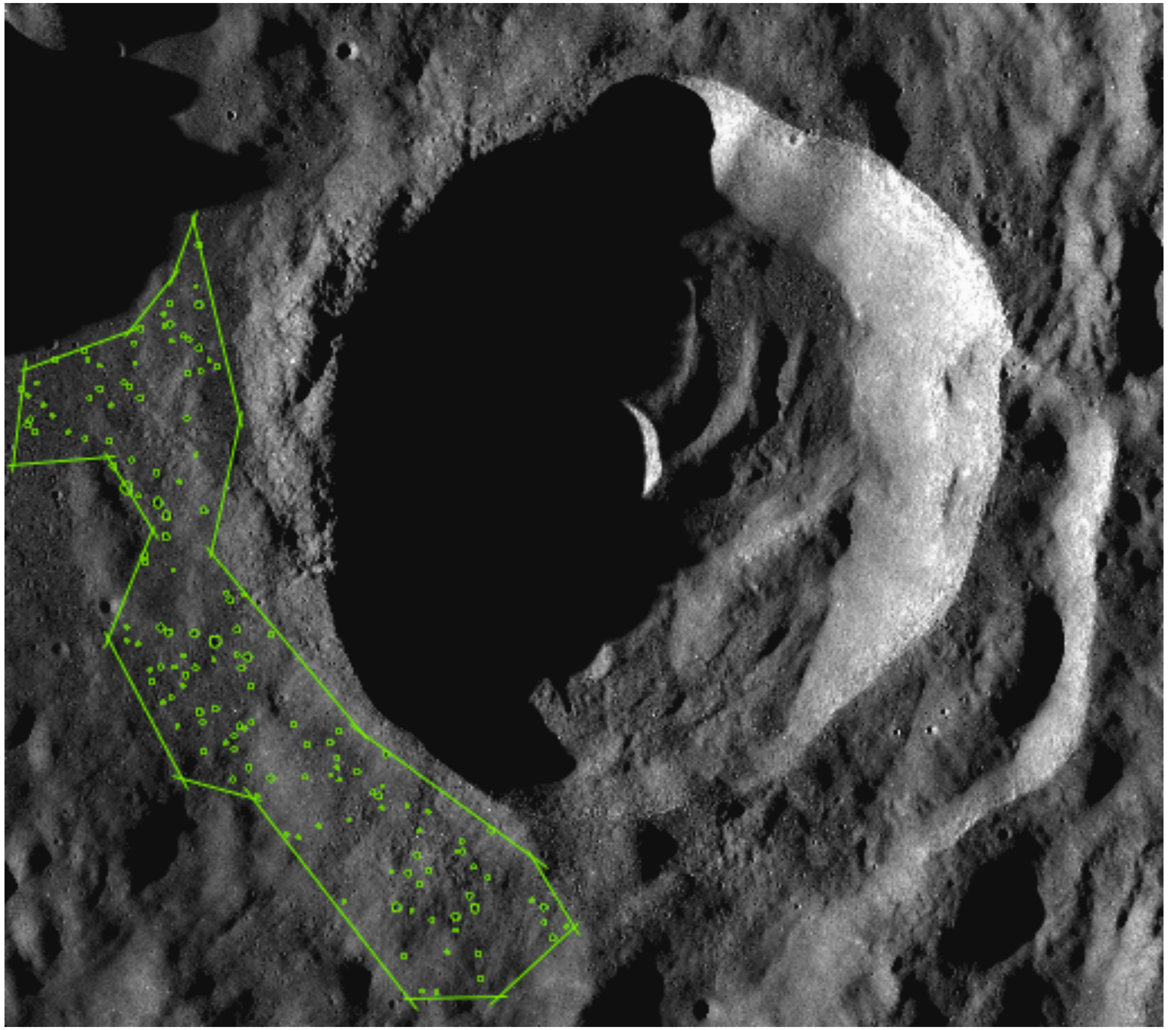

B

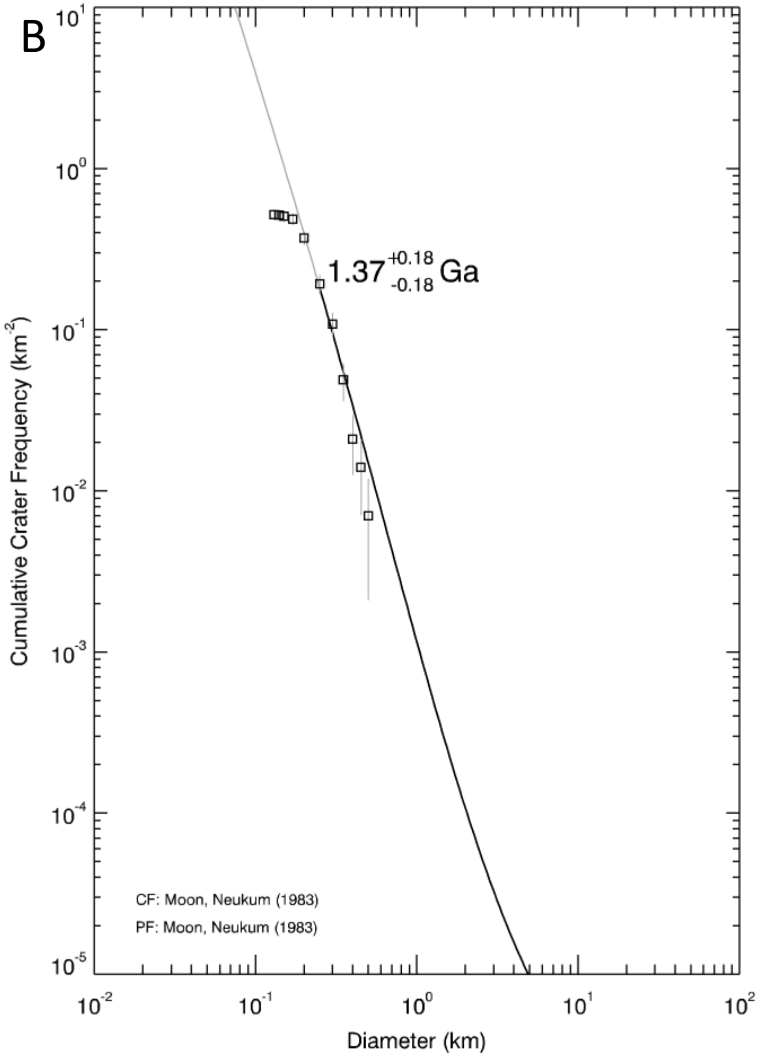

Supplementary Figure 50: The Terrain Camera image and the cumulative size-frequency distribution of Plante

Laue G

Diameter: 29.8 km

Number of craters: 152   Counting area: 819 km^2

N(1)=$1.20\times10^{-3}\pm9.62\times10^{-5}$ km^{-2}, N(10)=$3.04\times10^{-6}\pm2.44\times10^{-7}$ km^{-2}

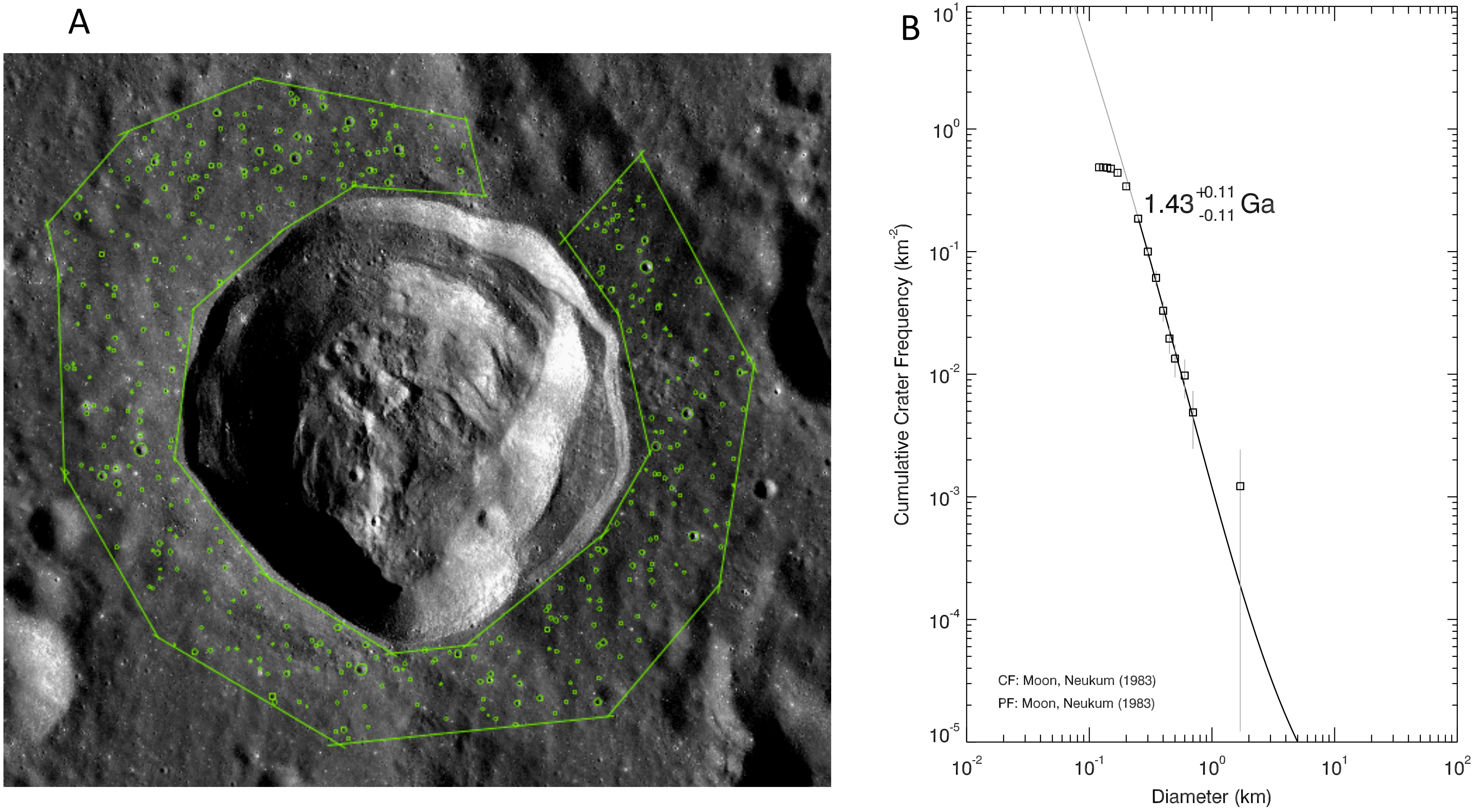

Supplementary Figure 51: The Terrain Camera image and the cumulative size-frequency distribution of Laue G

Focas

Diameter: 22.0 km

Number of craters: 78 Counting area: 325 km^2

N(1)=1.51$\times$10^{-3}$\pm$1.69$\times$10^{-4} km^{-2}, N(10)=3.82$\times$10^{-6}$\pm$4.29$\times$10^{-7} km^{-2}

A

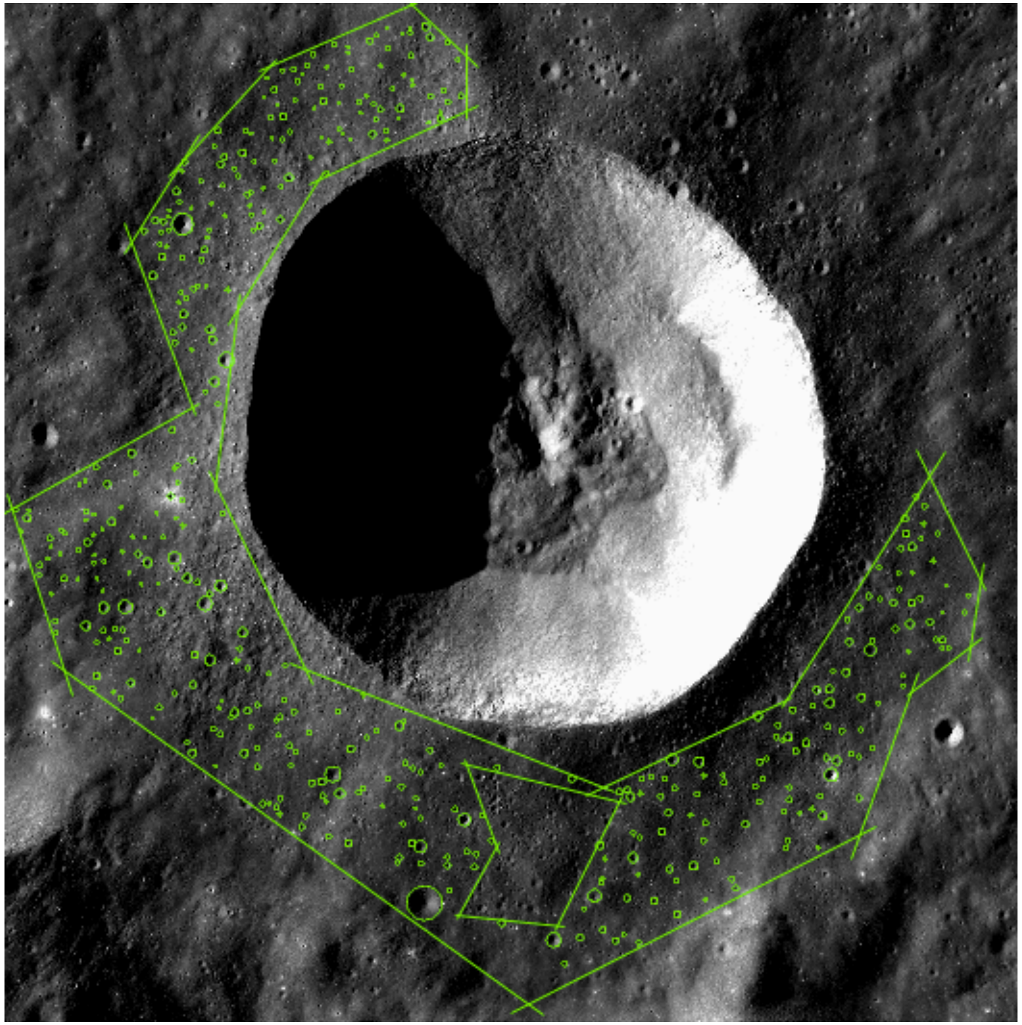

B

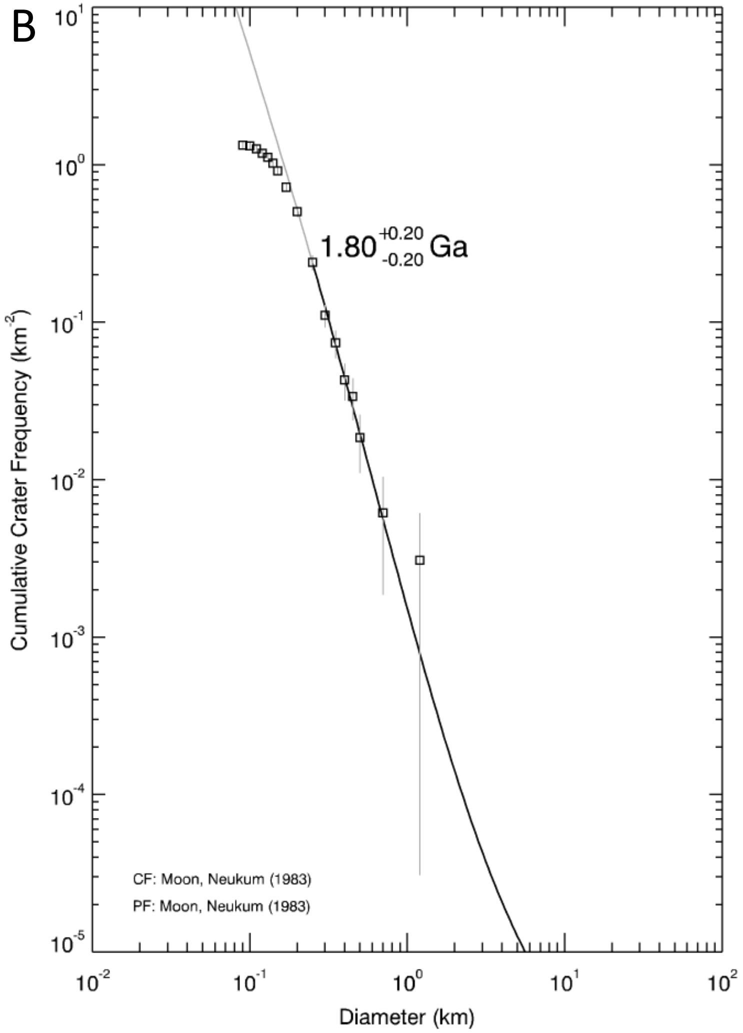

Supplementary Figure 52: The Terrain Camera image and the cumulative size-frequency distribution of Focas

Coriolis Y

Diameter: 31.2 km

Number of craters: 31 Counting area: 183 km^2

N(1)=1.57$\times$10^{-3}$\pm$2.49$\times$10^{-4} km^{-2}, N(10)=3.98$\times$10^{-6}$\pm$6.30$\times$10^{-7} km^{-2}

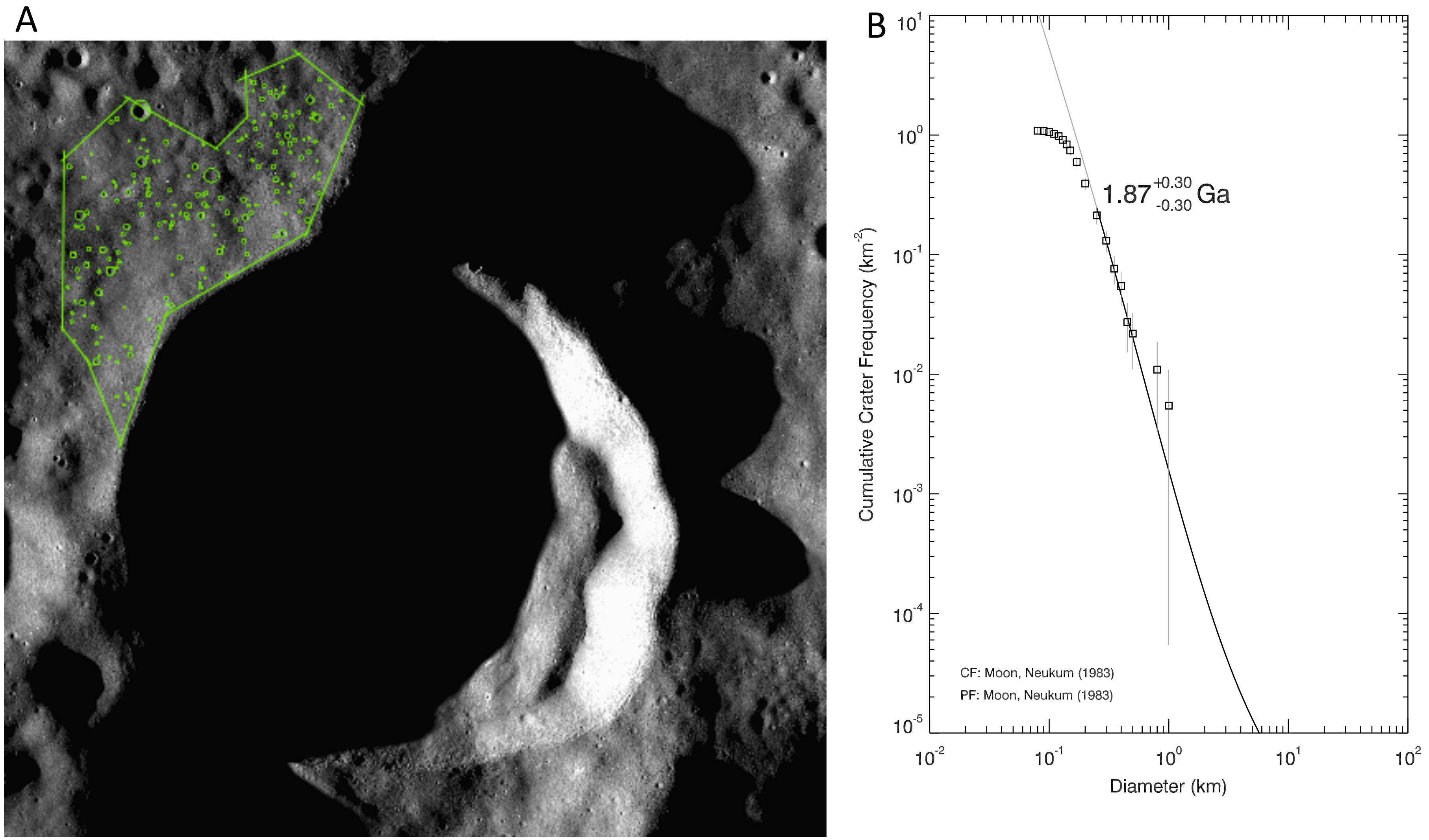

Supplementary Figure 53: The Terrain Camera image and the cumulative size-frequency distribution of Coriolis Y

Golitsyn J

Diameter: 19.0 km

Number of craters: 37 Counting area: 149 km^2

$N(1)=1.65\times10^{-3}\pm2.69\times10^{-5}\ km^{-2}$, $N(10)=4.20\times10^{-6}\pm6.83\times10^{-7}\ km^{-2}$

A

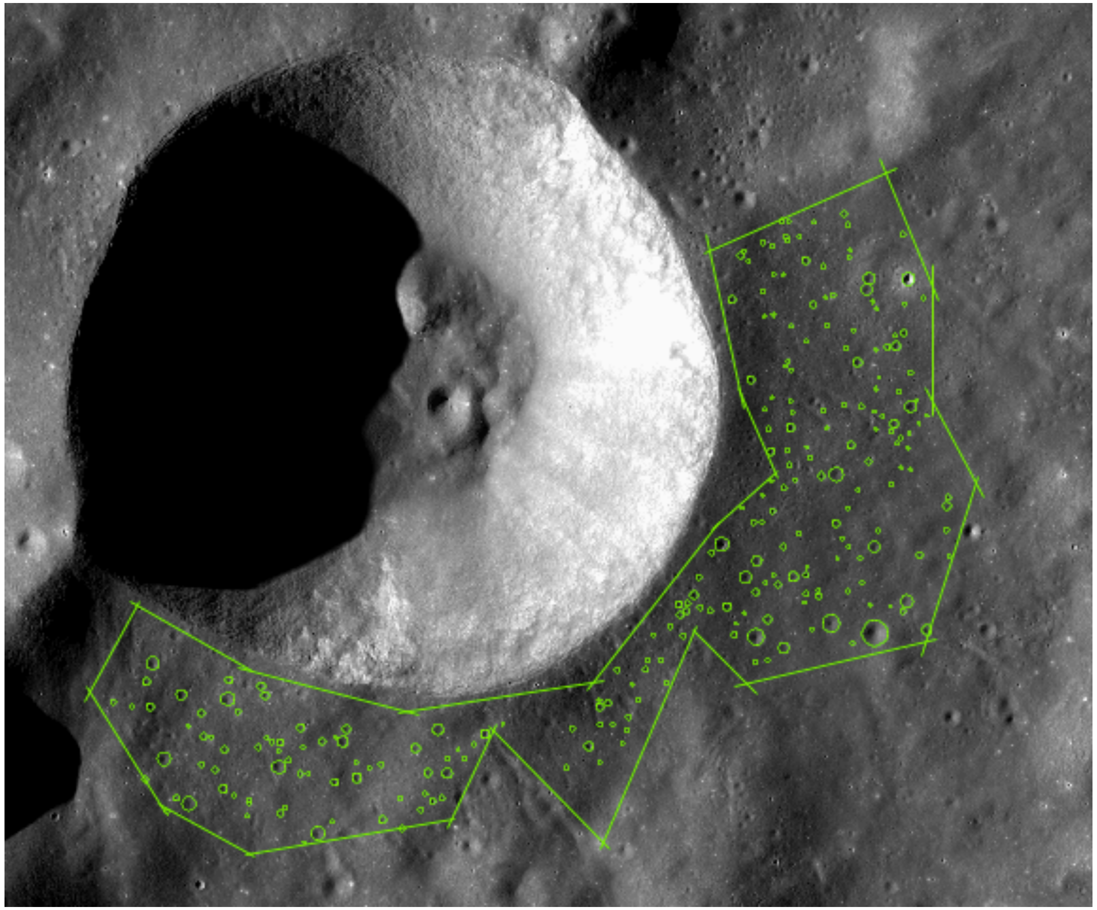

B

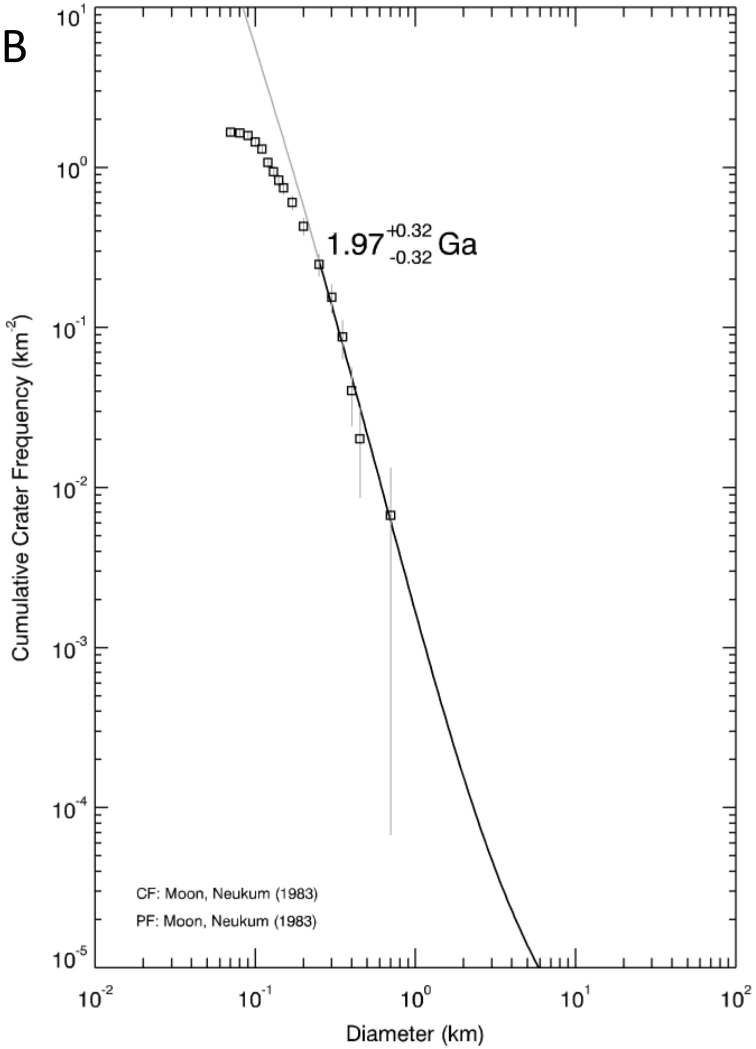

Supplementary Figure 54: The Terrain Camera image and the cumulative size-frequency distribution of Golitsyn J

Green M

Diameter: 34.7 km

Number of craters: 84 Counting area: 357 km^2

$N(1)=1.65\times10^{-3}\pm1.79\times10^{-4}$ km^{-2}, $N(10)= 4.19\times10^{-6}\pm4.53\times10^{-7}$ km^{-2}

A

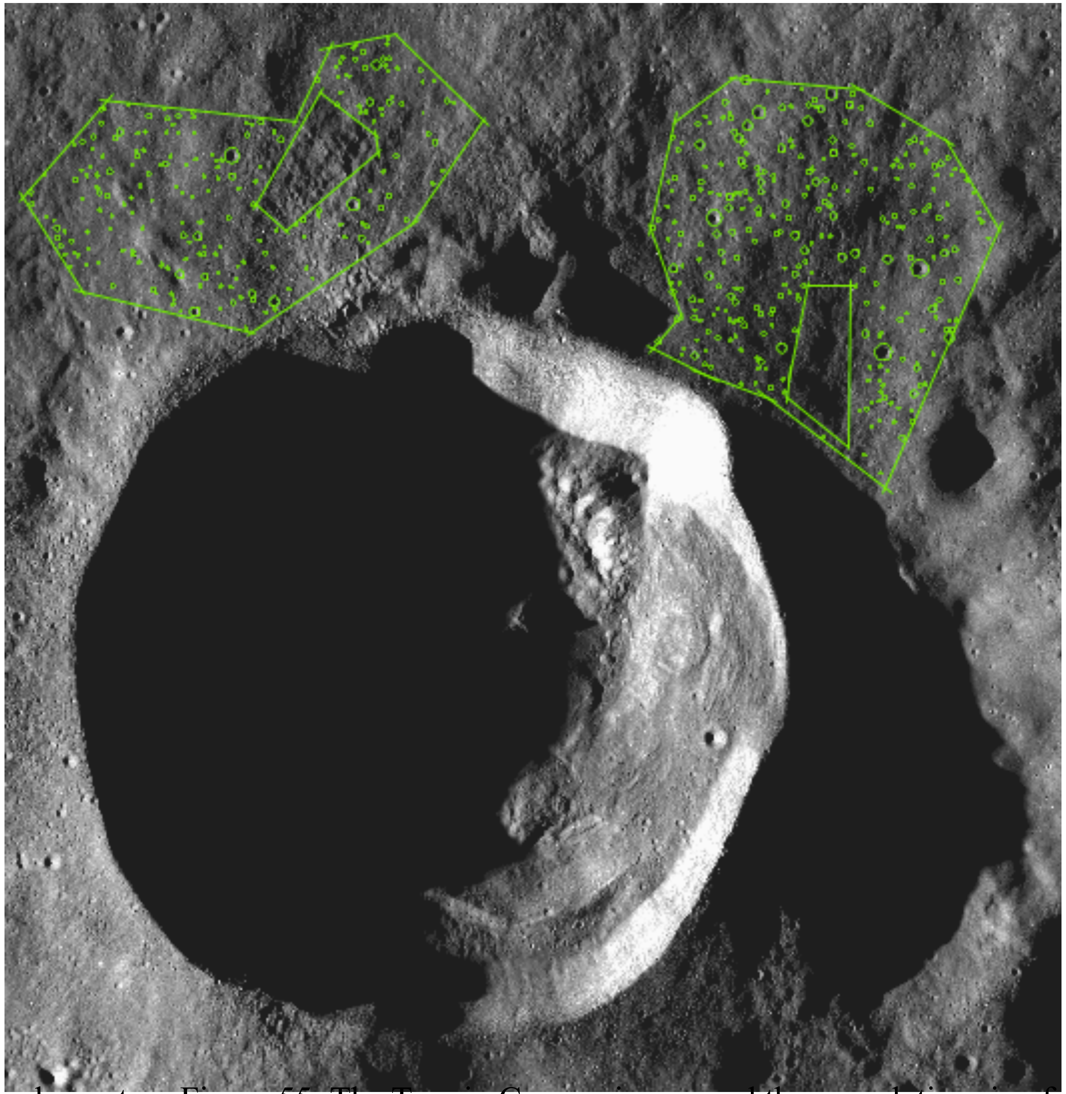

B

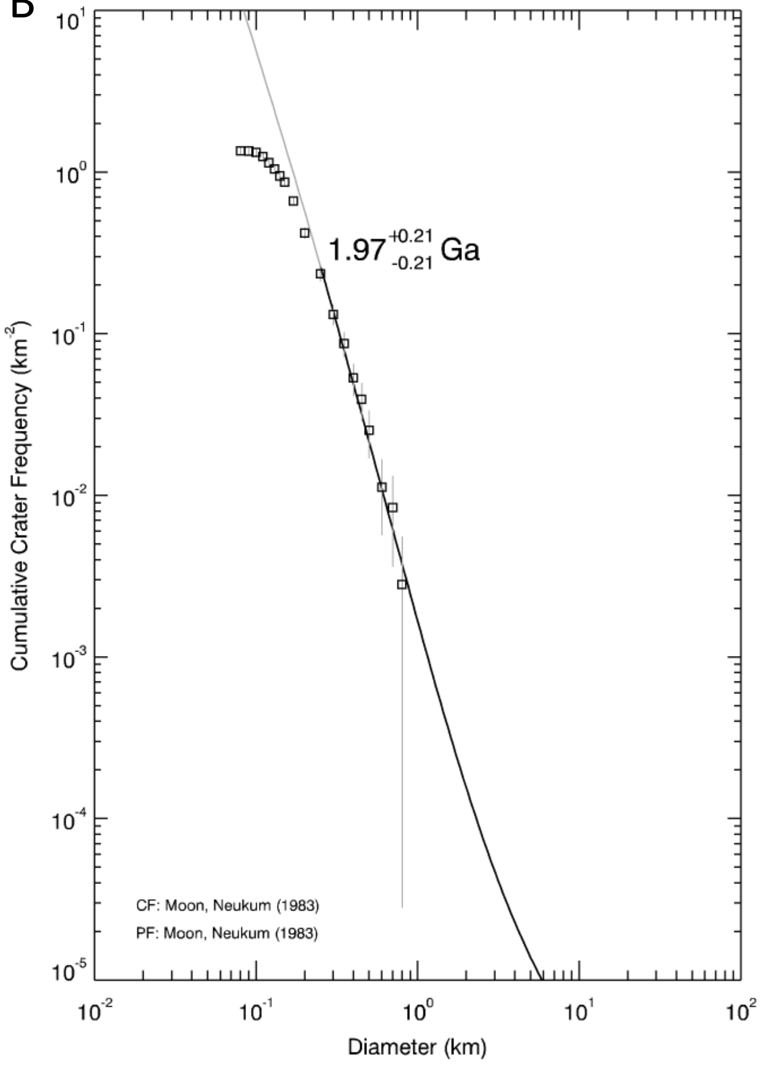

Supplementary Figure 55: The Terrain Camera image and the cumulative size-frequency distribution of Green M

Gerasimovich D

Diameter: 25.9 km

Number of craters: 40 Counting area: 392 km^2

N(1)=1.67$\times$10^{-3}$\pm$3.70$\times$10^{-4} km^{-2}, N(10)=4.24$\times$10^{-6}$\pm$9.39$\times$10^{-7} km^{-2}

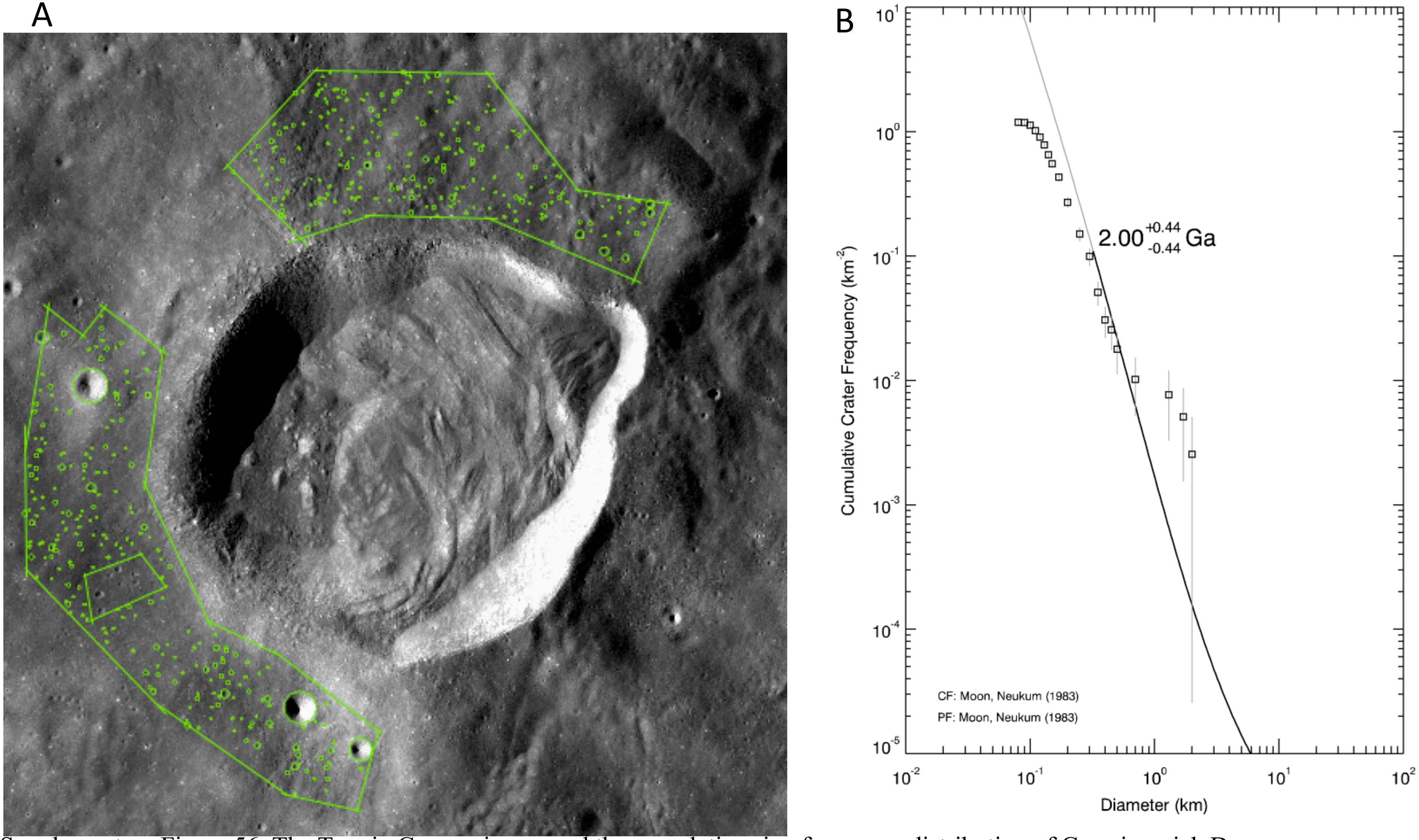

Supplementary Figure 56: The Terrain Camera image and the cumulative size-frequency distribution of Gerasimovich D

Dufay B

Diameter: 21.6 km

Number of craters: 75 Counting area: 264 km^2

N(1)=1.99$\times$10^{-3}$\pm$2.27$\times$10^{-4} km^{-2}, N(10)=5.04$\times$10^{-6}$\pm$5.77$\times$10^{-7} km^{-2}

A B

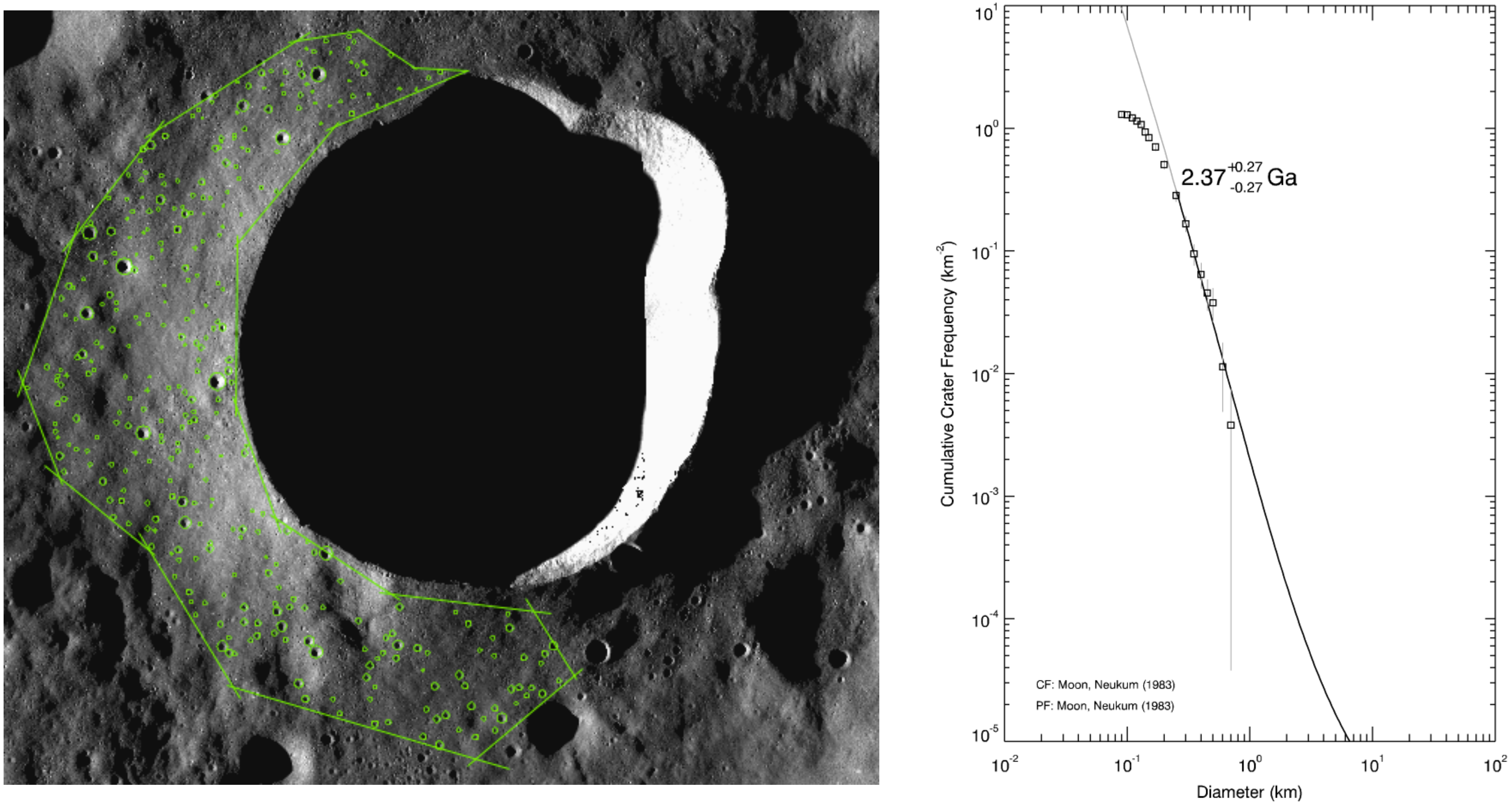

Supplementary Figure 57: The Terrain Camera image and the cumulative size-frequency distribution of Dufay B

Pasteur D

Diameter: 39.3 km

Number of craters: 65   Counting area: 380 km^2

N(1)=1.99$\times$10^{-3}$\pm$2.45$\times$10^{-4} km^{-2}, N(10)=5.06$\times$10^{-6}$\pm$6.21$\times$10^{-7} km^{-2}

A

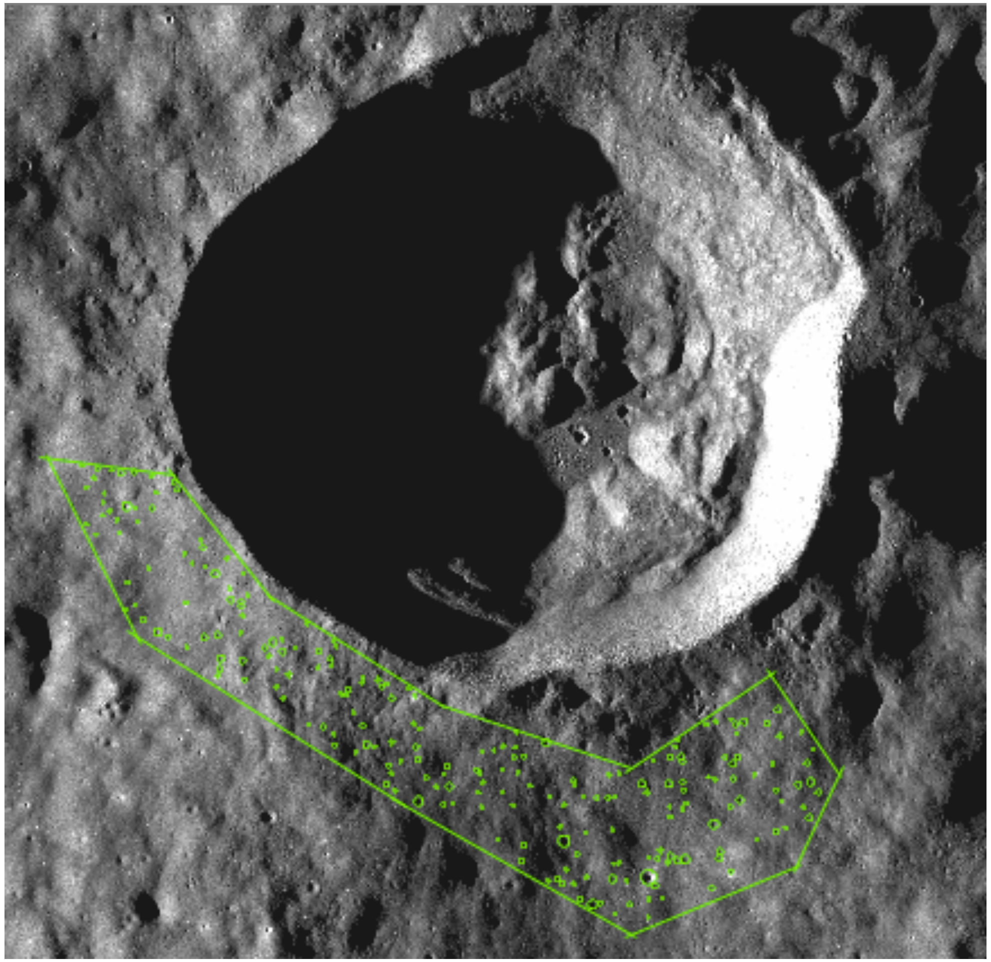

B

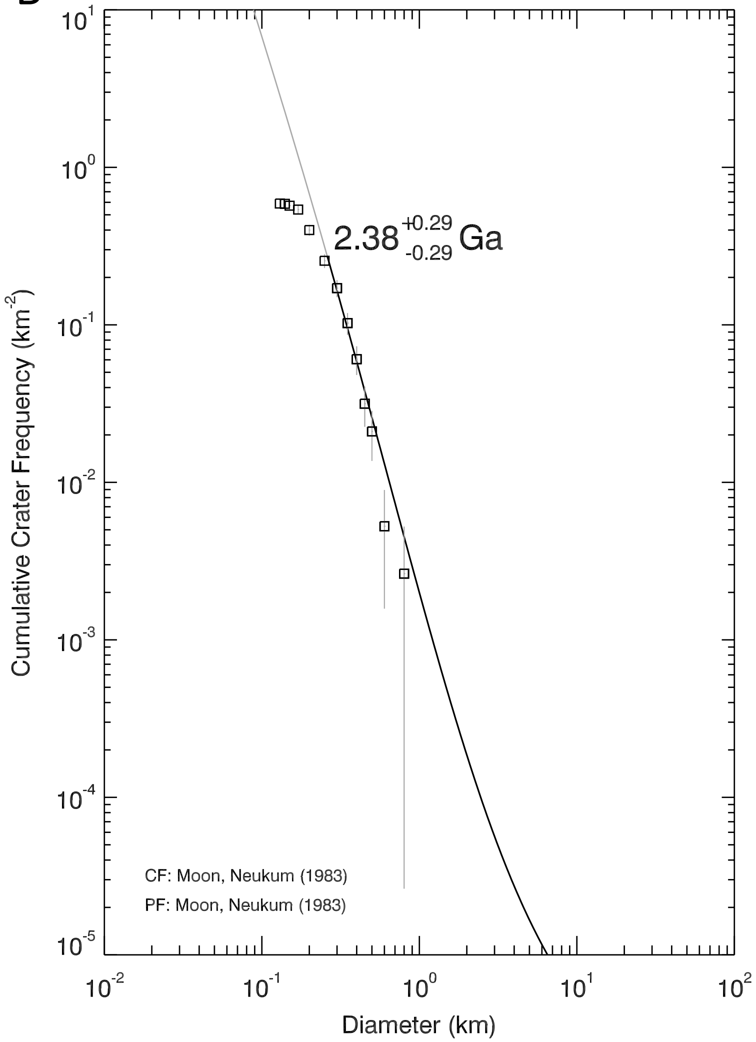

Supplementary Figure 58: The Terrain Camera image and the cumulative size-frequency distribution of Pasteur D

34S 130W

Diameter: 19.4 km

Number of craters: 2 Counting area: 141 km^2

N(1)=2.07$\times$10$^{-3}\pm$1.45$\times$10^{-4} km^{-2}, N(10)=5.26$\times$10$^{-6}\pm$3.68$\times$10^{-6} km^{-2}

A

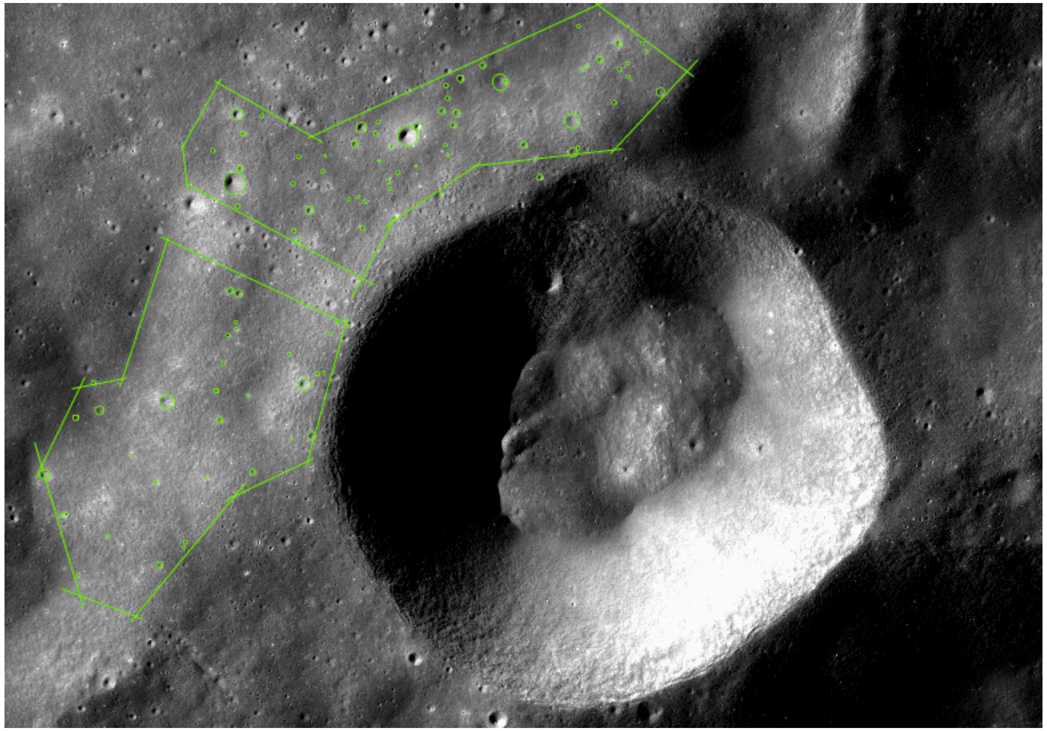

B

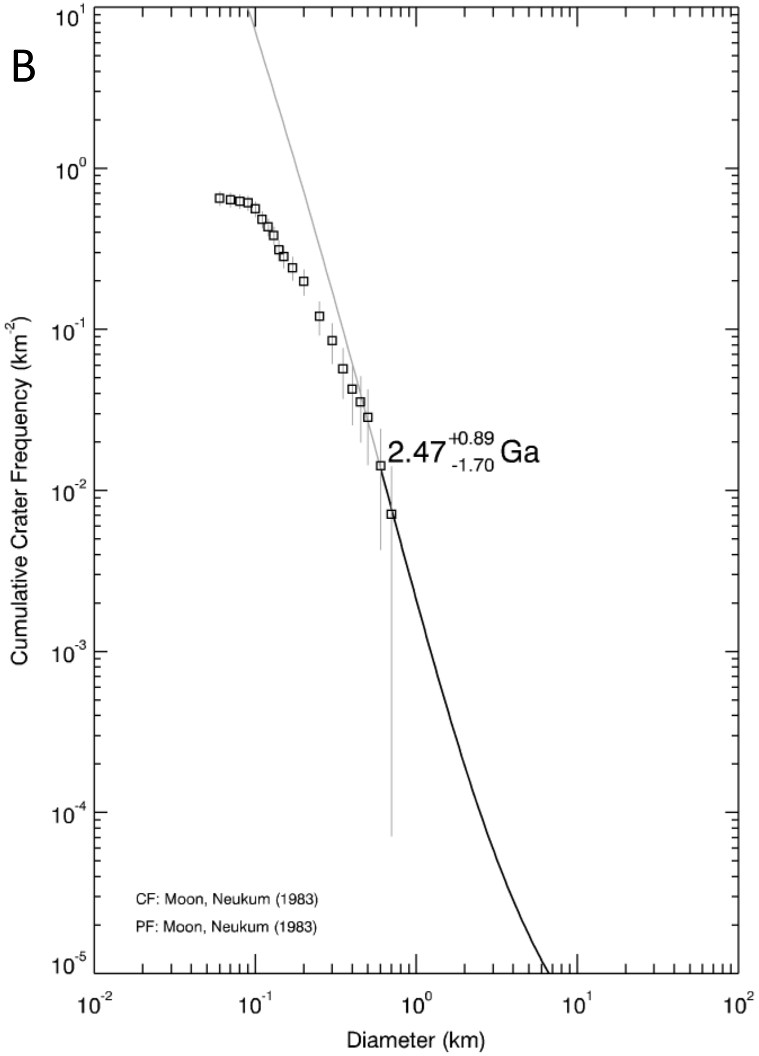

Supplementary Figure 59: The Terrain Camera image and the cumulative size-frequency distribution of 34S 130W
